# Supplementary material for: Perceived facilitators and barriers among physical therapists and orthopedic surgeons to pre-operative home-based exercise with one exercise-only in patients eligible for knee replacement: A qualitative interview study nested in the QUADX-1 trial
Source: PLoS One. 2020 Oct 23;15(10):e0241175. doi: 10.1371/journal.pone.0241175 (PMC7584251; doi:10.1371/journal.pone.0241175)
Supplement: S2 File — (PDF) [file pone.0241175.s002.pdf]

Appendix 2 – Interview guide physiotherapists

Interview guide physiotherapists

Therapist information

Age
Municipality

1. **The role of the physiotherapist in this type of exercise therapy intervention (exercise instruction followed by home-based exercise)**
   a. What kind of role do you think you will have in this type of physiotherapy?
      i. Advantages, disadvantages or possibilities?
   b. Reflect on how this home-based/self-management approach is different from your current treatment of these patients.
      i. Advantages, disadvantages or possibilities? Time?

   <u>Supplementary questions if topics not discussed from the above</u>
   c. Do you experience that you lose professionalism when instructing patients and send them home compared to supervising their treatment (exercise) and the quality of the treatment?
      i. Advantages and/or disadvantages of this un-supervised type of physiotherapy?
   d. Can more time by allocated to patients with higher need when patients with knee osteoarthritis exercise at home?
   e. Does this approach change your role in relation to motivating patients to exercise?
      i. E.g. adherence to home-based exercise/"sell the idea" (pedagogy)?
      ii. Does this role acquire other skills from you? If so, which?

2. **Pre-operative exercise**
   a. Which possibilities or barriers do you see in relation to exercise therapy in patients with end-stage knee osteoarthritis?
      i. Advantages and disadvantages for both physiotherapists and patients?

3. **Non-supervised exercise therapy**
   a. Which possibilities and barriers do you see in relation to non-supervised exercise therapy?
      i. Advantages, disadvantages why?
   b. To what extent do you think that the patients can get the same quality in the exercise therapy when you are not there to supervise them?
      i. Why, why not?

   <u>Supplementary questions if topics not discussed from the above</u>
   c. Are the patients able to adhere to the pre-scribed exercise dosage when not supervised?
      Why, why not? Elaborate.
   d. Are the patients able to exercise with the same quality non-supervised as supervised?
      i. Why, why not? Elaborate.

4. **One-exercise exercise therapy**
   a. Reflect on the rehabilitation approach with *one* exercise. How does this deviate you're your current practice?
      i. Advantages, disadvantages to this approach?
   b. Reflect on the effect of exercise therapy with *one* exercise. What does it require to achieve effect?

i. Why?<br>c. What do you think the patient's thoughts are on this?<br>i. Why?
**5. The model of coordinated non-surgical and surgical treatment**<br>a. What are your thoughts on the model where the coordination between municipality and hospital is initiated before potential surgery?<br>i. Which advantages, disadvantages or possibilities do you see in the model? Both to yourselves and for the patients?<br>b. Is the model of coordinated non-surgical and surgical treatment applicable to your daily clinical practice?<br>i. If not, what would need to be changed for it to be applied?<br>c. What are your thoughts on the model of coordinated non-surgical and surgical treatment on an organizational level?<br>i. Advantages, disadvantages or possibilities? Is it implementable?
**6. Other**<br>a. Topics we have not discussed which you would like to comment on?
