# Supplementary material for: Perceived facilitators and barriers among physical therapists and orthopedic surgeons to pre-operative home-based exercise with one exercise-only in patients eligible for knee replacement: A qualitative interview study nested in the QUADX-1 trial
Source: PLoS One. 2020 Oct 23;15(10):e0241175. doi: 10.1371/journal.pone.0241175 (PMC7584251; doi:10.1371/journal.pone.0241175)
Supplement: S3 File — (PDF) [file pone.0241175.s003.pdf]

Appendix 3 – Interview guide orthopedic surgeons

Interview guide orthopedic surgeons

Surgeon information

Age

1. The model of coordinated non-surgical and surgical treatment prior to surgery

   a. What are your thoughts on referring candidates for total knee arthroplasty to non-surgical treatment in the form of exercise therapy prior to potential surgery (the model)?
      i. Which advantages, disadvantages or possibilities do you see in this coordination of non-surgical and surgical treatment? Both to yourself and to the patients.
   b. Can this coordination of non-surgical and surgical treatment (the model) be adapted to your clinical practice?
      i. If not, what should be changed for it to be adapted?
   c. What are your thoughts on the coordination of non-surgical and surgical treatment (the model) on an organizational level, that is, who is responsible for running it?
   d. Advantages, disadvantages or possibilities? Can it (the model) be implemented?
   e. What do you consider the most important factor we should aware of in this project?

2. The role of orthopedic surgeons in the pre-operative coordination of non-surgical and surgical treatment

   a. What role do you think you as orthopedic surgeons will have in this coordination of non-surgical and surgical treatment?
      i. Advantages, disadvantages or possibilities?
   b. What type of patients (characteristics) do you consider good candidates to try exercise therapy prior surgery?
      i. Why, why not?
   c. Are there patients whom you do not think will benefit from exercise therapy but who should undergo surgery right away?
      i. Why, why not?
   d. Which barriers do you see in relation to systematically using exercise therapy in the treatment of patients with knee osteoarthritis?

3. Re-evaluation of the most optimal treatment on a better basis (shared decision making)

   a. The purpose of the project is that the evaluation of the most optimal treatment for the patient is based on the best possible basis, that is, that non-surgical treatment has been tried prior to surgery. Do you think this will provide you with a more optimal basis in relation to whether surgery is the best treatment or not?
      i. Why, why not? Advantages, disadvantages or possibilities?
      ii. What do you think the patients' thoughts are on this?

4. Self-management

   a. As part of the non-surgical treatment the patients also receive tools (self-managed exercise therapy and pain management) to manage their knee related symptoms? What are your thoughts on this education of the patients to better self-manage their condition?
      i. Advantages, disadvantages or possibilities?

5. Potentially better rehabilitation

a. If the exercise therapy is effective some patients might postpone their surgery, while those who undergo surgery likely will have an easier rehabilitation. What are your thoughts on these two scenarios?<br>i. Advantages, disadvantages or possibilities?
**6. Other**<br>a. Topics we have not discussed which you would like to comment on?
