# Supplementary material for: Perceived facilitators and barriers among physical therapists and orthopedic surgeons to pre-operative home-based exercise with one exercise-only in patients eligible for knee replacement: A qualitative interview study nested in the QUADX-1 trial
Source: PLoS One. 2020 Oct 23;15(10):e0241175. doi: 10.1371/journal.pone.0241175 (PMC7584251; doi:10.1371/journal.pone.0241175)
Supplement: S4 File — (PDF) [file pone.0241175.s004.pdf]

Supplementary file (S4 File) – Adjustments and additions to the semi-structured interview guides

Orthopedic surgeons:

Based on previous interviews the following topics were continuously added to the semi-structured interview guide for the single interviews with the orthopedic surgeons.

- Economic evaluations of non-surgical and surgical treatment. E.g. cost-benefit in the short and long term of non-surgical and surgical treatment.
- Perspectives on structuring coordination of non-surgical and surgical treatment. E.g. benefits and disadvantages of referring to exercise at the hospital (in-house) in stead of referring to exercise in the municipality.
- Orthopedic surgeon's individual criteria for selecting patients for either surgical or non-surgical treatment. E.g. more information on the individual orthopedic surgeon's reasons to refer to either surgical or non-surgical treatment.
- The importance of motivation towards exercise among the patients and concomitant compliance to exercise. E.g. whether patient motivation influence referral to i.a. exercise treatment.

Physical therapists:

Based on notes of topics important to pursue the semi-structured interview guide for the focus group interview with the physical therapists was adjusted in the two breaks during the interview to ensure that these were pursued later in the interview.

- Teaching patient's self-management of their condition after supervised treatment. Both related to exercise specific matters but also ensuring that patients has a better understanding of their condition and how to manage symptoms.
- Motivating patients to continue home-based exercise (self-managed) after supervised exercise.
- A physical therapist describes patients today as being more independent than e.g. twenty years ago. Meaning that patients are more likely to do what they feel is right regardless of recommendations from e.g. physical therapists. How does this affect the physical therapist-patient relationship and dynamics in the encounter with patients?
