# Supplementary material for: Perceived facilitators and barriers among physical therapists and orthopedic surgeons to pre-operative home-based exercise with one exercise-only in patients eligible for knee replacement: A qualitative interview study nested in the QUADX-1 trial
Source: PLoS One. 2020 Oct 23;15(10):e0241175. doi: 10.1371/journal.pone.0241175 (PMC7584251; doi:10.1371/journal.pone.0241175)
Supplement: S5 File — (PDF) [file pone.0241175.s005.pdf]

Supplementary information (S5 File) - Audit trail of the thematic analysis and a section on trustworthiness

Audit trail of the inductive thematic analysis					
**Analytical step**	**1. Familiarizing with data**	**2. Generating initial codes**	**3. Searching for themes**	**4. Reviewing and defining themes**	**5. Defining and naming themes**
**Analytical process**	Notes on potential facilitators and barriers were written down while listening to the audio recordings.<br><br>During the subsequent reading of the transcripts notes on potential facilitators and barriers (meaning units) were written in the margin of the document.<br><br>The purpose of the first listening and reading of the data was for the primary researcher to familiarize and immense himself with the data. This was considered important to ensure an understanding of the depth and breadth of the content despite that the primary researcher also conducted the interviews.	Coding of meaning units and labelled with initial names.<br><br>Initial codes are based on quotes from the informants or interpreted as a potential facilitator or barrier by the primary investigator.<br><br>Then the coded material was read through again and condensed to a coherent whole and each provided with headings describing the content.<br><br>Example:<br><br>Heading: Orthopedic surgeons and exercise;<br><br>"*Well, for me, as a profession, we must at least have a little belief in its (exercise) effectiveness before we*	Based on the initial codes the research group discussed potential sub-themes and themes.<br><br>During this process the codes were put together in initial sub-themes and themes. This was done in an iterative process where different combinations of codes were discussed to obtain the most fair and comprehensive sub-themes and themes.<br><br>Examples of some of the initial themes and sub-themes were:<br><br>Theme:<br>1) Physical therapists' and orthopedic surgeons' ambivalence in their professional roles.	The codes for each preliminary theme were reviewed to reconsider if they formed a coherent pattern supporting the theme. This was done to ensure validity of each theme reflecting meanings evident in the data as a whole. During this process the names of the themes changed.<br><br>For an example the preliminary theme named "Physical therapists' and orthopedic surgeons' ambivalence in their professional roles" was revised and spilt into two new themes named "Physical therapists' dilemma with *one* home-based exercise" and "Orthopedic surgeons' dilemma with exercise".	The final themes and associated sub-themes were agreed upon based the identified facilitators and barriers. Based on the dilemmas these facilitators and barriers created in the professional role of both the physical therapists and orthopedic surgeons the final themes were named.

		refer patients to it, when we have the other alternative (surgery)."<br><br>In this phase the research group discussed whether we interpreted the coding similarly. For an example, the primary investigator was very familiar with the context as he was also the primary investigator on the clinical trial but could also oversee topics due to his preconceptions. For an example, the primary investigator, as a trained physical therapist, oversaw topics related to the professional role as a physical therapist as these seemed given to him.	2) Orthopedic surgeons view on exercise<br><br>Sub-themes:<br><br><u>Orthopedic surgeons:</u><br>1) Criteria for selecting a patient for KR or exercise therapy.<br>2) Orthopedic surgeons are gatekeepers for which patients are referred to exercise therapy.<br>3) Orthopedic surgeons view on exercise therapy.<br>4) Effect of exercise therapy can help guide the decision on surgical treatment.<br><br><u>Physical therapists:</u><br>1) Passive role as physical therapist.<br>2) Thoughts on exercise therapy with *one* exercise.<br>3) Thoughts on home-based/unsupervised exercise therapy.<br>4) Self-management	The preliminary theme named "Orthopedic surgeons view on exercise" was removed and the associated sub-themes linked to the new theme "Orthopedic surgeons' dilemma with exercise".	

			After discussions in the research group some of the sub-themes and themes changed.		
**Analysis performed by**	RSH.	RSH, TB and JK.	RSH, TB and JK.	RSH, TB and JK.	Whole research group.
**Analytical tools**	Both physical and digital versions of the transcripts.	Transcribed interviews.	Coded transcripts.	Preliminary themes and sub-themes.	Reviewed themes and sub-themes.

Trustworthiness in the applied qualitive research process

Credibility, transferability, dependability and confirmability was ensured via the following steps [1–3]:

Credibility:

- Data was gathered and analysed using recognised methodology. Focus group and single interviews were based on semi-structured interview guides [4–7] and data was analysed using inductive thematic analysis [1].
- Reflexions during the analysis process. During the analytical process various preliminary codes/themes were written down and presented to the research group. In some situations, help was needed to ensure that the meaning from a quote was correctly understood in the context of the study. Other examples where related to the interpretation of the latent content of in a quote. Here the preconceptions of the primary investigator could cloud the underlying meaning and other members of the research team asked to challenge the primary investigators interpretation. Finally, discussion of the appropriateness of some quotes were needed in the research group as some members found some quotes inappropriate. For an example, the "[provided as an analogy]" was added to the quote "*You can take the horse to water, but you can't make it drink [provided as an analogy]. It might be that I refer the patient to exercise and that I insist on it. But if the patient comes back three months later and says, "I have not been exercising, now I would like an operation", then the recommendations have been met, but you have wasted three months of both his and my time*" to anticipate potential misunderstands or interpretations of the quote.
- Development of familiarity with the culture in each setting was undertaken to ensure a high degree of compliance among the participating orthopedic department and municipalities. This was done before the first data collection dialogues took place. This was done via introduction and information meetings related to the project and how the project could fit best in current daily practice.

- Site triangulation (one orthopedic department and three rehabilitation centers in municipalities) was applied which reduced the potential effect of specific local factors influencing the study.
- Honesty among the study participants was ensured via the following actions: Participation in the interviews was anonymous and voluntary and the participants could withdraw their participation at any time, at the beginning of the interviews it was made clear that there was no right answers to the questions and that we were interested in their thoughts on the questions, finally it was emphasized that the participants could contribute thoughts and ideas without losing reputation or credibility among their managers.
- Continuous meetings were held in the research group to ensure a coherent process. For an example, discussion of the initial thoughts after interviews and discussion of initial codes and themes and interpretation of the underlying meaning in the data. At these meeting supervisors and senior researchers contributed with ideas and a proposed cause of action to include these in the research process. For an example, the preconceptions of the primary investigator and the potential effect of these on the data and analysis were challenged.
- Member check was applied during the interviews to check the data "on the spot". For an example the primary investigator would ask "so, what you are saying is" or "do I understand you correctly if what you mean is xx". This could then lead to an elaboration of the statement made. During the focus group interview with the physical therapist's member checks also included questions asking if the other participants agreed in a statement made by another participant.

Transferability:

- The findings of the study were compared to other research findings to validate the results. These previous research findings are presented in the discussion where they are used to evaluate the results of the present study.
- To ensure sufficient contextual information related to the study setting a paragraph specifically addressing this was added to the manuscript (Context: The QUADX-1 trial). This paragraph is provided to enable readers to transfer the results into their own clinical or other settings. Further, detailed information related to the context was added throughout the methods section were appropriate.
- The phenomenon under study was a model of coordinated non-surgical and surgical care for patients eligible for knee replacement. The non-surgical treatment was home-based exercise with *one* exercise. These aspects of the study are described in detail in the trial protocol which there is a link to in the manuscript. The protocol is published as open access and thus freely available to all (https://bit.ly/2Iq01bl).
- The study design and applied methods are described in the methods sections and the limitation of these are discussed in the limitation paragraph. This includes for example the number of organizations taking part in the study and where they are based, any

restrictions in the type of people who contributed data, the number of participants involved in the fieldwork, the data collection methods that were employed, the number and length of the data collection sessions and the time period over which the data was collected.

Dependability:

- A detailed description of the data collection and analysis processes within the study is provided in the methods section of the manuscript. This is supported by references where further information can be located. To further ensure that the research process is logical, traceable and clearly documented we have enclosed an audit trial.

Confirmability:

- Interviews were chosen as data collection method as this is a recognized method to explore participants views and thoughts on a phenomenon. In this case specifically the perceived barriers and facilitators related to the design and intervention of the QUADX-1 trail. The interviews were guided by semi-structured interview guides to ensure that the interviews captured information related to the topic under investigation. This format enabled the interviewer to guide or re-focus the interview if the content of the conversation drifted away from the topic under investigation.
- The data was analyzed using inductive thematic methodology. This analytical approach was chosen as there was no a priori hypothesis. The aim of the study was exploratory ("*The aim of this study was to identify perceived facilitators and barriers*") why we found an inductive approach more appropriate. If we had had a theory or framework, we intended to use for the analysis we could have chosen a more deductive approach.
- An excerpt of the analytical process with step-by-step decisions, analytical considerations, and how the final results were agreed upon within the research group is presented in the audit trail.

References

1. Nowell LS, Norris JM, White DE, Moules NJ. Thematic Analysis: Striving to Meet the Trustworthiness Criteria. International Journal of Qualitative Methods. 2017;16: 160940691773384. doi:10.1177/1609406917733847

2. Shenton AK. Strategies for ensuring trustworthiness in qualitative research projects. Education for Information. 2004;22: 63–75. doi:10.3233/EFI-2004-22201

3. Guba EG. Criteria for Assessing the Trustworthiness of Naturalistic Inquiries. Educational Communication and Technology. 1981;29: 75–91.

4. Krueger RA, Casey MA. Focus Groups. A Practical Guide For Applied Research. 3rd Edition. Sage Publications, Inc.; 2000.

5. Kitzinger J. Chapter 3. Focus groups. 3rd edition. Qualitative Research in Health Care. 3rd edition. Blackwell Publishing Ltd; 2006.

6. Halkier B. Focus groups as social enactments: integrating interaction and content in the analysis of focus group data. Qualitative Research. 2010;10: 71–89. doi:10.1177/1468794109348683

7. Crabtree BF, Miller WL. Depth Interviewing. Second Edition. Doing Qualitative Research. Second Edition. 1999.
