# Supplementary material for: Perceived facilitators and barriers among physical therapists and orthopedic surgeons to pre-operative home-based exercise with one exercise-only in patients eligible for knee replacement: A qualitative interview study nested in the QUADX-1 trial
Source: PLoS One. 2020 Oct 23;15(10):e0241175. doi: 10.1371/journal.pone.0241175 (PMC7584251; doi:10.1371/journal.pone.0241175)
Supplement: S6 File — (PDF) [file pone.0241175.s006.pdf]

Informeret samtykke til deltagelse i interview vedrørende projektet
Coordinated conservative and surgical care of patients with severe knee osteoarthritis: a pragmatic approach (The PREHAB-TKA project)

Erklæring fra interviewpersonen:

Jeg har fået skriftlig og mundtlig information, og jeg ved nok om formål, metode, fordele og ulemper til at sige ja til at deltage.

Jeg ved, at det er frivilligt at deltage, og at jeg altid kan trække mit samtykke tilbage.

Jeg giver samtykke til at deltage i forskningsprojektet og har fået en kopi af dette samtykkeark samt en kopi af den skriftlige information om projektet til eget brug.

Interviewpersonens navn: ____

Dato: ____________ Underskrift: ____________________________________

Erklæring fra den interviewansvarlige:

Jeg erklærer, at interviewpersonen har modtaget mundtlig og skriftlig information. Efter min overbevisning er der givet tilstrækkelig information til, at der kan træffes beslutning om deltagelse i forskningsprojektet.

Den interviewansvarliges navn: ______________________

Dato:___________ Underskrift:__________________

Projektidentifikation: Etisk Komité, Protokolnummer: H-16025136. Datatilsynet, Region Hovedstaden, ID-nummer: AHH.2016-072, I-Suite-nummer: 04980.
