# Supplementary material for: Perceived facilitators and barriers among physical therapists and orthopedic surgeons to pre-operative home-based exercise with one exercise-only in patients eligible for knee replacement: A qualitative interview study nested in the QUADX-1 trial
Source: PLoS One. 2020 Oct 23;15(10):e0241175. doi: 10.1371/journal.pone.0241175 (PMC7584251; doi:10.1371/journal.pone.0241175)
Supplement: S7 File — (PDF) [file pone.0241175.s007.pdf]

**Anonymous transcribed focus group interview with physiotherapists (in original language; Danish)**

RSH: Ja, så optager den. Det er også lige til jeres information, så bliver det jo optaget det her. Øh. Og hvis I så gerne vil have fat i det her materiale efterfølgende, så siger I selvfølgelig bare til, så kan I få en transskribering af hvad det er der snakket om i dag. Og, øh, det er jo sådan et interview her der starter inden selve projektet sådan for alvor er gået i gang, for at høre, øh, jeres, øh, jeres tanker omkring det på nuværende tidspunkt. Og der bliver sådan fire/fem overordnede emner, som er: 1) jeres rolle denne her type træningsintervention, 2) så er der det her med ikke-superviseret træning, 3) at det er en enkeltøvelses-tilgang til, øh, rehabilitering, øh 4) også det her med, at vi har det her teknologi inde over med BandCizeren, også til sidst, 5) æh, hvis det passer omkring den her tværsektorielle model med hospital/kommune kommunikation. Øhm, og øhh, nu skal jeg lige se engang. Det tager ca., nu har jeg sat to timer af, nu må vi se hvor lang tid det tager, og vi holder sådan ca. en pause hver halve time, og hvis I har brug for en pause på et eller andet tidspunkt, så holder vi en pause. Øh, og så vidt muligt hvis I øh kan sætte jeres mobiltelefoner på lydløs imens, så øh vil det være at foretrække. Det er sådan set lige introduktionen her. Er der nogle spørgsmål til det? Nej.

Så har jeg lavet den her model, som er fra erfaring da vi lavede et prøveinterview, det er bare for sådan at vise projektets flow, sådan så vi alle sammen er nogenlunde enige om det. Øh, så jeg prøver bare lige at skitsere hvad der er der foregår, det er at patienten jo bliver henvist herud til (hospitalet) af egen læge, også er det jo at de snakker med kirurger, også tester jeg dem, hvis de gerne vil være med. Så kommer du ud til jer, for instruktion og begynder at træne, og det er jo så et 12 ugers forløb, og undervejs i det forløb er det jo så at de kommer ind ved uge 4 og uge 8 til sådan opfølgning. Og i virkeligheden når I har set dem her i uge 8, så ser i dem ikke igen. Fordi når de så kommer ind igen efter de 12 uger, så kommer de så tilbage hertil (hospitalet), for at få en ny tid med kirurgen, for at snakke om hvad deres nuværende status er, også tester jeg dem selvfølgelig igen. Også er det her at der bliver taget en beslutning om hvorvidt de skal opereres. Og hvis de så bliver opereret så tester vi dem også efter operationen. Det er sådan set en del af projektet hvor I er færdige med at arbejde med dem. Øh, også bare for at gøre, hele ideen med projektet det er jo at skabe et bedre beslutningsgrundlag for hvorvidt kirurgi er den rigtige løsning ej. Ja, der er ikke noget i det her?

??: Nej.

RSH: Fremragende. Godt, øh i det her forum vi sidder i nu her, er det jo selvfølgelig jer seks jeg gerne vil have til at snakke om tingene, så jeg er mere sådan en moderater der stiller spørgsmål, også må I ellers gerne snakke til hinanden. I må selvfølgelig gerne spørge mig hvis der er noget der er uklart, men ellers er tanker at I seks skal snakke. Øh, og I siger bare alt hvad I har lyst til, jeg lærer noget af det hele. Ja, så inden vi lige går i gang med det, øh det første spørgsmål, så må I

gerne lige sige jeres navn, og øhm hvad hedder det, hvor længe i har været fysioterapeut og om I har noget, æh, supplerende uddannelse. Ja…

PHYSIOTHERAPIST 6: Uddannelse? Er det al slags uddannelse, eller er det bare inden for faget? Altså, æh…?

RSH: Al fysioterapirelevant uddannelse.

PHYSIOTHERAPIST 6: Godt.

RSH: Du må godt sige hvis du har noget andet, men det er mest det første jeg tænkte på. Ja… Vil du starte xx?

PHYSIOTHERAPIST 1: Ja, det vil jeg gerne. Jeg er øh, fysioterapeut i xx Kommune og æh, jeg har været uddannet siden xx. Så det er xx år efterhånden, men øh ja.

RSH: Har du noget supplerende uddannelse efter xx?

PHYSIOTHERAPIST 1: æh, jamen diverse kurser, men ikke sådan en master, diplom eller på den måde.

RSH: Okay.

PHYSIOTHERAPIST 6: Jeg hedder xx jeg er xx år. Øh, jeg blev uddannet i xx ´xx, øh så jeg har kun været fysioterapeut i knap xx år. Øhm, og jeg har også været på diverse kurser, men jeg har heller ikke nogle andre uddannelser end fysioterapi.

RSH: Okay.

PHYSIOTHERAPIST 5: Jeg hedder xx, jeg arbejder også i xx Kommune. Øhm, jeg har været uddannet siden xx x, så lidt over xxt år. Øhm, og det har også bare været lidt supplerende akupunktur, ikke noget ud over det.

Naturlig stille overgang til næste.

PHYSIOTHERAPIST 2: Og jeg hedder xx og jeg har været uddannet siden sommeren ´xx. Og jeg har også nogle kurser, som det eneste.

Naturlig stille overgang til næste.

PHYSIOTHERAPIST 3: Jeg hedder xx og jeg, æh, blev uddannet fysioterapeut i xx. Har ikke nogen anden uddannelse end supplerende kurser.

Naturlig stille overgang til næste.

PHYSIOTHERAPIST 4: Jeg hedder xx, jeg er udannet i ´xx, og har været her i xx Kommune og har haft nogle xx her i området og xx. Og øhm, specialkurser har jeg ikke haft så mange af, men jeg har akupunktur og ”gammeldags” kurser, som blev taget for xx år siden.

RSH: Ja, godt. Okay, jamen øh tak for det. Så lad os starte med det første her. Og det øh, har overskriftet: *Fysioterapeutens rolle i den her træningsintervention* vi laver som så er det her instruktion efterfulgt af ikke-superviseret træning derhjemme. Så det er lidt det det kredser om det her. Øh, så det jeg godt kunne tænke mig at vide lidt om er, hvilken rolle I føler I for i det her set-up. Og i skyder bare ind hvis I vil sige noget…

5-10 sek tænkepause.

PHYSIOTHERAPIST 2: Altså jeg har tænkt, det er jo, de kommer for at få instruktion i én eneste øvelse, og vi skal ikke tage os af alt det andet. Øhm, alle de spørgsmål de nu måtte have og, om alle mulige andre steder på kroppen de har ondt eller, hvad det nu end måtte være. Jeg har i hvert fald tænkt, nu har jeg jo kun set tre, men jeg havde lyst til at undersøge dem nærmere for et eller andet andet også, eller i relation til deres knæ-artrose. Ja, nu er det jo kun den her eneste øvelse de skal have.

RSH: Ja.

PHYSIOTHERAPIST 5: Ja, vi har jo ikke haft nogle inde i xx…

RSH: (afbryder), nej nej…

PHYSIOTHERAPIST 5: Er vi de eneste der ikke har haft det? I har heller ikke (henvendt til xx og xx)?

PHYSIOTHERAPIST 3: Nej, vi har heller ikke haft endnu.

PHYSIOTHERAPIST 5: Okay.

RSH: Det er ligeså meget, sådan jeres, selvom I ikke har haft nogle patienter endnu.

PHYSIOTHERAPIST 5: Ja ja,

RSH: Så hvad er jeres er jeres umiddelbare tanke omkring det, også inden det er startet.

PHYSIOTHERAPIST 5: Sådan meget superviserende, ikke så meget indgribende måske, men mere bare at holde øje med det.

RSH: Ja, kan du prøve at uddybe det lidt sådan…

PHYSIOTHERAPIST 5: Ja, jeg tænker vi skal jo som sådan ikke ind også lave noget manuelt eller noget. Ja, vi skal jo som sagt bare instruere i den her ene øvelse. Ja, øh, holde øje med den. Så det er vel bare at sørge for at det hele falder på plads, og give noget information. Selvfølgelig heller ikke for meget ift. projektet.

PHYSIOTHERAPIST 6: Umiddelbart så tænkte jeg om. Var det hvad borgeren tænkte? Eller sådan, nu er de selvfølgelig blevet instrueret af jer inden ikke, om de tænker at det er sådan relativt lidt de skal lave. Øh, ift. at det jo er et ret omfattende problem, ikke. Jeg ved ikke om de så'en tænker sådan, æh, det har jeg i hvert fald gået og tænkt over, ik. Hvordan at de vil opfatte det. Om de kan

stille sig tilfredse med det eller, med den ene ting. Sådan har jeg det i hvert fald, så skal man gerne give dem nogle ting ikke, de kan arbejde med. Sådan to, tre måske fire hvis de har gode ressourcer, ikke. Så de kan arbejde lidt øh. Så jeg er lidt spændt på at se om det bare er helt fint med dem, og det, om de godt kan forstå…

PHYSIOTHERAPIST 5: Ja, eller om de hellere ville have haft træningen (mere træning?)

PHYSIOTHERAPIST 6: Ja, eller sådan, men det er de jo allerede blevet sat i udsigt, ikke, altså inden de startede.

PHYSIOTHERAPIST 5. Ja, ja.

PHYSIOTHERAPIST 6: Men derfor kan folk godt nogle gange alligevel være lidt, øh, ???. Også have en anden holdning til det når de står ude på stedet, ikke. Så det er jeg lidt spændt på at se.

PHYSIOTHERAPIST 2: Det er selvfølgelig et lille grundlag stadig, men der er ikke nogle der har kommenteret det indtil videre. Som om de, altså de har jo været velinstruerede når de kommer herude fra xx (hospital), så de har været ret klar over hvad det er det handler om.

PHYSIOTHERAPIST 6: Mm, ok.

PHYSIOTHERAPIST 2: Der er heller ikke nogle der har nævnt noget om, øh, om der er andre der skal træne det samme, eller på en anden måde.

PHYSIOTHERAPIST 6: Ok.

PHYSIOTHERAPIST 5: Hvor lang tid har det sådan taget i instruktion?

PHYSIOTHERAPIST 2: Cirka, ehh, en halv time til tre kvarter lidt afhængigt af deres spørgsmål også er alt muligt uddybende snak.

PHYSIOTHERAPIST 3: For mit eget vedkommende så tror jeg, at det vil være en udfordring for mig, det her med at jeg ikke må tale over mig (griner).

RSH: Ift. hvad?

PHYSIOTHERAPIST 3: At der er nogle informationer jeg ikke må give. Altså den her, øh, begrænsning jeg selv har fået. Og os den lyst til at undersøge, og ting som man så skal holde sig fra. En uvant rolle som man lige skal øve sig lidt i. Også har jeg en forventning om at når de kommer, så er de vildt motiverede fordi det jo er os der sidder med den øvelse der skal lindre deres smerte. Og det er jo også ret spændende. Også har vi jo kun en, lige præcis ikke. Ret stor lille øvelse.

PHYSIOTHERAPIST 6: Ja, det er jo ofte sådan at der er måske noget der virker, og noget øh, så hvis man finder ud af, nå den virker ikke eller, så ved du godt at du har nogle andre (øvelser) du ved der virker, så kan det være at man kan bygge noget andet på i et øvelsesprogram.

PHYSIOTHERAPIST 3: Ja.

PHYSIOTHERAPIST 6: Altså det hele står og falder med den ene øvelse, ikke. Altså….

PHYSIOTHERAPIST 3: Ja, det er jo lidt uvant. Også tænker jeg også det her med, øh, det er nok mere et spørgsmål. Der er mange ude i xx Kommune, der bliver henvist til GLAD-træning, når de har ondt i knæene hos os. Om de er klar over det bevidst. Altså, er det et valg at de har valgt fra frem for, for øh, undersøgelsen her. Altså om de ved hvad de jo så ikke for, eller hvad man skal sige. Det har jeg tænkt lidt over. Om de er klar over det?

RSH: Ja…

PHYSIOTHERAPIST 3: Har de fået muligheden eller har de sådan bevidst ikke fået tilbudt GLAD-træning?

RSH: Njjaamen, øh, den kirurg der indtil videre har inkluderet, han er velvidende om hvad der foregår her (studiet), så dem han kan se der kan passe ind her, de bliver ikke tilbudt GLAD, øhm, hvis de ligeså godt kan være med her.

PHYSIOTHERAPIST 3: Ja. Hvad nu hvis de nævner ordet GLAD-træning når de kommer?

RSH: Så er det jo et alternativt behandlingskoncept eller behandlingsform. Bare lad være med sige om det er værre eller bedre.

PHYSIOTHERAPIST 3. Ja, ok.

RSH: Har I mere til det umiddelbare omkring jeres rolle ved denne her…

PHYSIOTHERAPIST 1: Jeg tænker at det bliver, altså det er en meget håndgribelig øvelse, som de skal hjem og lave, og den er meget nem at gå til. Og det tænker jeg, det må også være rart for patienten der kommer. Øh, at der ikke er så mange spørgsmål når de så er kommet hjem. Hvordan var det lige jeg skulle lave den her øvelse, og jeg har en pjece med hjem. Øh, og det er meget sådan håndgribeligt, hvor mange gange skal jeg lave den og hvor mange gange om ugen skal jeg lave den. Ikke, så kan de ligesom sige nu har jeg lavet mine hjemmeøvelser, som jeg skal. Så det synes jeg egentlig også er en force ved det.

RSH: Ja. Nu kom du selv lidt ind på det, altså vi kan godt tage den lidt over, så vi gør det her spørgsmål lidt mere positivt. Så hvad tænker I kan være fordelene ved denne her model? Jeg vil gerne høre hvad I tænker om det.

PHYSIOTHERAPIST 1: Netop, at det er simpelt og tilgængeligt. Det kræver ikke det store træningsudstyr eller at de skal fragtes sig fra hjemmet ned på et træningscenter. Øh, dem vi har set indtil videre de arbejder også, så se kan plotte det ind i dagen, når det lige passer.

RSH: Ja. Hvad med jer som fysioterapeuter, hvis I ikke skal tænke så meget på hvad patienten oplever, men hvordan I oplever det.

PHYSIOTHERAPIST 2: Jeg tænker det er interessant at se hvad det kan gøre med en øvelse alene.

PHYSIOTHERAPIST 4: Jamen, altså jeg har det på samme måde. Det er en meget simpel øvelse den her. Altså, så det bliver spændende og se hvad de for ud af det. Jeg glæder mig sådan mest til at komme i gang, for vi har heller ikke fået nogle endnu. Vi har bare afprøvet det på os selv, ikke, og gennemgået de her øvelser og set hvor meget vi skal strække den her elastik for at få noget max ud af det. Vi har fundet ud af at den kan komme meget langt…

PHYSIOTHERAPIST 3: Vi har fundet ud af at gøre elastikken kortere…

RSH: Okay, er der nogle flere der har noget til fordelene for jer?

PHYSIOTHERAPIST 3: Altså, jeg synes det er sjovt at være med i noget større. Øvelsen i sig selv er lidt småkedelig, men projektet, altså det at være med i en større ting er rart.

RSH: Ja, mmm. Så er der jo også den anden side, omkring om der skulle være nogle ulemper ved jeres rolle i for i det her. Hvad tænker I om det?

PHYSIOTHERAPIST 6: Altså, jeg vil jo nok sige, at man er jo låst i det man laver ikke, så øh, altså i det man normalt havde tænkt sig at lave med sine borgere. Men det vidste man jo godt, men det er det eneste jeg lige kan komme på. Altså, hvis man tænker på ens syn på det, man ved jo ikke hvad det giver med en øvelse.  Man tænker også lidt på hvad de måske kunne have fået, ikke. Det gør jeg da lidt, øh.

RSH: Kan du prøve at uddybe det lidt?

PHYSIOTHERAPIST 5: Ja, altså træningsudbytte. Men det er jo det der bliver spændende at se, ikke. Hvad det giver, ikke. Øh, men sådan vil det jo altid være, at man tænker sådan. Det har jeg da i hvert fald tænkt. Men det bliver spændende at se om de for lige så meget ud af det, og om man så behøver at sætte lige så hårdt ind som vi måske nogle gange gør, ikke.

PHYSIOTHERAPIST 1: Jeg tænker også at mange af dem, de kommer jo, og de har ondt, måske fordi de har en uhensigtsmæssig måde at dele deres aktiviteter og hvile op på. Øh, om jeg så må gå ind og undervise borgeren som jeg plejer at gøre med nogle pacing principper, eller er det helt skåret væk i det her? Så de bare mere kommer ind og nu skal de lære den her øvelse, også skal de hjem og lave den, færdig. Eller må jeg godt gå ind i en dialog omkring, hvad gør du så når du har ondt, eller hvor meget gør du så og…

RSH: Ja, er det et spørgsmål?

PHYSIOTHERAPIST 1: Ja, det er lidt et spørgsmål også ja.

RSH: Øh, jamen altså, man, så mange redskaber man kan give dem mens man er sammen med dem må man gerne give dem så synes jeg, inden for rimelighedens grænser. Pædagogiske redskaber osv.

PHYSIOTHERAPIST 1: Ja, for det er jo ikke noget der går ud over øvelsen eller den måde de laver øvelsen på.

RSH: Så hvis man kan give dem et redskab mere ud over selve øvelsen, men mere pædagogisk, sååå, det ligger sådan også lidt i det. Det er i virkeligheden det næste spørgsmål. Jeg fletter det lige sammen her.

RSH: Det er det her med at det er self-management. Altså at man ligesom giver patienterne noget uddannelse også skal de selv kunne håndtere det. Øh, så ja. Men spørgsmålet det går så på hvor I… det er anderledes fra hvad i plejer at gøre? Det her med at I måske giver lidt mere ansvar til patienten, også trækker jer derfra. Hvordan afviger det fra jeres normale praksis?

PHYSIOTHERAPIST 6: Altså, jeg kan godt lide det. At man giver noget ansvar over på, altså at de skal tageansvar for deres, altså det skal de også normalt ikke, men altså det kan jeg egentlig godt lide det princip og se om det holder i princippet ikke. Om man kan give over, hvor meget man kan trække sig. Det synes jeg er spændende, øh, og om det holder. Og se om de for gjort så meget som man har tænkt ikke, at de skal gøre. Det kan jeg godt lide, altså tanken om det.

PHYSIOTHERAPIST 3: Altså, jeg øh, jeg træner også folk i GLAD-konceptet, øh, som jo går rigtig meget ud på at de skal superviseres i deres træning. Øh, det ved jeg ikke rigtig hvad jeg synes om (superviseringen i GLAD), men jeg synes det er vildt interessant så at stille det modsatte (indeværende projekt) op overfor. Fordi GLAD-træning det fylder ret meget i vores kommune i øjeblikket. Jeg vil så også sige, at der altså ikke er lige så mange der for lavet nye knæ de sidste to år, efter at GLAD-træningen er startet, men der bliver også brugt rigtig mange ressourcer på det.

PHYSIOTHERAPIST 1: Er det ikke lige så mange, eller lige så mange?

PHYSIOTHERAPIST 3: Der er ikke lige så mange der for lavet nye knæer i øjeblikket hos os, efter at GLAD-træningen er gået i gang. Men der bliver brugt rigtig mange terapeutressourcer på det fordi konceptet jo skal overholdes, og det er så superviseret alt sammen. To gange ugentligt. Også er det jo interessant at stille det modsatte op ift. også se. Altså, på alle måder interessant.

PHYSIOTHERAPIST 2: Man kan sige vi prøver jo også i den almindelige forløb vi har at give dem ansvar ift. hjemmeøvelser osv. Her er det så bare den eneste del af hele interventionen. Så det der med at vi ikke kan følge op på hvordan det går det er anderledes.

PHYSIOTHERAPIST 5: Hvordan er det egentlig hvis der er en der begynder at få mere ondt af øvelsen, skal vi så sige til dem at de skal lave øvelsen mindre, eller skal de bare køre på indtil projektet er slut?

RSH: Ja, øh, de skal jo inden for rimelighedens grænser skal de blive ved. Så det er sådan beskrevet, at øh, hvis det er til at håndtere så mindsker de modstanden i elastikken, så de kan træne inden for hvad der er acceptabel smerte. Og hvis det så er al for meget så skal de stoppe. Også genoptage det når de kan. Og i yderste konsekvens så kontakter de mig, også finder jeg ud af om de kan være med eller ej. Så der er ligesom et par niveauer i det her.

PHYSIOTHERAPIST 5: Okay.

RSH: Øh, ja, er der sådan nogle umiddelbare, nu har i sådan været inde på fordele omkring det. Kan I se nogen ulempe ved at I sådan, at de træner derhjemme, og at I ikke ser dem så meget. Er der noget i tænker omkring det? Fordi at der var en del fordele I kom ind på her, men er der også noget I kan se der er negativt?

PHYSIOTHERAPIST 5: Det er selvfølgelig svært at se kvaliteten også. Jeg ved godt, registrerer den (BandCizer) om de kommer helt ud i end-range elastikken? Eller hvordan?

RSH: Nej, det gør den jo ikke.

PHYSIOTHERAPIST 5: Det er i hvert fald en ting der kunne mangle. Om man kan se om de for den lavet helt.

Nogle i baggrunden: Mmmm.

PHYSIOTHERAPIST 1: Ja, at der går fire uger inden vi eventuelt kan rette dem i den øvelse, som de sidder og laver. At de ikke laver den med nok modstand f.eks., eller at de ikke laver den, altså hvis de sidder og laver en fejlstilling mens de gør det. Eller et eller andet som der kunne gøres anderledes. Også går der fire uger inden vi har mulighed for at rette det.

PHYSIOTHERAPIST 2: Jeg tænker også det jo er en del af projektet, at vi skal se hvor godt det går med netop om de kan selv, af dem der nu engang bliver inkluderet. Med så lidt supervision og follow-ups. Ja, men det bliver jo interessant at se. Det kan godt være at de kan finde ud af at sidde og lave øvelsen korrekt og har forstået princippet i det, mens de sidder der. Men hvordan det så ser ud efter fire uger er jo lidt sjovt.

RSH: Ja, det bliver spændende, det gør det.

RSH: Ja, nu skal jeg lige se engang her… Føler I at hvis som fysioterapeut at I mister noget af det I måske er uddannet til ved denne her model? Hvis I forstår hvad jeg mener.

PHYSIOTHERAPIST 6: Ikke umiddelbart, taget i betragtning af at det jo er et afgrænset projekt og hvis, altså lige nu, altså både ja og nej. Altså sådan fordi det kan godt være at man sådan mister noget supervision og noget hands-on at man, vil gerne være på, man vil gerne være med i hvad der foregår og rette de ting ind, som hvis der er nogle fejl i træningen. Men samtidig så er der også positive ting i det der. Altså både ja og nej, altså.

PHYSIOTHERAPIST 5: Det er jo vigtigt at se om det virker, det er jo noget vi bruger meget på holdene i hvert fald, og under behandlingen. Det der med quadriceps styrke, det er meget vigtigt at få at vide om vi skal blive ved med at bruge det.

PHYSIOTHERAPIST 2: Hvis det var den eneste type forløb jeg så, så vil jeg nok synes at jeg manglede noget andet. Men i og med at det er i projektsammenhæng, så synes jeg ikke at jeg mangler, eller ikke føler mig fysioterapeut. Ikke endnu i hvert fald (haha).

PHYSIOTHERAPIST 1: Jeg kan godt have en lille bekymring på sigt ikke, med hvordan bliver de her resultater der så kommer, hvordan bliver de så tolket. Og hvilke konsekvenser for det så bagefter. Fordi der kører mere og mere med at øh, nu også med at vi kører IKURA (??) i xx Kommune, som er sådan et system hvor man træner med kropssensorer. Og det bliver meget sådan at, altså det bliver en *one size* til alle. Og folk er bare ikke ens. Det er ikke papkasser vi laver. Det er forskellige mennesker og det er forskellige behov. Øh, og det synes jeg ville være ærgerligt hvis det var den konsekvens som det gav.

PHYSIOTHERAPIST 2: Jeg tænker ikke at det nogensinde kommer til at stå helt alene. At det bliver sådan det typiske forløb at de skal komme ind til instruktion i denne her ene øvelse. Det kan være det er naivt tænkt, men øhm…

PHYSIOTHERAPIST 1: Jeg tænker at der bliver sparet alle steder, så hvis man kan se en muligheder der og der lige pludselig er noget der bliver tolket som om at det er et resultat man kan bruge på det, at så bliver det også *one-size* der. Men det er måske fordi jeg har været i faget så længe og bare set hvordan der er blevet skåret hele tiden. Øhm, ja så det tænker jeg, men det er sådan lidt bekymringer at man fremover ikke ser det hele menneske. Eller ser lige præcis hvordan fru Hansen er anderledes end hr. Jensen og kan sætte ind på det. Også selvfølgelig hvis det er et godt resultat her, så kan bruge det i behandlingen, helt klart.

RSH: Har i nogle andre, har I nogle kommentarer til det?

PHYSIOTHERAPIST 4: Nej, jeg ved mest umiddelbart at det jo er et projekt det her, ligesom alle projekter, det kører jo meget stringent sådan, øh, nu kører vi det sådan øh, og når man melder sig til det her projekt, ja så kører det sådan her. Så jeg har det sådan at nu er jeg spændt på at køre det på den her måde her. Og hvordan det så bliver brugt senere hen i videre forløb det må, for det første så må vi se hvordan det ender her, også er det jo rigtigt, altså kommunerne de skærer jo ned under alle omstændigheder ikke, så vi må se hvordan det kører det her.

RSH**: Okay, så springer vi lidt videre til en anden, det flyder lidt sammen det her. Det er en anden overskrift, øh, så det her det handler omkring det at lave træning før operation. Øh, om I ser, hvilke muligheder I ser ved at træne de her patienter før de eventuelt bliver opereret.**

PHYSIOTHERAPIST 5: Forhåbentligt undgå operation (hehe).

RSH: Ja.

PHYSIOTHERAPIST 3: Altså jeg synes allerede vi ser en, om man så vælger at træne på den ene eller den anden måde, og hvad for nogle metoder man vælger at gøre, så synes jeg at vi I xx Kommune allerede ser tydeligt ift. hvad vi for ind, at der er borgere i xx Kommune, der træner meget mere før operation end de gjorde før. Eller har positiv effekt.

PHYSIOTHERAPIST 1: Og også undgår operation som du snakkede om?

PHYSIOTHERAPIST 3: Jamen, vi for ikke så mange TKA'er de sidste to år. Er det ikke øhm, det synes jeg er tydeligt. Det er tydeligt at se.

PHYSIOTHERAPIST 6: Ude hos os der har vi også set eksempler på, at der er nogle der for en fornyet træningskultur, så ikke kun, også bare generel træning. Altså at de for glæden ved, og måske, at træne igen, altså under de rigtige omstændigheder ikke. Man for ligesom samlet nogle op. Også fordi de for reducering i smerter, eller nogle bliver helt smertefri. Og, øh, altså så man for samlet nogle op. Som måske kan holde sig selv i gang, øh, hvad enten det er i fitness center eller de går igennem arbejder, eller, ja der er også nogle der kommer ude i træningscenteret på nogle selvtræningshold og kommer lidt ind der, ikke. Så det er jo også en positiv ting som er tydelig at se.

PHYSIOTHERAPIST 5: Vi for også mange flere artrose knæ nu. Inden for det sidste halve år…

PHYSIOTHERAPIST 6: De spytter dem ud.

PHYSIOTHERAPIST 3: Ja, og færre TKA'er?

PHYSIOTHERAPIST 5: Ja, eller jeg ved ikke om der er kommet færre TKA'er, det kan vi ikke rigtig se endnu. Men, øh, mange mange flere med genoptræning inden i hvert fald.

PHYSIOTHERAPIST 6: Man kan også, altså det tager mange ressourcer. Altså det fylder, ikke.

PHYSIOTHERAPIST 5: Vi har aldrig nogen sinde haft et kun artrose hold, det har vi nu.

PHYSIOTHERAPIST 3: Ja, jamen jeg tror at vi har tre GLAD-hold og to artrose hold, eller et eller andet.

PHYSIOTHERAPIST 2: Dem I så ser der bliver opereret, for et nyt knæ eller noget. Er de så bedre rustet til at kunne varetage træning efterfølgende?

PHYSIOTHERAPIST 5: Hvis de har været der før og fået træningen hos os?

PHYSIOTHERAPIST 2: Ja.

PHYSIOTHERAPIST 5: Ja, det synes jeg helt klart. De plejer at komme sig hurtigt. Der har vi haft nogle stykker efterhånden faktisk, som vi har trænet inden. Det har både været individuelt eller på hold. Også kommer de på hold også bliver de opereret, og kommer igen bagefter.

PHYSIOTHERAPIST 2: Så har de et bedre udgangspunkt?

PHYSIOTHERAPIST 5: Ja.

PHYSIOTHERAPIST 6: Der er også mange der for øjnene op for hvad det egentlig kræver, ikke. Altså hvad det vil sige, også bare at skulle holde sig, også bare ved lige, men også efter operationen at de ved hvor meget det kræver bare inden operationen og blive klar til at blive opereret. Også bagefter, det er hvert fald det jeg har oplevet.

PHYSIOTHERAPIST 2: Mmmm.

RSH: Ja, det er jo mange positive ting set fra patientens side. Hvad med jer som fysioterapeuter, eller hvis vi skal se professionen. Kan I se nogle muligheder i det her eller fordele?

PHYSIOTHERAPIST 2: Ved det forebyggende træning?

RSH: Ja.

PHYSIOTHERAPIST 2: Jamen, jeg tænker da, der bliver som vi lige har talt om skåret ned på al mulig, og hvis der så bliver skåret ned på de her post-operative forløb så er der måske en større indsats vi kan gøre, øh, præ-operativt. Mmm, det lader til at der er rigeligt og gøre.

PHYSIOTHERAPIST 1: Jeg synes også. Altså, det er da altid en tilfredsstillelse at kunne hjælpe borgerne eller patienten videre uden at der skal skæres i dem. Altså, det øh, og de kan undgå de risici som ligger i det. Og det er jo ikke, altså, selvom man synes man for et nyt knæ, så er, øh, altså det er jo ikke ukompliceret for alle, og få et nyt knæ. Og det er ikke alle der synes de for et godt resultat ud af det. Så hvis man kan undgå det, så er det da helt klart positivt.

RSH: Mmmm, er der så nogle ulemper ved det? Hvis I skulle tænke på den anden vej rundt. Hvis vi tager jer som fysioterapeuter først ved det her.

PHYSIOTHERAPIST 6: Altså, indtil videre sådan som vi ser det, så er det jo altså en øget arbejdsmængde ift., altså afhængigt af om ressourcerne følger med. Fordi det er jo rigtig fint at man siger at man presser på med at få smidt dem ud i træning, og lige nu tror jeg vi havde 7-8 på venteliste med artrose.

PHYSIOTHERAPIST 5: Ja, også en del andre.

PHYSIOTHERAPIST 6: En del andre.

PHYSIOTHERAPIST 5: Lige nu er det kun os i teamet.

PHYSIOTHERAPIST 6: Ja, så der bliver fyldt godt på. Så, og man skal jo også varetage de andre opgaver vi har, ikke. Med hjemmetræning og des lige. Så det er lige som om, at hvis ressourcerne så ikke følger med i den øgede arbejdsbyrde der kommer så kan det godt blive svært. Altså også med kvaliteten ikke, af den intervention man så skal, øh, kunne give ikke. Øh, kan jeg godt se nogle problemer i hvert fald på sigt. Øh, så skal man gå ned på nogle andre områder ikke, eller være mere, altså…

RSH: Hvad mener du med at gå ned på nogle andre områder?

PHYSIOTHERAPIST 6: Jamen, altså, så bliver man nød til at prioritere i hvert fald. Så bliver de nødt til fra ledelsen at sige, hvis du skal bruge x antal timer på GLAD-hold og vi kun har så mange timer at give af, så bliver man jo nød til og finde på nogle nye løsninger i hvert fald. Altså, så må man jo tage den et sted fra jo. Det er jo det eneste alternativ vi har.

PHYSIOTHERAPIST 5: Eller vi ser nogle som måske skulle have været individuelle, og vi bare ikke har plads til dem, så må vi nogle gange starte dem ud på et hold, så der ikke går for lang tid. Også må vi tage dem individuelt bagefter, bare sådan at de ikke går helt i stå.

RSH: Nogle supplerende ting her?

PHYSIOTHERAPIST 3: Jeg er egentlig meget nysgerrig. Øhm, kører xx og xx artrose-hold eller kører I dissideret GLAD-koncept, eller?

PHYSIOTHERAPIST 5: Det er artrose-hold lige nu med udgangspunkt i GLAD.

PHYSIOTHERAPIST 3: Men I følger ikke den stringente…

PHYSIOTHERAPIST 5: Ikke som sådan.

PHYSIOTHERAPIST 3: …altså konceptet, og hvad der hører til ”pakken”…

PHYSIOTHERAPIST 5: Ikke som sådan nej. Vi kører en fællesopvarmning på en halv time, og individuelt en halv time efter, hvor vi også laver manuelt. Men den halve time fælles der, det er GLAD inspireret.

PHYSIOTHERAPIST 3: Inspireret. Og hvad med xx Kommune?

PHYSIOTHERAPIST 1: xx Kommune kører også GLAD. Øh, men de har valgt at samle det ét sted i byen. Så det er ikke alle, altså f.eks. der hvor xx og jeg er, der kører ikke noget GLAD. Øh, de bliver samlet på Nørrebro.

PHYSIOTHERAPIST 3: Okay, og på Nørrebro kører de det så rigtigt? Den stringente.

PHYSIOTHERAPIST 1: Ja, det går jeg ud fra at de gør.

PHYSIOTHERAPIST 2: Ja, det tror jeg også.

PHYSIOTHERAPIST 3. Ok, jeg spille bare lige høre.

PHYSIOTHERAPIST 2: Vi ser ind i mellem, men det er få, der bliver henvist pga. noget artrose.

PHYSIOTHERAPIST 3: Ja, okay.

PHYSIOTHERAPIST 5: Men det kan være vi kommer til at køre det. Altså jeg skal på uddannelsen (GLAD) her om et par måneder, så kan det være vi går mere over i det.

PHYSIOTHERAPIST 4: Vi arbejder jo direkte i GLAD-reden, så'n altså. Det er (kan ikke høre mere da andre bryder ind)

PHYSIOTHERAPIST 3: Men det er bare så interessant fordi, at det er så forskelligt ik også, med at det er mega superviseret. Og man kan nærmest, og nogle gange synes jeg det bliver lidt for meget, det her med, altså at de nærmest skal holdes i hånden.

PHYSIOTHERAPIST 5: Men jeg tror aldrig at vi kommer til at køre det 100 %, øh, ude i xx.

PHYSIOTHERAPIST 3: Også i forhold til det der (QUADX-1), det bliver spændende at se.

PHYSIOTHERAPIST 6: Hvad mener du med at holde i hånden, øh?

PHYSIOTHERAPIST 3: Jamen det er det her med, at de jo ikke laver noget træning hjemme overhovedet. De skal lave det superviseret.

PHYSIOTHERAPIST 6: Superviseret. Ja.

PHYSIOTHERAPIST 3: Og det skal være de øvelser, og, altså og der bliver lavet alle de her test før og efter, og. Og sådan noget ikke.

PHYSIOTHERAPIST 6: Ja.

PHYSIOTHERAPIST 3: Især ift. det du (RB) siger, at de kan selv.

PHYSIOTHERAPIST 6: Mmmm.

PHYSIOTHERAPIST 3: Og få noget… Altså, for jeg synes stadig at jeg oplever, at når de så slutter, nu er det ikke os der har det stringente GLAD, vi har det GLAD inspirerede, men når de så stopper, så er de sådan lidt, ”hvad nu”?

PHYSIOTHERAPIST 6: Okay, ja.

PHYSIOTHERAPIST 2: Men er der ikke også en del af GLAD-projekt, kan man ikke også træne hjemme? Men er det anbefalet og har, øh…

PHYSIOTHERAPIST 3: Ikke hvis du skal følge det stringent, så har vi også nogle artrose-hold hvor det bare er inspireret (af GLAD), ikke, øh.

PHYSIOTHERAPIST 2: Mmmm.

PHYSIOTHERAPIST 3: Det var dem hvor der ikke var plads på det rigtige GLAD-hold, de kommer så på det andet.

PHYSIOTHERAPIST 6: Hvordan tager de det?

PHYSIOTHERAPIST 3: Ehm, det er vidst noget, de har fået valget og vælge, vil du vente til der bliver plads på det rigtige GLAD-hold, eller vil du have en plads nu på det ikke så rigtige GLAD-hold, også kan du starte med det samme. Men på det der inspirerede hold, der træner de også lidt selv. Og de kan godt kun komme en gang om ugen også noget ikke, så.

PHYSIOTHERAPIST 6: Okay, fordi vi skal begge to på kursus her i det nye år ikke, altså i GLAD. Øh, og der er jo nogle, de komme jo mange med henvisning om, der står specifikt på gentræningsplanen GLAD, ikke. Så kan man sige ikke, jamen vi kan ikke tilbyde det lige nu. Vi

skal på kursus først men, vi laver det jo GLAD-inspireret ikke. Altså med hvad vi kan læse os frem til og… Så det bliver da spændende og se.

PHYSIOTHERAPIST 3: Ja.

PHYSIOTHERAPIST 2: Om der er noget der bliver anderledes af det i laver.

PHYSIOTHERAPIST 6: Ja. Det er jo os der styrer det, så det er jo der bestemmer hvordan det skal være.

RSH: Ja.

PHYSIOTHERAPIST 4: Nu er tilgangen til autoriteter jo også blevet mere og mere loose. Altså bare fordi det er fysser der siger, og man er en del af et eller andet GLAD-team, at man øh, at man ikke må gøre ditten og datten derhjemme. De fleste folk, de har det jo sådan, lidt mere i dag, altså jeg gør hvad jeg synes der er bedst for mig. Ikke sååå, æh. Så de gør jo, de laver det, som de synes at der er godt for at de skal komme videre i deres knæ-projekt.

RSH: Ja, jamen vi holder lige en lille pause så. Øh, det var første afsnit. Så, som sagt tag lidt mere af det der, og hvis der mangler noget så siger I til.

Der mangler kaffe…

**xx og jeg er gået for at snakke og lave mere kaffe**

PHYSIOTHERAPIST 2: Har I indtryk af, dem der bliver henvist til GLAD, er de lige så dårlige, som dem der for en operation? Er det samme sådan udsnit af borgere?

PHYSIOTHERAPIST 3: Det kan være begge dele. Altså der kommer også mange der bare har en lille menisk eller bruskskade ikke, som de ikke rigtigt har gidet og, øh, lavet en scanning.

PHYSIOTHERAPIST 1: Så det er ikke noget artrose, men det er noget, de har brug for træning, fordi det er jo en god træning…

PHYSIOTHERAPIST 3: Ja, eh, fordi hvis de ikke rigtigt gider og øh, bruger ressourcerne på at undersøge den her lille meniskskade…

PHYSIOTHERAPIST 2: Så nu er der et tilbud om træning, så nu er det nemt at…

PHYSIOTHERAPIST 3: Ja, eh, så prøv med lidt træning først, også kom tilbage, hvis det ikke er blevet bedre, ikke. Så for de en GLAD, ikke også.

PHYSIOTHERAPIST 1: De skal jo heller ikke konkurrere så meget mere, som de har skullet tidligere. (??)

PHYSIOTHERAPIST 1: Det jeg er lidt spændt på at se med dem der kommer du til os, ja igen med de få vi har set, så tænker jeg at dem der trods har fået en knæ-operation… (kan ikke høre pga. støj fra anden samtale)

PHYSIOTHERAPIST 3: Ja, i xx Kommune er GLAD gået hen og blevet sådan lidt en skraldespandsdiagnose…

PHYSIOTHERAPIST 2: Ja, så de egentlig er bedre dem vi ser, end dem der reelt for knæ.

PHYSIOTHERAPIST 3: Ja, altså der er nogle der kommer reelt med slidgigt i knæet, også er det så ift. hvor meget det er, om træning så hjælper. Også er der den der miskmask bombombe med menisk og bruskskader med folk over 50 der lige for en GLAD, for det er billigere end at lave en reel undersøgelse på hospitalet.

PHYSIOTHERAPIST 1: Ja, vi prøver lige. Men det er måske også bedre, tænker jeg, alligevel, fordi der er så meget nu der siger at man skal ikke opereres.

PHYSIOTHERAPIST 3: Ja ja, fair nok, men det er det der med at de kalder det GLAD, de kalder alt GLAD i øjeblikket, ikke.

PHYSIOTHERAPIST 1: Men det er så den træning man for, det er så bare ikke artrose de har.

PHYSIOTHERAPIST 3: Nej. Du for GLAD-træning, men du har en meniskskade.

PHYSIOTHERAPIST 4: Vi for nogle meget underlige genoptræningsplaner fra xx Hospital.

PHYSIOTHERAPIST 1: Ja ok, det er godt.

PHYSIOTHERAPIST 5: Vi skal passe på hvad vi siger, vi bliver optaget (reference til at optageren stadig er tændt)

Alle griner, ”nå ja”

PHYSIOTHERAPIST 5: Vi for også mange som gerne vil have GLAD, og som nærmest, altså ikke vil ud til os fordi vi ikke har det. Også siger vi jo bare at vi er meget inspirerede af det, altså, og det tager udgangspunkt i det. Så vil de jo gerne lige give det en chance ikke. Men der er mange som nærmest ikke vil ud til os, fordi vi ikke har nogle GLAD-terapeuter endnu.

PHYSIOTHERAPIST 4: Ja, for det er kæmpestort her i xx. Der er øh. Det er nærmest tre. Det er tre ikke fuldtidsstillinger, men de er øh, men de laver ikke ret meget andet de der tre.

PHYSIOTHERAPIST 6: Det fornemmer man godt, når man snakker med dem.

PHYSIOTHERAPIST 5: Ja. Når man snakker med dem ja.

PHYSIOTHERAPIST 3: Vi har fire terapeuter på det stringente rigtige GLAD-hold. Også har vi tre, to terapeuter på det der inspirerede hold.

PHYSIOTHERAPIST 6: Men hvordan er øh. Er terapeuterne så tilfredse med kun og øh, beskæftige sig med GLAD, eller er det ok, eller… Altså sådan…

PHYSIOTHERAPIST 1: Laver de ikke andet end GLAD?

PHYSIOTHERAPIST 4: Jo jo, de laver andet men øh…

PHYSIOTHERAPIST 1: Men de har mange hold bare med GLAD?

PHYSIOTHERAPIST 4: Ja. Der er mange folk.

PHYSIOTHERAPIST 3: De kører de der, de kører tre hold, er det tre hold? Nu.

PHYSIOTHERAPIST 4: Mmmm.

PHYSIOTHERAPIST 1: Er det en times varighed de er der, eller hvor meget er det?

PHYSIOTHERAPIST 3: Ja, en time to gange om ugen, også er der noget undervisning også.

PHYSIOTHERAPIST 1: Okay, ved siden af?

PHYSIOTHERAPIST 3: Ja ja, så underviser de også.

PHYSIOTHERAPIST 1: Ja. Men det synes jeg er skide godt… (kan ikke høre resten pga. støj)

PHYSIOTHERAPIST 3: Jeg tror det er to gange med undervisning ikke?

PHYSIOTHERAPIST 4: Jo.

PHYSIOTHERAPIST 3: Også start og slut test.

PHYSIOTHERAPIST 1: Ja.

PHYSIOTHERAPIST 4: Og de terapeuter der har med det at gøre, de er superglade for det.

PHYSIOTHERAPIST 1: De er også glade?

PHYSIOTHERAPIST 4: De er også glade. Ja, ikke. Der er mange der gerne vil være med i det her projekt (GLAD) og øh.

PHYSIOTHERAPIST 3: Men det er meget superviseret ikke.

PHYSIOTHERAPIST 4: Ja.

PHYSIOTHERAPIST 3: Jeg kan godt nogle tænke på de der GLAD-inspirerede hold, om man har lagt ressourcerne det helt rigtige sted (på GLAD??), nogle gange ikke.

PHYSIOTHERAPIST 4: Ja.

PHYSIOTHERAPIST 5: Hvis nu der er en der siger at vedkommende har ondt et eller andet sted. Må I så godt gå ind og lave manuelt eller et eller andet, hvis I ser det er relevant? Eller skal I være sådan fuldstændigt GLAD?

PHYSIOTHERAPIST 3: Altså, på det rigtige GLAD-hold må man ikke.

PHYSIOTHERAPIST 5: Det må man ikke nej. Okay.

PHYSIOTHERAPIST 3: Fordi dem der sidder og har de der koncepter tjener penge på det. De vil jo have nogle helt specifikke…

PHYSIOTHERAPIST 5: Nå de skal også have nogle data på det?

PHYSIOTHERAPIST 3: Ja, de skal have nogle data, ikke også. Og der må man ikke lave alt muligt andet. Så kan de ikke bruge det til noget.

PHYSIOTHERAPIST 5: Nej okay.

PHYSIOTHERAPIST 3: Det er sådan lidt forskningsprojekt–agtigt ikke.

PHYSIOTHERAPIST 5: Ja…

PHYSIOTHERAPIST 6: For I ikke mange diagnoser, eller sådan folk der skal have GLAD-træning hvor der viser sig, det sagde du godt nok at det var en skraldespands-diagnose, altså men.

PHYSIOTHERAPIST 3: Det er det blevet lidt i kommunen her, ja.

PHYSIOTHERAPIST 6: Hvor, øhm, det er muskulære problemer næsten. Altså vi har da en del, hvor når man så undersøger dem lidt nærmere så finder man ud af…

PHYSIOTHERAPIST 5: Så er det bare musklerne der gør ondt. Selvom de måske har fået diagnosticeret noget, så laver vi bare manuel, så er det bare væk

PHYSIOTHERAPIST 3: Altså, vi fik lavet de her fibromyalgi-hold ud af nogle af de her artrose borgere. Et lille samtræningshold. Jeg tror også fibromyalgi er sat over på et hold ikke.

PHYSIOTHERAPIST 5: Ja, ok. Hold da op. Fungerede det fint nok?

PHYSIOTHERAPIST 3: Ja, for de var jo ikke i det rigtige GLAD-koncept. Der sorterer de jo folk fra. Altså, du skal opfylde nogle kriterier for at kunne få lov, at få det rigtige GLAD-træning ikke.

PHYSIOTHERAPIST 5: Mmmmm. Okay, spændende.

PHYSIOTHERAPIST 2: Men det var næsten også for godt til at være sandt. Altså, jeg tror heller ikke, at man bare kan sætte alle ind i det der koncept. Der kan jeg bedre lide ideen om, at der også er mulighed for at sætte ind med noget manuelt, hvis det er det man vurderer der er brug for. Jeg nægter at tro det er alle der kan passe ind i ”GLAD-kassen”.

PHYSIOTHERAPIST 3: De sorterer nogle fra, ikke også…

PHYSIOTHERAPIST 6: Hvis, ja men, når man tager på det der GLAD-kursus, og man så kommer tilbage. Hva, er det så, man kan vel vælge at bruge det som man har lyst til? Er det ikke korrekt forstået?

PHYSIOTHERAPIST 2: Det kan man vel ikke, hvis du lover borgerne GLAD-konceptet. Så kan man vel ikke afvige.

PHYSIOTHERAPIST 6: Ja, nej…

PHYSIOTHERAPIST 5: Ellers, kan vi vel kalde det GLAD-inspireret stadigvæk?

PHYSIOTHERAPIST 3: Ja, okay.

PHYSIOTHERAPIST 2: Det ville jeg bedre kunne lide at gøre.

PHYSIOTHERAPIST 5: Fordi jeg ved ikke om vi kommer til at lave helt rene GLAD-hold.

PHYSIOTHERAPIST 6: Nej, det...

PHYSIOTHERAPIST 5: Det er noget vi selv bestemmer. Jeg kan meget godt lide den der del af det hvor vi tager den til side og laver manuel.

PHYSIOTHERAPIST 6: Det må vi jo tage en snak om.

PHYSIOTHERAPIST 5: Det kan da også godt være at vi laver et hold hvor det kun bliver GLAD.

PHYSIOTHERAPIST 6: Bare for at se hvad der virker bedst.

PHYSIOTHERAPIST 5: Ja.

PHYSIOTHERAPIST 2: Ja, de kommer jo også med forskellig baggrund. Nu er det her dissideret artrose, nogle har lidt brusk, øhm, fidelihut eller gammel menisk et eller andet.

PHYSIOTHERAPIST 3: Ja, ja.

PHYSIOTHERAPIST 2: Øhm, og de har jo ikke nødvendigvis brug for den samme som fru Jensen med…

PHYSIOTHERAPIST 4: Men kommunerne vil jo helst putte folk i kasser. Det er jo nemmere for dem (kan ikke høre slutningen af sætningen)

PHYSIOTHERAPIST 6: Faktisk at have en diagnose ja.

PHYSIOTHERAPIST 4: Ja.

PHYSIOTHERAPIST 5: Ja, helt sikkert.

PHYSIOTHERAPIST 2: Der kan jo være masser af ting, uden at jeg er helt inde i hvad det handler om, GLAD-projektet. Masser af øh, brugbare øvelser.

PHYSIOTHERAPIST 5: Ja, det tænker jeg også. Om der er noget nyt i det, ift. det man ligesom har lært tidligere. Det er jeg lidt spændt på.

PHYSIOTHERAPIST 2: I princippet kan man vidst lave alle øvelserne om.

PHYSIOTHERAPIST 5: Ja.

PHYSIOTHERAPIST 2: Jeg tænker også nogle gange om den, om GLAD-projektet har haft den effekt som det har. Fordi det/de netop, nogle gange, kommer til noget superviseret, de for noget information om, eller ift. vægttab og, og al det der. Om det mere er det egentlig, der har den gode effekt. At det dissideret er de øvelsers effekt på leddet, der tror mere at det…

PHYSIOTHERAPIST 1: Jeg tror det er kombinationen. At man for mere med.

PHYSIOTHERAPIST 5: Mmmm.

PHYSIOTHERAPIST 2: Jeg synes det er interessant med det forebyggende, men, men igen det skal ikke være så konceptlåst. Hvis det kan undgås.

PHYSIOTHERAPIST 1: Ja, altså det er jo det der både er force, og ikke force eller sådan noget.

PHYSIOTHERAPIST 2: Ja.

PHYSIOTHERAPIST 5: Så…

PHYSIOTHERAPIST 2: Så er der mere kaffe.

**Der hældes kaffe op og skramles med ting.**

RSH: Ja, øh, er I klar til at starte lidt op igen?

Flere: Ja.

RSH: **Ja, jamen inden vi går videre, så vil jeg gerne bare lige følge lidt op på, øh, lidt af det I har været inde på. Fordi bl.a. xx, du var inde på, øh, det har med GLAD kontra det som vi kører her med PREHAB, eller hvad vi skal kalde projektet. Øh, sådan graden af supervision. Og du var lidt inde på at du synes måske at i GLAD at der er for meget supervision næsten. Sådan som jeg hørte det.**

PHYSIOTHERAPIST 3: Ja, eller sådan i perioder af forløbet, ikke, altså.

RSH: Ja, og hvordan har du det med det?

PHYSIOTHERAPIST 3: Nå, men det er også fordi at jeg synes det er fedt og, øh, give noget ansvar over på patienterne ikke også.

RSH: Mmmmm.

PHYSIOTHERAPIST 3: Og synes også i stor omfang at hjemmetræning er vigtig. Især når man har en lidelse som er, øh, kronisk. Ikke, altså.

RSH: Mmmm.

PHYSIOTHERAPIST 3: Også er det bare også nogle gange det her med, at når folk skal tjene penge på nogle koncepter, så er det meget fornuftigt nogle gange at stille nogle kritiske spørgsmål.

RSH: Ja. Hvordan har I andre det med det her med at det er, øh, måske det koncept der er mest udbredt lige nu er meget superviseret? Også kontra denne her model?

PHYSIOTHERAPIST 2: Vi talte lige lidt om det her i pausen. Hvor jeg tænker det (alle griner lidt, da de netop har snakket om dette i pausen), at det lader jo til, at det har rimelig god effekt det GLAD. Men jeg tænker bare at det er umuligt at man kan proppe så mange ned i den samme kasse, til at det kan være effektfuldt. Om det så er selve træningen eller det at det er superviseret, og, fokus på vægttab og andre parametre der, i virkeligheden gør at det viser sig at være brugbart. Også tænker jeg at noget af vores vigtige rolle er også med de midlertidige forløb vi har, det er netop og kunne, eller og ja, hjælpe dem til at kunne blive motiveret til at kunne forsætte med noget træning. Og der lyder det lidt som om de er taget ret meget i hånden, som du (xx) lige beskrev der.

PHYSIOTHERAPIST 3: Det som jeg oplever…

PHYSIOTHERAPIST 2: At det med at de så fortsætter på egen hånd, at det lige pludselig det er lidt sværere.

PHYSIOTHERAPIST 3: Så er der selvfølgelig nogle undervisningsseancer og sådan noget, ikke øhm.

PHYSIOTHERAPIST 2: Mmmm.

RSH: Hvordan har I det med det med at de, øh, at de ikke måske, det lyder på mig som om at de, øh, nu må I korrigere, hvis de ikke er rustet når de så forlader jer? Er det lidt det jeg hører? Og hvordan har I det så med det?

PHYSIOTHERAPIST 3: Altså, de for noget undervisning i smertehåndtering og fysisk aktivitet og sådan noget. Øhm, med folk der søger sådan et koncept, så er det jo fordi de gerne vil have et træningstilbud og komme til. Ligesom og gå i et motionscenter. Og når de så er færdige, så synes jeg at jeg kan fornemme på xx (leder?), at så vil de gerne have et nyt sted og gå hen, også kommer de nede i xx motionscenter. Det er et motionscenter som ikke er kommunalt eget.

PHYSIOTHERAPIST 4: Ja, som de også skærer ned på lige pt her.

PHYSIOTHERAPIST 3: Ja. Så jeg ser dem egentlig bare vandre fra det ene træningstilbud over tli det andet. Det er jo også okay, så længe at man…

PHYSIOTHERAPIST 2: Hvis de fortsætter træningen i en eller anden forstand…

PHYSIOTHERAPIST 3: Det gør de. Også skal de ud og betale sig til et nyt. De har stadig væk i en eller anden grad lagt det over til nogle andre der fortæller dem ”hvad skal jeg lave”.

PHYSIOTHERAPIST 3: Mmmm.

PHYSIOTHERAPIST 5: Må man godt ligge et program til dem tilpasset træningscenteret, men som ikke nødvendigvis er alle GLAD-øvelserne, sådan til sidst?

PHYSIOTHERAPIST 3: Når de er færdige med GLAD, så skal de ud og finde noget andet selv, hvor altså…

PHYSIOTHERAPIST 5: Ok.

PHYSIOTHERAPIST 4: I xx kommune der har vi det vi kalder udslusningsholdet. Og det udslusningshold havde vi før i tiden på vores almindelige træningscenter, og det er så flyttet på xx stadion. Øh, og den henvisende terapeut laver et træningsprogram med de maskiner der er på træningscenteret. Det har ikke rigtigt noget med GLAD-øvelserne at gøre, altså.

PHYSIOTHERAPIST 5: Ok.

PHYSIOTHERAPIST 4: Det går kun efter de maskiner og de ting de nu kan lave dernede.

PHYSIOTHERAPIST 5: Ok.

PHYSIOTHERAPIST 3: Men folk der ønsker det, den træning, det er jo folk der ønsker at der kommer nogle og fortæller dem, hvad de skal gøre. Også når de er færdige så går de ud et andet sted, hvor der er nogle andre mennesker der kan fortælle dem hvad de skal gøre. Om de så skal betale for det, eller få det gratis, det er lidt lige meget. Altså, øhm, jeg kan være lidt i tvivl om de kan forbinde det de for i undervisningen med deres egen træning. Altså, man kender det fra sig selv, man for noget teori, også for man koblet den teori på sin hverdag. Altså, vi kender det når vi går på kurser også, ikke altså. Der er ligesom et lille hop fra det ene til det andet.

RHS: Er det noget i alle sammen kan genkende?

PHYSIOTHERAPIST 5: Jeg vil sige at vi plejer altid at sende dem videre til øh, et træningscenter sådan via Vestbadet eller Fitness World eller sådan noget. Også har de nogle maskiner som ligner lidt dem vi har, også plejer vi at køre dem ud, ved at introducere til dem. Også laver vi så et program hvor, at vi skriver de maskiner på ved navn i det center de så skal hen.

RSH: Ja.

PHYSIOTHERAPIST 5: For på en eller anden måde at lave sådan en glidende overgang. Det stræber vi imod i hvert fald. Det kan jeg meget godt lide. Sådan at man har lidt fingeren på pulsen, og sørger for at de bliver lidt selvhjulpne på en eller anden måde når de tager videre.

RSH: Ja.

PHYSIOTHERAPIST 1: Jeg synes da at en af mine fineste opgaver sammen med borgeren, det er og gøre dem selvhjulpne til at de kan klare sig uden mig, når de er færdige. Og de har fundet et tilbud, eller jeg hjælper dem med at finde et tilbud som de er motiveret for. Om det så er at træne videre selv, eller at komme i en eller anden klub, eller i et fitness center, eller whatever. Altså bare

et eller andet der motiverer dem til og fortsætte med noget motion og bevægelse. Det synes jeg er en af de allervigtigste opgaver. Det er noget vi har fokus på lige fra de kommer ind ad døren.

PHYSIOTHERAPIST 5: Ja.

PHYSIOTHERAPIST 2: Fordi vi fortæller dem samtidig med det, at vi prøver allerede at få fokus på det fra starten. Så fortæller vi dem jo også samtidig med det, at de er jo ikke færdigtrænet for sådan et midlertidigt forløb i kommunen. Så det er nødvendigt at de skal træne videre for at få fuldt udbytte af det.

RSH: Ja.

PHYSIOTHERAPIST 1: Men de er bare færdige med deres genoptræning, men træningen foresætter.

PHYSIOTHERAPIST 2: Ja. Det er sådan alle diagnosegrupper vi ser, der forsat har et behov.

RSH: Mmmm. Ja.

PHYSIOTHERAPIST 4: Jeg tror, at vi har halvdelen af de folk der er startet på udslusningshold, de for, på udslusningshold har de en max i måneden.  Altså enten en gang om ugen, eller to gange om ugen. Sååå, og når de er færdige med det lille en-måneds forløb så ser vi faktisk at 50% de forsætter oppe i motionscenteret. Og træner selv.

PHYSIOTHERAPIST 1: Det er mange.

PHYSIOTHERAPIST 6: Det er det faktisk.

PHYSIOTHERAPIST 2: Siger du at det udslusningshold det foregår i det andet træningscenter?

PHYSIOTHERAPIST 4: Ja, det foregår på stadion.

PHYSIOTHERAPIST 5: Det er da smart.

PHYSIOTHERAPIST 1: Det er en virkelig god ide.

PHYSIOTHERAPIST 4: Ja, altså jeg har udslusningsholdet, så jeg er der så de der to gange om ugen. Hvor de så, mange af dem kender mig mere eller mindre, sådan fra træningssalen fra xx hvor vi har, og øh, ja så viser jeg dem de maskiner der er der ikke. Eller introducerer dem til maskinerne, også fortsætter de så deroppe. Fordi de føler sig trykke ved maskinerne, og øh, for det meste bliver de så også og kommer i de tidspunkter hvor vi i forvejen er der ikke. Så kan de stadigvæk lige komme hen og prikke mig på skulderen.

PHYSIOTHERAPIST 1: Mmmm, god ide.

PHYSIOTHERAPIST 3: Men det er jo en indsats fra vores side, kommunens side at vi gerne vil lave den overgang fra GLAD-konceptet og over til, hvor de tager noget, hvor de kommer videre.

Men konceptet i sig selv det synes jeg ikke ligesom sørger for at de for ansvar, eget ansvar for noget.

PHYSIOTHERAPIST 5: Er det noget i selv har taget initiativ til, eller hvordan?

PHYSIOTHERAPIST 3: Ja udslusningsholdet, ja. Det er vores egen, øhm…

PHYSIOTHERAPIST 5: Ja, ok.

PHYSIOTHERAPIST 3: Fordi vi oplever at når de er færdige på holdet så siger de ”hvad”.

Folk snakker lidt i munden på hinanden (kan ikke høre det)

PHYSIOTHERAPIST 2: Er det kun for GLAD?

PHYSIOTHERAPIST 4: Det gælder alle, altså, alle kan komme på udslusningsholdet. Det er lige meget om de har, om det er skulderfolk, eller om det er hofte, eller knæ, eller ankel, eller det er øh, det har ikke noget direkte med GLAD og gøre.

RSH: Men hvordan har I det med at patienterne de siger ”hvad nu”? Hvordan for det jer til at føle?

PHYSIOTHERAPIST 3: Altså, hvis vi ikke havde noget andet at tilbyde dem, så ville vi jo nok blive lidt frustreret. Nu har vi jo vores udslusningshold, så, øh…

RSH: Mmmm. Hvordan er det så i xx og xx med det? Hvad tænker i om den model?

PHYSIOTHERAPIST 5: Vi har noget selvtræningshold bl.a. hvor de kan komme efterfølgende hvor der er en terapeut der står for det. Det er så dig (RB) der står for et af holdene i vores, som er der en gang om måneden eller kommer når han nu lige har tid og retter lidt til ikke. Og øh, de kan jo fortsætte der.

PHYSIOTHERAPIST 6: Ja, det er meget de ældre borgere som ikke kan se sig selv på nogen måde i et fitness center, ikke, som har. De kan simpelthen ikke identificere sig med at komme sådan et sted, ikke altså. Øhm, så det er dem vi prøver at prioriterer der kommer der ikke. Også prøver vi så vidt muligt og øh, opfordre de lidt yngre borgere, altså måske minus under de 65 til og at, kunne det ikke være noget og komme i xx eller, hvor de også har træningsfaciliteter, ikke også.

PHYSIOTHERAPIST 5: Som regel så laver vi også et program til dem, som de også kan følge.

PHYSIOTHERAPIST 6: Ja, eller i xx, eller hvis de ikke kan. Hvis man ser at det er en type, hvis man fornemmer at det er en der godt vil kunne forlig sig med at komme der ikke. Øhm, så prøver vi at opfordrer dem til at komme der. Også det der med mentalt og forberede dem på, måske, noget tid inden at de stopper, nå men på et tidspunkt så stopper du her, og hvad tænker du så der skal ske? Og synes du, at du kan varetage din træning selv, og er du usikker på noget, skal vi lave et program eller? Øhm, så vi prøver at gøre det. Også er der mange, de siger ja, også ved man jo godt hvordan det er ikke. Så har de svært ved at sætte sig op til det, de har ikke ressourcer til det. Men det vil altid være et problem. Men det er det der er interessant. Jeg tænker med GLAD med fastholdelsen

bagefter. Også hvis man så træner i et koncept som er svært at holde fast i bagefter tænker jeg, men det må vi jo se. Jeg har heller ikke været på kurset endnu.

PHYSIOTHERAPIST 1: Jeg tænker, hvis der kommer en borger, der siger til mig, ”hvad nu?”. Så vil de også gerne have mig til at finde løsningen.

RSH: Ja.

PHYSIOTHERAPIST 1: Ikke, altså det kunne jeg godt tænke mig at sende tilbage igen. Ja, hvad tænker du, at du skal nu? Fordi det er også lidt det der med, at hvis det kun er mig der står med svaret altid, så bliver de ikke så selvhjulpne.

RSH: Nej…

PHYSIOTHERAPIST 1: Jeg vil selvfølgelig gerne hjælpe dem på vej, men det er også lidt det her med at motivere dem til selv og tage ansvar. Det som at vi snakkede om før.

RSH: Mmhmm.

PHYSIOTHERAPIST 1: I stedet for at det er mig, der skal finde på hvad de skal.

PHYSIOTHERAPIST 1: Men vi har, altså spørgsmålet gik også på hvad vi gør eller…??

RSH: Ja, det er lidt denne her med hvordan I havde det med, det startede helt tilbage til hvordan I havde det med at slippe nogle patienter, som måske kun har fået superviseret træning, også når de forlader GLAD eksempelvis, måske ikke har de fornødne redskaber, hvordan I havde det med det?

PHYSIOTHERAPIST 1: Vi kører jo så ikke GLAD.

PHYSIOTHERAPIST 6: Nej, det gør vi jo så heller ikke. Og det er jo også, det er måske også det, måske hvis vi tager udgangspunkt i at det skal være GLAD fordi det kører vi jo ikke, vi kan jo kun forholde os til det vi gør nu ikke. Hvor vi måske, hvor der er nogle der, heldigvis er der mange der begynder at blive meget selvkørende, i noget, altså hvor du faktisk, altså du er jo kun superviserer, også er du faktisk ved dem, og du kan se at de kører det som de skal, ikke. Altså, man kan godt mærke at man begynder at få mere og mere hands-off-. Og de mere og mere komfortable med øvelserne og hvor det til sidst giver meget god mening og slippe dem, men når man så går over til GLAD-konceptet, og man er på hele tiden, det bliver lidt interessant hvordan det bliver.

RSH: Hvis nu man havde GLAD på den ene yderpol, også det her projekt på den anden yderpol, ift. mængden af supervisering. Hvor ser I så at man helst umiddelbart skal ligge?

PHYSIOTHERAPIST 6: Til og starte med, eller bare igennem…?

RSH: Lidt mere generelt.

PHYSIOTHERAPIST 5: Det kommer jo lidt an på borgeren. Der er nogle der har lidt mere behov for supervision.

PHYSIOTHERAPIST 6: Ja, det kommer an på deres ressourcer, og deres træningskultur inden. Der er nogle der kommer, som har været vant til at træne før i tiden, som er gået i stå og som godt ved noget, og de skal bare have et spark, og øh så starter man med at være meget på ikke, så stille og roligt så giver man dem mere fri ikke. Også til sidst så er det jo bare fedt, hvis man så… Jeg har ikke noget problem med at man går helt over i den løsning at man lader dem, næsten, klarer det hele selv. Hvis de kan klare det, altså hvis de gør det rigtigt.

PHYSIOTHERAPIST 1: Ja, så meget supervision i starten, eller i hvert fald mere supervision i starten også når man ved de kan med god kvalitet, så kan man slippe det mere.

RSH: Det er mere sådan generelt..?

PHYSIOTHERAPIST 4: Ja, det er jo meget individuelt at borgerne, ikke… Der er jo nogle der bare ikke gider gå i træningscenter, der er nogle der hellere vil sidde på en stol derhjemme og lave denne her øvelse. Og det kan jeg sagtens se for mig at der er… Hvis det virker det, jamen så vil de hellere sidde derhjemme og lave det her, end at man vil komme på et træningscenter eller gå til… Nu vil xx kommune, vi er ved og udvikle udslusningen lidt mere til øh, at tage kontakt til privatklinikker og til øh, så folk de starter på noget line-dans eller et eller andet jeg ved ikke. Nu kommer der en masse ting på bordet, som ikke øh, det er først lige startet. Og jeg er ikke line-dans terapeut (haha). Men vi prøver med flere og flere ting, så vi kan få skubbet folk ud. Og jeg synes da, vi har mange som hellere vil træne for sig selv derhjemme.

RSH: Det der med at de bliver skubbet mere ud. Synes du det er en god ide?

PHYSIOTHERAPIST 4: Ja, jeg synes da træning skal tilpasses til de folk der kommer. I stedet for og øh, sige du skal gøre sådan og sådan, når så halvdelen de er øh, hvor tingene de stritter på dem ikke. At faktisk ikke rigtig har lyst til det vel. Så de skal da, der skal da være nogle valgmuligheder.

RSH: Er det noget i alle sammen, en ide der er bred enighed om?

PHYSIOTHERAPIST 5: Ja, helt sikkert. (generelt bred enighed omkring bordet)

PHYSIOTHERAPIST 2: Kæmpe forskel. Lige xx og xx, der er et vælg af tilbud, af alle mulige forskellige aktiviteter, så det er nogle gange også kunsten i at kunne sortere lidt i det. Altså borgerne har på ingen måde kendskab til alt hvad der foregår, og det er nærmest også umuligt for os og have en finger på pulsen med alle tilbud. Så det er lidt om undervejs også og sortere og finde frem til hvad der kunne være det rigtige. Nogle gange er det bare hjemmeøvelser, hvis der skal laves noget. Og nogle gange er det noget helt andet.

PHYSIOTHERAPIST 1: Vores store udfordring ligger jo i, altså i de borgere, de geriatriske borgere som ikke selv kan transportere sig. Altså, som har brug for kørslen, og som for kørslen når de går til genoptræning, men så når de slutter der, så er der ikke noget tilbud mere om kørsel. Og det har været en udfordring altid.

RSH: Ja. Hvordan, og øh…

PHYSIOTHERAPIST 1: Også, deres tilbud det er måske at komme i et aktivitetscenter, hvor der er lidt træning tilknyttet, men så skal de være der hele dagen. Men nogle af dem har jo bare lyst til at komme og få træningen. Så kan man selv transportere sig, så er der faktisk mange muligheder for at gå til noget.

PHYSIOTHERAPIST 2: Ja, det er rigtigt.

RSH: Øh, der var en kommentar før pausen SH, hvor du nævnte det her med at patienterne de så at sige gør hvad de vil. Øh, uanset hvad/hvilken koncept eller kasse du prøver at putte dem ned i. Eksemplet var GLAD, hvor de fik at vide at de må ikke træne derhjemme, også sagde du at det gør de jo, som de vil. Hvordan har du det med det, at det sådan…

PHYSIOTHERAPIST 4: Som udgangspunkt har jeg det sådan, altså folk de har jo, det er jo øh, de er jo. Folk der tager ansvar, der selv tager ansvar for deres liv, det synes jeg da er en positiv ting. Så hvis de har lyst til at lave øvelsen 50 gange, eller sådan eller hvordan de nu synes det skal gøres, og de kan se at der en øh, at der sker noget positivt, at de for mindre smerter, synes jeg da at det er super fint. Folk tænker jo selv. Man er jo ikke så autoritetstro mere som man var, altså var for 30 år siden eller for 20 år siden, ikke. Der er jo mange ældre borgere som er, der er det sådan at lægerne de er, det er jo nærmest sådan at de bliver betragtet som små guder, sådan der ikke, eller når de kommer hen til terapeuter, så gør de kun hvad terapeuten siger, ikke. Og der er jo mange flere der i dag er sådan, der er… folk tager mere ansvar selv i dag.

RSH: Er det noget generelt det her med, I oplever med autoritet, at det er noget der er mindre af?

PHYSIOTHERAPIST 2: Jeg tænker at folk undersøger mere og mere selv. Og har en måske, allerede på forhånd en ide om hvad GLAD er inden, som I selv nævner, at der er nogle der måske bliver lidt irriterede over at "der står jo GLAD", det er det vi forventer. Øh, men fordi I ikke lige har kurset kan I ikke sige præcis at det er den øh.

PHYSIOTHERAPIST 5 og PHYSIOTHERAPIST 6 enige.

PHYSIOTHERAPIST 6: Ja, men der er stadig mange der kommer, altså det kan jeg godt følge, altså der er nogle der kommer og fornemmer, altså man lurer hurtigt en type, og der er mange der kommer, bum, det skal være sådan ikke. Også er der også rigtig mange der kommer og, åh du er fys, red mig ikke og øh, jeg har av av av av, og du ved ikke. Altså det er, det fornemmer man også, der er mange der kommer virkelig og har brug for hjælp med smerter. Altså som virkelig håber inderligt på at de kan få noget hjælp til at lindre smerte eller komme til at kunne nogle ting igen, øh som, hvor de egentlig er autoritetstro fornemmer jeg.

RSH: Ja, men denne der med de kommer og, du siger "åh du er fys, helbred mig", hvordan har du eller I det med den tilgang?

PHYSIOTHERAPIST 6: Ikke altid særlig godt når du ved, at de komme med nogle kroniske ting hvor du ved at du har rigtig svært ved og øh, hjælpe dem med alle deres problemer, ikke. Det er også derfor man skal prøve og øh, og sammen finde ud af, hvad kan jeg gøre noget ved og hvad kan

jeg ikke gøre noget ved. Hvad kan vi, hvad er realistisk for os og nå, ikke. Sådan plejer jeg i hvert fald gerne og ville stille det op, uden og være pessimist men sige vi sætter et mål ikke, altså.

PHYSIOTHERAPIST 2: Og allerede der lægge noget af ansvaret hen til dem selv også. Tænker jeg i hvert fald.

PHYSIOTHERAPIST 5: Ja, så længe at de ikke bare tror at de kommer og så laver vi noget manuelt…

PHYSIOTHERAPIST 2: Også ligger de sig bare på briksen og kan blive kurreret.

PHYSIOTHERAPIST 5: Ja, det er der også nogle der tror.

PHYSIOTHERAPIST 1: Og der synes jeg det er vigtig med undervisning af dem også, altså i… Det gør vi rigtig meget ud af i pacing principperne med hvor meget hvile, hvor meget aktivitet. Og hvordan kan jeg mærke når min krop siger nu har lavet for meget, og hvad skal jeg så gøre. Hvordan kommer jeg videre, sådan at de bliver selvhjulpne i det. Det synes jeg giver rigtig rigtig god mening. Øh, og der er også nogle der kommer som er meget utålmodige og bare gerne vil i gang og derfor knokler al for meget på, også kommer de jo selvfølgelig og har ondt bagefter, ikke. Så jeg synes det giver rigtig god mening og fortælle dem om pacing. Både til dem der er for lidt aktive og til dem der er for meget aktive.

PHYSIOTHERAPIST 2: Jeg kommer lige til at tænke ift. det her projekt og det der. De bliver vel ikke ekskluderet, hvis de laver noget træning selv ud over, eller hvordan? Er det et spørgsmål de bliver stillet?

RSH: Øhhh, kriteriet hedder bare at de ikke må lave quadriceps-træning.

PHYSIOTHERAPIST 2: Okay.

RSH: De må gerne træne sådan som de plejer, og de må også gerne starte på noget nyt i virkeligheden. Men sådan helt specifikt knæ-ekstensionstræning det må de ikke lave.

PHYSIOTHERAPIST 2: Okay.

RSH: Hvordan med det her, xx du snakkede meget om det her pacing. Kan du prøve at fortælle lidt mere om det, hvordan det bliver brugt? Eller fordele, eller ulemper, eller hvordan du oplever det.

PHYSIOTHERAPIST 1: Jeg bruger dem faktisk, altså jeg bruger dem næsten til alle som jeg har inde. Øh, jeg har meget ortopædkirurgiske, og der har jeg sådan helt generelt sådan et stykke papir, hvor der er sådan to forskellige kurver på, hvor der er et dårligt eksempel på hvordan man kan gøre og hvor der er et godt eksempel på det. Og dem gennemgår vi så, og allerede når de kommer, det kunne være folk med et nyt knæ, jamen så har de allerede været ude og prøve den dårlige kurve af. De har bare ikke lige vidst at det var det, så nu for vi sat nogle ord på hvad det handler om. Øhm, og hvad gør jeg så når jeg for så ondt. Øhm, nå men så er det noget med og afbetale med og aflaste, eller, sådan på den måde. Jeg bruger også nogle gange det man kan kalde en belastningskonto, hvor

man kan se at man har… Du har f.eks. 1000 kr. til rådighed i dag på dit gode knæ og 100 kr. på dit nyopererede knæ. Og alt hvad du laver i løbet af dagen, det koster penge, ikke. Det koster noget og gå på trapper og stå i køkkenet, og sådan på den måde. Det giver en meget pædagogisk forståelsesmodel for dem, som de meget meget nemt kan overføre og bruge lige med det samme. Og jeg kan bruge det når de kommer til træning, når de siger jamen jeg har simpelthen så ondt i dag, også kan vi jo snakke om at de har for lidt penge på kontoen til at træne i dag, fordi de brugte for mange penge i går og er ved at afbetale for det de har lavet. Det giver sådan et andet, sådan et fælles sprog om smerter og aktivitet.

PHYSIOTHERAPIST 2: Og et simpelt supplement, som lagt de fleste kan sætte sig ind i med nogle gode pædagogiske kurver eller regnskab. Så det er anvendeligt for de fleste.

PHYSIOTHERAPIST 1: Meget simpelt.

RSH: <u>Er det også noget I andre bruger?</u>

PHYSIOTHERAPIST 4: Nej. Jeg er faktisk lige ved og skrive det, for jeg bruger det ikke.

PHYSIOTHERAPIST 3: Altså lige til det med afbetaling og sådan noget, den har jeg ikke brugt. Og det kan man da forholde sig til.

PHYSIOTHERAPIST 5: Jeg synes også det lyder meget godt.

PHYSIOTHERAPIST 1: Det er utrolig simpelt. Og bare det at få sat ord på. I bruger det jo sikkert også i dagligdagen bare ikke så specifikt og så undervisningsagtigt. Øh, men det fungerer skide godt. Og den for de lige når de starter første gang. Også, og på holdet kan de jo også sige til hinanden, ”nå men du har ingen penge i dag”, eller, altså på den måde kan de, vi skaber sådan et sprog om det.

PHYSIOTHERAPIST 5: Det er en god ide.

PHYSIOTHERAPIST 3: Vi har haft nogle fysser på det her handleplans kursus, hvor de skulle øh, de var på kursus i at lave handleplaner med patienterne. Også, det er der jo ikke noget nyt i, der er det her med mål og delmål, nu kalder man det bare handleplaner, man bruger nogle andre ord. Øh, men der var en ting. Det her med at patienten, når de havde lavet de som man i gamle dage kaldte et delmål, altså en handleplan nu, så skulle patienten på en skala fra 0-10 score hvad er sandsynligheden for at jeg når det her mål. Altså det her med at vi tit laver delmål med dem, men hvis de ikke, hvis de sætter et tal under 5, så skal vi ikke gå i gang med det alligevel. Øhm, fordi så er det ikke realistisk at vi når det. Men hvis de scorer over 5, så kan vi, øhm. For det er så nemt og lave et mål med borgeren og man kan også nemt sige at det har vi lavet sammen, men måske har jeg lagt ordene i munden på ham ikke. Det er altid sådan lidt, øhm, ”det er en god ide”, haha. Men hvis man så bagefter skal sige, ”også så skal vi lige sætte en score på”. ”Ah, men det er realistisk at jeg når det inden for en måned, det er en 4”. Godt, så tager vi den lige forfra igen, vi skal lave et nyt mål. Fordi, hvis du scorer under 5, så du ikke noget ud af det vel. Det var den skjulte motivation eller hvad man kan sige, der lige viste sig der.

PHYSIOTHERAPIST 1: Interessant.

JK: xx, hvis jeg skal være den hårde tidsholder, så tror jeg vi skal omkring i 6 og 7 ikke. Tænker du ikke det? Vi havde til klokken 11 ikke?

RSH. Jo. Jamen det er så fint. I havde ikke mere til denne lige nu? Godt, så går vi videre til en anden overskrift. Det handler lidt om denne her BandCizer eller teknologiunderstøttet træning sådan mere overordnet. Øh, og i og med i jo ikke har brugt det så længe, så handler det lidt om hvordan I tænker det her fungerer. Øh, så lidt hvordan I indtil videre har oplevet det sådan rent praktisk. Om den er let og bruge?

PHYSIOTHERAPIST 5: Det er den. Den er jo nem og sætte op. Altså, og der er jo ikke så mange ben i den, især ikke hvis du bruger ordentlig tid på, sammen med øh borgeren til og prøve det igennem. Altså ofte så når man tænker på at det er noget teknologi, altså, baseret studie, studie i skal lave, altså så øh, er det forholdsvis nemt for den enkelte og leve op til, altså og bruge det. Øh, hvad man ellers har været ude for. Hvad man har prøvet med iPads og des lige. Øh, som godt kan være en del sværere, så det burde, det er realistisk at folk kan finde ud af at gøre det ordenligt, tænker jeg.

RSH: Ja. Har I andre også samme holdning til det?

PHYSIOTHERAPIST 2: Tænker også den er ret simpel og bruge. Og nu den information vi giver dem er jo bare at den registrerer at de træner. Og ikke noget med at de kan se belastningen eller noget som helst. Så den spiller på en eller anden måde en lille rolle, synes jeg indtil videre ift. den samlede instruktion.

RSH: Tror du at det er en god eller en dårlig ting, at det er en lille…

PHYSIOTHERAPIST 2: Hmmm, altså jeg tror det er både og med det der. Nogle kan måske føle at det er overvågning ikke, og andre ”det er bare sådan det er”. Eller det er i hvert fald ikke det vi ser med ICUA, som xx talte om før ik. Det der med at der er nogle der kan overvåge eller følge med i hjemmetræningen. For nogle er det en motivation og for andre spiller det en mindre rolle. Fordi de ville have gjort det alligevel.

RSH: Ja. Og det leder lidt ind til det andet. Altså, hvordan tror I patienterne vil have det med, at der er den her lille teknologi-dims med?

PHYSIOTHERAPIST 4: Jeg tror ikke den gør den store forskel. Fordi den er så simpel, og den er så, den er så ufarlig den ting her. Så den er ikke… Jeg kan ikke forstille mig at et flertal af folk er øh, imod den der. Eller bliver bange for den eller synes den er…

RSH: Dem der så skulle være imod den, hvorfor kunne det være?

PHYSIOTHERAPIST 4: Jamen, det er nok mest de folk som øh, som synes at vi i forvejen bliver overvåget og overgennemlyttet og alting, fordi at alle ved hvor man er henne, når man tænder sin telefon. Så, det er vel mere ovre i sølvpapirshalvtaget-folket sådan ikke (haha). Jeg tror at majoriteten, de tror jeg ikke er øh…

PHYSIOTHERAPIST 2: Det tror jeg heller ikke.

PHYSIOTHERAPIST 1: Det tror jeg heller ikke er noget problem.

PHYSIOTHERAPIST 5: Nej.

RSH: Og sådan rent praktisk for patienterne når de jo så forlader jer, så står de jo alene med den. Tror I de kan finde ud af det?

PHYSIOTHERAPIST 1: Og sætte den på og tage den af?

RSH: Ja.

PHYSIOTHERAPIST 1: Det tror jeg godt de kan.

PHYSIOTHERAPIST 2: Mmmmm.

PHYSIOTHERAPIST 5: Det eneste der skulle være, var vidst den gik i stykker af en eller anden grund, altså.

PHYSIOTHERAPIST 1: Ja, de for jo ikke feed-back fra den på nogen måde.

PHYSIOTHERAPIST 2: De skal lige ind i vanen med at den lige bliver opladt.

PHYSIOTHERAPIST 1: Altså, det kan godt være at de glemme og tage den af, tænker jeg. Men altså, jeg ved det ikke. Jeg tænker ikke det er det store problem.

PHYSIOTHERAPIST 2: Det forestiller jeg mig heller ikke.

PHYSIOTHERAPIST 3: Skal den ikke i opladeren hver gang de har…

PHYSIOTHERAPIST 1: Jo, det er instruktionen i hvert fald.

PHYSIOTHERAPIST 3: Er den blevet opladet når du (xx) giver den til dem?

RSH: Jo, den er helt fuld, når de for den fra mig af.

PHYSIOTHERAPIST 3: For ellers kunne første test jo være om de kommer med den opladet til fysioterapeuten.

RSH: Ja, det er også lidt risky (haha). Okay…

PHYSIOTHERAPIST 1: Det virker meget simplet.

RSH: Ja. Hvad hvis det skulle, øhm, skal det, er der mulighed for at tilpasses det så det blev bedre end det er på nuværende tidspunkt?

PHYSIOTHERAPIST 2: Altså, brugen af BandCizeren eller hvad?

RSH: Ja, generelt, ja brugen af den, og hvad skal man kalde det, ”det at den er der”, hvis I kan følge mig i det. Og brugen den, det er jo det med og sætte den på, men at de skal huske den og forholde sig lidt til den.

PHYSIOTHERAPIST 5: Det er svært og sige, vi har jo ikke rigtig så meget erfaring med det endnu, øh…

PHYSIOTHERAPIST 2: Nu må vi se når de kommer igen, om de har brugt den

PHYSIOTHERAPIST 1: Vi kan jo heller ikke se om de har husket og sætte den på. Det er jo dig (xx) der kan se det.

RSH: Ja, og det kan jeg først se når de er færdige faktisk.

PHYSIOTHERAPIST 1: Nå ok.

PHYSIOTHERAPIST 2: Du for ikke nogle, øøh, løbende?

RSH: Nej, men det leder lidt videre til det næste…

PHYSIOTHERAPIST 2: Og hvad er det så præcist du kan sidde og følge med i? Er det så hvor udstrakt elastikken er?

RSH: Ja, men det trækker vi ikke ud, for det er ikke, det kan vi ikke vide helt endnu hvad det helt svarer til. Det er det ikke helt nok udviklet til. Men det jeg kan sige det er, at jeg for datoen, dvs. dagen, også kan jeg jo se hvor mange datostempler er der i løbet af en uge, ift. hvor mange de skulle lave. Også kan jeg gå ind på den dato og se hvor mange gentagelser de har lavet. Så der kommer bare til at køre sådan en kurve derhen af, hvor en bølge er en gentagelse, også kan jeg jo tælle dem sammen. Ift. hvor mange de skulle lave og hvor mange sæt er der så. Så det er de tre ting der kommer ud af det. Det var lidt omkring det praktiske. Så er der det… Hvis nu I havde det her data til rådighed. Enten at I løbende fik det, eller at når de kom ind til de der booster besøg. Ville det så være… Er det et potentiale I kunne se noget positivt i? At I kunne få denne her information.

PHYSIOTHERAPIST 2: Det er mere at hvis nogle de ikke har trænet f.eks., så finde ud af årsagen til det.

RSH: Ja, så du ville bruge informationen til, at du ville vide at vedkommende rent faktisk ikke havde trænet.

PHYSIOTHERAPIST 2. Ja. Eller kunne følge op på at de har gjort det som de skal, og skal forsætte. Hvis der er nogle der følger instruktionen.

PHYSIOTHERAPIST 6: Den er også, den er lidt svær ikke og bruge, altså de data der med at man følger med i om de gør det de skal ikke, via overvågning. Man er ude i noget, hvornår man skal holde øje med, eller om man skal holde øje med folk på den måde. Eller om deres ord er nok i sig selv eller. Der er fordele og ulemper.

RSH: Ja, hvad tænker du? Kan du bruge at uddybe det der med hvordan, det lyder som om hvordan du har det med at skulle (”overvåge”)…

PHYSIOTHERAPIST 6: Ja, det er det der med hvordan man, så skulle det i hvert fald hvordan man… Ah, hvis du har lavet en ordentlig aftale fra start af måske, og du snakker om hvad den registrerer når du gør, og sådan noget ikke. Os hvordan man leverer den ikke. Nå men jeg kan se at du ikke har lavet det du skal eller. Også kan man selvfølgelig spørge ind til hvorfor og sådan noget men, nogle vil måske føle at det er sådan lidt overskridende ikke, altså at blive konfronteret med at de ikke måske har lavet det de skal, ikke. Det kunne jeg forestille mig.

PHYSIOTHERAPIST 5: Den kan være meget god ift. at kunne tilpasse hjemmetræningen i hvert fald. Om du siger de skal lave det 3-4 gange om ugen, og de måske kun laver det 2 reelt, sådan gennemsnittet. Om du så skal give 6 gange om ugen eller hvordan man skal. Det synes jeg den er meget god til at kunne finde ud af det.

PHYSIOTHERAPIST 6: Ja.

PHYSIOTHERAPIST 1: Jeg tænker, altså nu har vi det jo allerede med IKURA, som vi træner og kan gå ind og se. Og i en travl hverdag… Jeg for bare ikke kigget nok. Altså, fordi det kræver jo også noget tid at du går ind. Altså, hvis jeg skal sidde og kigge og tæller takker, og hvor mange gange om ugen og sådan, det for jeg ikke gjort.

PHYSIOTHERAPIST 2: Jeg tænker heller ikke det var relevant for lige projektet her, men ift. BandCizeren hvis man skulle kunne bruge sådan en til noget i andre typer…

RSH: Ja, det er lidt tænkt ud over projektet. Senere hen hvis det var noget information I faktisk skulle have på mere daglig basis.

PHYSIOTHERAPIST 2: Så er det igen det der, hvis alt skal være tidsbesparende ift. kontakten med patienterne og borgerne, så kommer der jo al den tid, hvor man skal sidde og netop monitorere og følge med i øvelserne. Og hvor tidsbesparende er det så lige?

RSH: Ja.

PHYSIOTHERAPIST 2: Jeg synes også hurtigt, at det kan blive kedeligere. F.eks. med IKURA ikke, hvis man skal sidde og kunne korrigere øvelser eller antal.

RSH: Ja, hvis nu du kunne vælge. Ville du så tage mere patientkontakt som den ene model, eller en med mindre patientkontakt plus mere data?

PHYSIOTHERAPIST 2: Mere patientkontakt, mindre data. Det er meget sjovere.

PHYSIOTHERAPIST 1: Ja.

PHYSIOTHERAPIST 3: Ja, det tænker jeg også.

RSH: Er det generelt (jeres holdning)?

Alle: Ja.

PHYSIOTHERAPIST 6: Det er nok derfor man er i faget tror jeg. Altså, mange vælger (fys) fordi de godt kan lide og arbejde med mennesker.

Flere: Ja.

PHYSIOTHERAPIST 6: Altså, men havde det fungeret at det havde været nemmere og aflæse, havde det været meget mere hurtigt og lige og overskue rent hvor mange gentagelser fordelt over hvor mange dage og sådan. Havde det været sådan havde det. Altså hvis det bare stod på sådan en lille monitor eller sådan en skærm eller hvad ved jeg, ikke. Så havde det været skide smart at man lige kunne se. Nå, ok, øhhh, de for måske ikke lavet det, eller, det de har skulle, men de har stadig fremgang, eller modsat ikke, øh. Det kunne da være meget interessant.

RSH: Ja.

PHYSIOTHERAPIST 6: Så tror jeg, at hvis det var nemmere at gå til, hvis det var nemt. Nemmere. Nu ved jeg ikke hvordan det er at gå til, men hvis det er nemt…

PHYSIOTHERAPIST 1: Det er mere det, hvis man skal til og ind i et nyt computersystem, også ind og klik, og klik, og klik, og klik. Også kan du alligevel ikke rigtigt få det overblik som du gerne vil. Altså, det skal i hvert fald meget tilgængeligt, tænker jeg hvis jeg skal bruge det i min dagligdag. Og ellers vil jeg jo gerne, som udgangspunkt, tro på det som patienten siger.

RSH: Er det også noget som I andre mener? At det skal være nemt, hvis det var der.

PHYSIOTHERAPIST 5: Helt sikkert.

Andre: Ja.

PHYSIOTHERAPIST 2: Hjælper vi dem mere på vej, ved at de, så længe de går hos os føler sig lidt tvunget måske eller overvåget til, så de for udført øvelserne. Men lige så snart de så slutter og vi tager alle de der remedierne fra dem, om de så bare tænker, nå men nu er der ingen der holder øje. Altså, så kan vi ligeså godt, eller tænker jeg også at så vil jeg bare tro på det de siger.

RSH: Ja. Nu det du siger der xx det er, kunne, tror I de vil træne mere fordi at denne her (BandCizeren) den er med dem, end de ellers ville have gjort?

PHYSIOTHERAPIST 2: Jeg tror dem der bliver inkluderet har en ide om at de vil jo, nu har de jo sagt ja, så nu vil de gerne deltage og…

RSH: Uanset om den (BandCizeren) er der eller ej?

PHYSIOTHERAPIST 2: Ja.

PHYSIOTHERAPIST 3: Ja. Jeg tror ikke det er BandCizeren der for dem til at lave øvelsen hver dag.

PHYSIOTHERAPIST 2: Nej.

PHYSIOTHERAPIST 3: Jeg tror, at det er det at de ved, at de er en del af et projekt.

PHYSIOTHERAPIST 2: Ja. Og de har ondt, og de vil gerne have det bedre.

PHYSIOTHERAPIST 3: Vi giver dem kun én øvelse, de har ikke andet. Altså, det tror jeg også er det der gør at de for den lavet. En følelse af at de er en del af et projekt, derfor skal de lave øvelsen. Jeg tror også følelsen af at jeg skal lave øvelsen, for ellers kan de jo ikke bruge mig til noget, også er det spild af tid det hele. Også det her med at de har én øvelse. Så bliver den meget vigtig.

RSH: Er det en potentiel fordel ved at der kun er en øvelse?

Mange: Ja.

PHYSIOTHERAPIST 3: Ja, altså lige i det her projekt så tror jeg.

PHYSIOTHERAPIST 1: Ja, det er nemmere og overskue…

Flere taler i munden på hinanden, ikke til at høre.

RSH: Er det noget i alle sammen tror kan være en fordel?

Alle: Ja.

PHYSIOTHERAPIST 6: Ja, helt sikkert.

RSH: Kunne der så også være en ulempe i, vi har nok været lidt inde på det, men det der med at der trods alt kun er den ene øvelse?

PHYSIOTHERAPIST 6: Ja, det tror jeg også, hvis nu at de ikke føler nogle fremskridt, øh hverken ift. altså ift. smerter eller styrke, eller… Altså så tænker de nok, altså hvis det ikke giver mening for dem og de måske har en ide om at de kunne noget andet (træning), tænker jeg også. Så kan det godt være at de bliver demotiveret.

RSH: Ja.

PHYSIOTHERAPIST 6: Nogle vil måske gøre det. Hvilket så kan føre til, at de ikke er lige så flittige med og få lavet det ikke.

RSH: Så det er, så umiddelbart tænker du at den er meget afhængig af, altså det er alt eller intet nærmest, med den øvelse her?

PHYSIOTHERAPIST 6: Med nogle tror jeg godt det kunne være sådan. Altså sådan vil det altid være, men ja det kunne det godt være. Hvis nu at de ikke mærker nogen effekt og der er jo, hvis man nu er en utålmodig sjæl ikke, og man går noget tid, hvis du hurtigt kommer op, altså hvis du går i de fire uger efter vi har set dem og de ikke mærker en forskel, så kan det godt være at man tænker, jeg sidder her og laver den ene øvelse. Især hvis det er nogle der har, måske, altså hvis der

er nogle der måske har været vandt til og træne og tænker jeg der måske, tænker jeg, jeg kunne måske også lave nogle andre ting. Kunne man forestille sig. Også vil der uden tvivl også være dem, som så også for ondt også bare af pligtsomhed, du ved, bliver ved, altså fordi nu har de meldt sig til noget også kører de det til ende ikke.

PHYSIOTHERAPIST 2: Jeg tænker også om der er nogle der kunne blive inkluderet der måske har nogle andre problematikker også. Øh, i forbindelse med knæet, som… Den ene jeg så, det lød som om at det var, der var nogle delte meninger hos alle de kirurger hun havde været hos, også lige ham på xx havde sagt, det er artrose, øhm du skal være med i det her projekt. Og hun havde allerede gået til fysioterapi, for den ene mente at det var en fibersprængning i læggen, hun kunne ikke strække knæet helt, øh, så om der kunne være noget bias der ift. til lige den simple øvelse og hvad de ellers har, måske har, af udfordringer og skavanker.

RSH: Ja, er det øhm, det her med at de kommer ind og har fået forskellige ting at vide og forskellige sundhedsfaglige personer. Er det noget I oplever ofte?

Blandede svar, både ja og nej

PHYSIOTHERAPIST 2: Ikke så meget lige de diagnoser vi ser, fordi så er de ofte opereret, så har de fået det nye knæ eller sat knoglerne sammen.

PHYSIOTHERAPIST 1: Ja, vi har ikke så mange præ-operative, så det er måske nok derfor.

RSH: Men I sagde lidt ja herovre fra…

PHYSIOTHERAPIST 5: Ja, det sker nogle gange hvor at de har fået at vide at det er menisk, eller noget korsbånd, eller et eller andet. Og så kommer de også ind til sidst og for at vide at det er noget slid, så, også prøver vi med det.

RSH: Og hvordan har I det med det? Altså at de kommer ind der og er lidt forvirrede eller hvordan man kan opleve dem? Opgivende.

PHYSIOTHERAPIST 6: Ja, der er i hvert fald nogle der kommer ind med en følelse af at øh, at der ikke altid er helt så godt styr på det. Altså har jeg fornemmelsen af.

PHYSIOTHERAPIST 5: Ja, en mistillid til systemet på en eller anden måde.

PHYSIOTHERAPIST 6: Øhm, som ikke, altså der er mange, altså lige for en måneds tid siden der havde jeg sådan 2-3 stykker i træk til startsamtaler hvor at, øh. Hvor de kommer fra en ortopædkirurg som de føler, hvor de ikke er blevet hørt. Altså sådan, hvor jeg egentlig godt kan forstå lidt, altså hvor jeg, jeg prøver ikke at tage parti, men og være objektiv, men jeg kan godt se fra deres syn, altså der hvor de sidder ikke, at de kommer ind til en lidt forvildet 10 minutters samtale ikke, uden at der er blevet læst journaler og sådan noget ikke, og bla bla bla bla, du skal have noget GLAD træning eller... Og bare swup ud med dig, ikke. Også kører den ikke. Øh, også når man så går lidt i dybden så finder man ud af, at det ikke er altid nødvendigvis at man er helt enig i det der står i genoptræningsplanen.

RSH: Og hvordan har du det så med det når du sidder med den fornemmelse, i ikke og være enig i hvad der måske er kommet fra hospitalet?

PHYSIOTHERAPIST 6: Altså, man prøver at forstå at det, hvorfor det sker ikke. Øh, man kan godt se at der jo er en grund til at ting de sker, at det må være fordi der er et pres et sted. Og nogle gange så er der også nogle patienter der er sværere end andre. Men jeg kan da også godt se det fra deres side af, øh patienten, at det må være død frustrerende og komme med noget hvor man tænker at det burde være lige til. Altså, jeg kan ikke forstå hvorfor de ikke scanner mig. Eller sådan du ved, sådan ting ikke. Også jeg tænker, der er sikkert en god grund til det. Det er jo ikke alting, altså jeg ved jo ikke alt der foregår hvorfor de vil det ene og ikke det andet altså. Ja, man kan i hvert fald godt forstå det nogle gange, at de føler sig… Det er mere det der med, at jeg kan ikke forstå at man ikke tager sig mere tid til lige og… Mange gange så handler det om en kommunikation faktisk sådan som jeg ser det. Det er kommunikationen der ikke bliver brugt tid nok på.

RSH: Mellem lægen og patienten?

PHYSIOTHERAPIST 6: Ja, det sådan ser jeg det mange gange, altså hvis man bare brugte 5 minutter ekstra ikke på lige og tage de. Nogle gange så er de mildt ud sagt, altså snotforvirrede. Øh, de borgere der så kommer ud.

RSH: Er det noget I andre også oplever?

PHYSIOTHERAPIST 2: Ja.

PHYSIOTHERAPIST 4: Vi for efterhånden nogle genoptræningsplaner, som vi knapt nok kan forstå. Altså hvor beskrivelsen, som lægen er kommet med er ufyldestgørende og øh, og faktisk kan man læse at de har ikke forstået hvad patienten egentlig, hvad de er her for. Så derfor sender de dem videre ikke. Så er de kommet af med dem, også kommer de ud til os.

PHYSIOTHERAPIST 3: Altså må jeg komme med et rigtig godt eksempel på, at vi nogle gange kan have svært ved og finde vores rolle som fysioterapeut. Jeg skal have en patient i morgen, som øhm har en achillessene tendinit. Lægen skriver i journalen, eller i GOP'en, hun skal lave udspænding excentrisk uden dorsalfleksion i anklen. Patienten har fået en artikel med hjem, som hun vil komme med til træning. Og jeg skal som terapeut sørge for at hun udfører det der står i artiklen rigtigt. Jeg har ikke fået nogen artikel, den ser jeg først når borgeren kommer i morgen. Altså det her med, det kan godt være at patienterne er lidt forvirrede. Det synes jeg altså også vi kan være nogle gange. Han (lægen) kunne da i det mindste have sendt artiklen inden.

PHYSIOTHERAPIST 2: Det er også bare meget og diktere. Altså han har lavet en undersøgelse, men det kan være at du ser noget andet, eller finder noget andet, eller…

PHYSIOTHERAPIST 3: Lige præcis.

PHYSIOTHERAPIST 1: Ja, og hvad er årsagen til at du ikke må lave dorsalfleksion, og hvordan for man så lavet den øvelse?

PHYSIOTHERAPIST 3: Ja, lige præcis ikke.

RSH: Hvordan har I det med de der eksempler, når I for GOP’er som er svære og forstå?

PHYSIOTHERAPIST 4: Ryster lidt på hovedet også tager vi en ordentlig snak med vedkommende når de kommer ikke. Altså, for tit er de jo ikke blevet taget ordentlig. Altså, tit har de jo ikke fundet hinanden, sådan lægen og klienten, så tager jeg en lang snak med de her mennesker, hvad handler der her så om? Fordi, der kommer flere og flere af dem. Jeg tror vi begyndte og lave en mappe en overgang, sådan hvor puttede dem i som var helt gak.

PHYSIOTHERAPIST 2: Vi oplever lige ift., det er måske ikke en dissideret uoverensstemmelse, men nogle af dem der har, øhm, ankelfrakturer. Der for de at vide at nå de har været ude til kontrol, nu skal du bare smide stokkene, du skal ud og gå, og du skal gøre alt hvad du kan. Så kommer de hos os haltende, kæmpe hævelser og smerter, og bliver måske endda meget demotiverede over at vi siger, det duer ikke det her. Du skal begynde og bruge stokkene igen. Du skal ikke gå så langt, du skal have lidt mere hvile, øh, a pro pro pacing osv. Også sidder de, jamen jeg har jo lige hørt det modsatte.

RSH: Mmmm.

PHYSIOTHERAPIST 2: Øhm, så der siger vi jo noget andet end det den øverste i hierarkiet ligesom har givet besked om.

PHYSIOTHERAPIST 3: Og omvendt kan det jo også være, altså nogle gange når de så kommer og har været dybt uenige med en læge om det nu var menisken eller brusken. Og de kommer og nærmest er oppe og skændes med lægen, til os. Så prøver jeg og sige til dem måske er det ikke så vigtigt om det er brusken eller menisken, fordi det vi kan lave hernede (i fys regi) det er knæbøjninger, og vi skal nok få finde den knæbøjning som hjælper dig. Uanset om det er den ene eller den anden. Altså hvor de er blevet for fokuserede på diagnosen, altså fordi, så er fysioterapi jo heller ikke mere indviklet end at vi skal nok finde en træning der passer, også prøver vi at smide diagnosen lidt væk.

PHYSIOTHERAPIST 2: Mmmmm, men det er nok ofte et led i frustrationen, hvis det er dem der har været hos, øh, flere forskellige kirurger ikke, og kun bliver mere og mere forvirrede og måske for det værre og værre med det knæ, eller hvad det nu må være ikke.

PHYSIOTHERAPIST 3: ”Det fylder for meget i hovedet hvad det er jeg fejler”.

PHYSIOTHERAPIST 1: Men det er nok også lidt en udløber af, hvor du siger xx, at de måske ikke har følt sig mødt eller hørt, når de så er kommet til denne her (samtale med lægen). De har gået og ventet lang tid på at komme til kirurgen, og nu er de endelig kommet der, også bliver de ikke hørt i det som de har at sige, og det tager måske bare 5 minutter ekstra lige og føle sig hørt. Eller lige at lægen tier stille et øjeblik og de for lov til at tale om det de har, også snakke i stedet for at der bare bliver sagt et eller andet også er de ude af døren.

PHYSIOTHERAPIST 6: Ja, jeg sidder i hvert fald nogle gange med fornemmelsen af at øh, at de kører, at nu har jeg ikke været til sådan en konsultation, jo jeg har været til nogle, men men at de lirer noget af på 10 minutter ikke og bam bam bam (knips). Det sker bare…

PHYSIOTHERAPIST 2: Ja, det går lidt hurtigt.

PHYSIOTHERAPIST 1: Og lægen sidder måske i forvejen foran en computer imens de snakker sammen ikke. Så den der kommunikation/mødet det er der ikke rigtig.

PHYSIOTHERAPIST 6: Nej.

RSH: Men hvordan har I den med denne her rolle? Det lyder som om I for en rolle hvor I måske kan snakke lidt mere med patienten. Hører lidt mere om hvordan og hvorledes. Hvordan har I det med at have den rolle i systemet?

PHYSIOTHERAPIST 6: Altså de gange… jeg har haft gode oplevelser med at man kan ligesom føle at man kan få reddet nogle tråde ud og sagt, altså sådan, nu er det ikke værre og, men man skal selvfølgelig bruge noget ekstra tid på det ikke. Men øhm, ofte så går det jo godt, altså så føler de sig sådan, øhm, taget hånd om. Det føles da også godt at man kan, altså give dem en håbenlig god oplevelse af at man bruger sig tid til og prøve at forstå hvad det er, at der er deres problem. Også og forstå hele deres situation, ikke. Og det har vi vel nok også lidt mere tid til. Eller vi tager os bare mere tid til det end måske læger har. Øh, hvad hedder det, end hvad læger har historik for at gøre. Fordi de skal gå hurtigere ikke, de skal køre mange flere igennem. Øhm, så har man mulighed for at rette op på det i hvert fald. Øh, og finde ud af hvad det er ikke. Det er så et problem når man for nogle ind med en diagnose hvor man kan se, at det har ikke noget med det at gøre, ikke. Altså, hvor man skal finde ud af, hvad man gør. Øh, om man skal have en ny GOP eller, hvad man så gør.

PHYSIOTHERAPIST 1: Jeg synes det er meget meningsfuldt for mig, som terapeut og som menneske i det hele taget at få… Specielt det første møde når de kommer ind, f.eks. har de lavet som xx siger haft en eller anden ankelfraktur eller de har fået et nyt knæ, også kommer de ind også for vi en snak om hvad det er de skal gøre, og ”det her det er helt normalt de smerter du har eller den hævelse”, og hvad gør jeg så når jeg har det? Og de går derfra, altså nogle gange, som et helt andet menneske. Men i hvert fald med en helt anden indsigt i den problematik, som de har og hvad de selv kan gøre ved det. Og det er ikke noget der er farligt og det skal nok gå godt. Der er lys for enden af tunnelen, ikke. Altså det, jeg synes vi gør en, jeg tror vi gør en kæmpestor forskel for dem der kommer ind. Og har følt sig lidt alene i det, som de har været i.

PHYSIOTHERAPIST 2: Jeg tænker også nogle gange og kunne få aflivet nogle af alle de frustrationer. Netop kunne finde årsagen til noget der måske er simpelt, men som patienten ikke har været informeret om tidligere. Øh, man kan komme langt med det. Og at der netop er mere tid en kirurgerne har til noget information.

RSH: Ja.

PHYSIOTHERAPIST 6: Nogle gange så har de hængt sig op i et par enkelte ord, som kirurgen har sagt, som bare hænger fast. Jeg prøvede sådan med en der havde et kompartmentsyndrom og var blevet opereret sådan tre steder, og han havde sådan fortalt et eller andet, som måske via operationen med at han havde syet noget fast i noget væv imellem knoglerne i underbenet. Og det tænke hun var, det havde hun fundet frem til var en eller anden dårlig ting, og mente det var derfor at det gik så dårligt med foden og sådan noget. Det er jo bare et eller andet han bare lige hurtigt har slynget ud, og i hendes ører der lød det som om det var en fejl han havde gjort. Som om han havde… Men det havde han så ikke fået forklaret ordenligt, at det egentlig var en del af operationen for at holde det hele på plads. Så hun kom og hun troede at det hele var på grund af det, altså. Det var sådan lidt, at der manglede i hvert fald noget der. Men nogle gange synes jeg også det er meget rart, f.eks. med TKA'er, at nogle gange når de kommer og siger at, at de har gået hos os i 3 måneder og de synes ikke at det går som det skal, og der er stadig en lille smule hævelse og sådan noget, så er det også meget rart og kunne sige, "nå men lægen han sagde jo også at der gik et år". Også, så er det okay. Altså, når man ligesom kan sige det ikke. Det er som om at det betyder lidt mere for dem. Så kan man sige "nå, men de læge sagde jo også at der gik et år", så kan man også bruge den på den måde i hvert fald.

PHYSIOTHERAPIST 2: Men nu går mange af, f.eks. lige TKA'erne og THA'erne på de der patient seminarer, hvilket jeg tænker er en stor fordel og de kan nå og fange mange. Og få forklaret nogle årsager og hvad de kan forvente sig. Øhm, også er der jo nogle der måske ikke for, eller deres udbytte er måske ikke så stort, så de kan komme ud til os og sige "oh, havde jeg bare vidst hvor voldsomt det var, så havde jeg aldrig gjort det", eller et eller ikke. Men jeg tror på, at netop de der patient seminarer kan gøre en forskel. Øh, de for i hvert fald fanget nogle.

PHYSIOTHERAPIST 1: Man ved så bare ikke hvor meget de snakker om smerter. Altså, om de så ikke for nogle kunder i butikken, hvis de fortæller hvor virkelig ondt det kan gøre bagefter.

PHYSIOTHERAPIST 2: Så de gør ikke alt. Og netop, de bliver, der er mange der stadig bliver overrasket over smerterne og øh…

RSH: Så i virkeligheden så…

PHYSIOTHERAPIST 2: Men jeg tror på at det er den rigtige vej.

RSH: Ja, det lyder som om at de her patientseminarer, skulle de så indeholde mere omkring, altså tegne et mindre gloriøst billede af det post-operative forløb?

PHYSIOTHERAPIST 1+2: Ja, mmmmm.

PHYSIOTHERAPIST 1: I hvert fald lige den første del af det. Fordi de første 14 dage/1 måned, det er altså rigtig hårdt for nogle. Altså, super super hårdt, altså hvor de ikke sover om natten, og derfor bliver fuldstændig tyndslidte, og grådlabile, og har det rigtig rigtig skidt. Men man skal måske bare have en vigtighed om at, hvor, altså, at de så skal opsøge egen læge og blive bedre smertedækket, hvis det er muligt.

RSH: Ja. Lad os lige holde en sidste lille pause, inde at vi tager, det sidste leder lige op til det.

**xx og jeg er gået for at snakke.**

PHYSIOTHERAPIST 5: Er der sket noget nyt på xx?

PHYSIOTHERAPIST 2: Er der sket noget nyt. Jamen vi er blevet slået sammen med forebygningscenteret.

PHYSIOTHERAPIST 5: Nå okay, og hvordan fungerer det?

PHYSIOTHERAPIST 2: Ja, det er der ingen der ved (haha). Der kom en melding oppe fra. Så i stedet for så har de lavet sådan et bydækkende diabetescenter.

PHYSIOTHERAPIST 5: Nå, okay. Spændende.

PHYSIOTHERAPIST 2: Også er hjerte- og KOL-delen ligesom fusioneret med det almene træningscenter, som vi kalder det.

PHYSIOTHERAPIST 5: Har I så fået nogle nye derud?

PHYSIOTHERAPIST 2: Ja, så har vi fået nogle af dem der var i det tidligere forebyggelsescenter. Der er rykket sammen med os.

PHYSIOTHERAPIST 5: Okay, dem tror jeg ikke jeg har mødt.

PHYSIOTHERAPIST 2: Nej, nogle af dem var oppe på anden sal hos os, og andre var nede på første sal.

PHYSIOTHERAPIST 5: Nå, okay.

PHYSIOTHERAPIST 2: Der er mange gode ideer ved det. Problemet er bare at det er så ugennemtænkt før det er sat i værk.

PHYSIOTHERAPIST 5: Var det det?

PHYSIOTHERAPIST 2: Ja.

PHYSIOTHERAPIST 5: Nå okay.

PHYSIOTHERAPIST 2: Så det var sådan fra den ene dag til den anden så rykker i fysisk sammen på kontorerne, også skal vi finde ud af hvordan og hvorledes tilbuddet skal være, undervejs. Så det er faktisk ret frustrerende, selvom der er gode ideer med det.

PHYSIOTHERAPIST 5: Ja, det kan jeg godt forstå. Det var i forvejen lidt presset derude kan jeg huske.

PHYSIOTHERAPIST 2: Ja, der er plads nok egentlig på kontorerne. Så de rammer er der. Det er mere sådan det frustrerende i, hvad er det vi skal gøre egentlig. Hvad er det der skal være anderledes? Hvad er det vi skal kunne tilbyde og…

PHYSIOTHERAPIST 5: Da jeg var der, mens jeg skrev bachelor, der var jeg der sådan to gange om ugen…

PHYSIOTHERAPIST 6: Det er altså lidt interessant at der alt sammen… Nu for vi selvfølgelig også mange genoptræningsplaner, så der vil jo komme nogle tilfælde med dårlig kommunikation ikke. Men jeg synes at alle sammen oplever det ikke, og det er sådan…

PHYSIOTHERAPIST 2: Det er jævnligt at jeg må ringe ud og beder om nogle supplerende oplysninger til de der GOP'er. Øh, fordi de simpelthen er for mangelfulde.

PHYSIOTHERAPIST 6: Ja.

PHYSIOTHERAPIST 5: Jeg fik også en der var blevet henvist til genoptræning. Men lægen skrev at hun ikke måtte begynder genoptræningen endnu, men han havde henvist hende. Og så er det sådan lidt, det eneste hun måtte lave det var sådan noget passiv bevægelighed, men alligevel ikke for meget og… Jeg vidste slet ikke hvad jeg måtte. Det var sådan virkelig spøjst. Der spurgte jeg hende også "hvad fik du egentlig at vide?". Det kunne hun heller ikke huske. Hun havde bare skrevet det på sådan en almindelig seddel bare i hånden. Er det ikke spøjst?

PHYSIOTHERAPIST 2: Ja, okay. Det virker lidt ufærdigt.

PHYSIOTHERAPIST 5: Det var også hende med kompartmentsyndromet faktisk. Hun var også en der kunne forstå mange ting. Men alligevel det var altså. Det kunne jeg heller ikke forstå.

PHYSIOTHERAPIST 2: Hun blev ret meget tabt lyder det som, ja.

PHYSIOTHERAPIST 5: Ja.

PHYSIOTHERAPIST 6: Det er utroligt hvor meget kommunikation det betyder ikke.

PHYSIOTHERAPIST 5: Ja.

PHYSIOTHERAPIST 2: Jeg tænker med nogle af TKA'erne, eller THA'erne, at det virker til at det nogle gange går så hurtigt at de. Også hvis kirurgen siger "ved du hvad, det bedste for dig det er en operation", "nå men hvis kirurgen siger det, så er det nok det". Jeg tænker sådan. Skulle der måske lige være sat ind med noget præ-operativ træning? Eller noget vægttab, øhm…

PHYSIOTHERAPIST 5: Og det kan også godt være at de ikke informerer så meget om det efterfølgende forløb, fordi de er bange for at folk de så siger nej.

PHYSIOTHERAPIST 2: Ja.

PHYSIOTHERAPIST 5: Altså, de vil jo gerne have så mange igennem som muligt.

PHYSIOTHERAPIST 2: Og nogle er helt sikkert så generet af det, og kan ikke sove om natten pga. smerter osv. Men nogle gange så synes jeg også det lader til at der nogle der ikke har været så generet. Eller der så i hvert fald måske lige er et tiltag der har skullet afprøves inden de giver dem det nye knæ.

PHYSIOTHERAPIST 5: Ja, der er nogle der kommer ud og ikke, altså heller ikke efter operationen har nogle smerter. Jeg kan huske jeg havde også en ældre herre på, hvad var han, på 75 eller sådan noget, som også kom ud. Han havde bare sådan en lille smule ondt når han havde spillet badminton i tre timer. Øh, og han blev opereret.

PHYSIOTHERAPIST 2: Også for han et nyt knæ ikke. Hvor man tænker, det var lidt overkill.

PHYSIOTHERAPIST 5: Han var jo selv meget kritisk ikke. Han ville jo gerne, så han pressede selv på. Så fik han et nyt knæ.

PHYSIOTHERAPIST 2: Ja.

PHYSIOTHERAPIST 5: Han var 73, han kunne mærke efter at han havde spillet badminton i tre timer, han kunne mærke en lille smule. Det ville han gerne gjort noget ved.

PHYSIOTHERAPIST 2: Det var måske ikke et helt nyt knæ der skulle til der.

PHYSIOTHERAPIST 5: Nej, men det fik han.

PHYSIOTHERAPIST 4: Hvad giver i jeres TKA'er? Tre måneder?

PHYSIOTHERAPIST 5: Nej, ikke nødvendigvis.

PHYSIOTHERAPIST 4: Nå, okay.

PHYSIOTHERAPIST 5: Det er meget forskelligt. Øh, vi, de for som udgangspunkt 12 gange. Så to gange om ugen ikke. Så de er der vel i en to måneders tid. Nogle gange så slutter vi dem før, hvis der er behov for det. Eller så forlænger vi dem bare. Vi har heldigvis ikke nogen begrænsning for hvor længe vi må forlænge dem. Så hvis de er virkelig dårlige så beholder vi dem bare.

PHYSIOTHERAPIST 4: Ja, vi kan også altid forlænge dem. Men vi har, hvad er det en TKA for, 10 gange?

PHYSIOTHERAPIST 3: Ja.

PHYSIOTHERAPIST 5: Ja okay, men vi ville gerne have det på 16 faktisk. Fordi der er en del af dem der er lidt tunge, og hvor vi ikke føler dem…

PHYSIOTHERAPIST 4: Især knæene. Hofterne de for 8 gange.

PHYSIOTHERAPIST 5: De for 16 hos os tror jeg. Det er lidt spøjst, fordi de bliver hurtigere færdige.

PHYSIOTHERAPIST 6: Har vi ikke gået ned til…

PHYSIOTHERAPIST 5: Er vi gået ned til 12?

PHYSIOTHERAPIST 6: Vi kiggede på vores kvalitetstandarder. Også valgte vi og gå ned på 12.

PHYSIOTHERAPIST 5: Ja, vi har lige det nemlig, men…

PHYSIOTHERAPIST 6: Vi gik op for knæ og ned for hofter ikke.

PHYSIOTHERAPIST 5: Jeg tror ikke vi har fået det igennem endnu.

PHYSIOTHERAPIST 6: Nå.

PHYSIOTHERAPIST 5: Øh, men vi prøvede nemlig og lave det om, fordi vi synes også det skulle være omvendt. Det gav jo ikke nogen mening. Så det skulle gerne laves om. Jeg ved ikke om det er kommet endnu.

PHYSIOTHERAPIST 3: Her på det sidste har vi fået flere øh, uni-knæ, end vi har haft tidligere.

PHYSIOTHERAPIST 5: Nåå, ja. Dem har vi også fået nogle stykker af.

PHYSIOTHERAPIST 3: Jeg ved ikke om der var en speciel læge på operationsgangen der lige…

PHYSIOTHERAPIST 5: Der lige skulle afprøve det.

PHYSIOTHERAPIST 3: Ja. Der var lige sådan hvor man tænkte, nå.

PHYSIOTHERAPIST 5: Bilaterale TKA'er de er til gengæld, de er godt nok langsomme…

PHYSIOTHERAPIST 4: Der er ikke ret mange der tager den… Et halvt år det er…

Støj, for mange taler.

PHYSIOTHERAPIST 5: Jeg har lige fået en. Hun har fået 4-5 grader fleksion på, altså ekstra, på 2½ måned i begge knæ. Der sker jo intet.

**Pausen er slut.**

RSH: Ja, nu har vi jo et kvarters tid tilbage. Der er lige et opfølgningsspørgsmål, også er der en sidste hovedoverskrift. Øh, så jeg starter lige med at følge op på en af de her, omkring det med og bruge teknologi i genoptræningen eller i træning. Og det går lidt på, om det her med at man har en øh teknologi med, som hvor man kan sige, det set-up vi har lige her, der teknologien den er ligesom en erstatning for jer på et eller andet niveau. Øh, og hvordan I føler at, tror I det er på linje med patientens værdier? Hvis I kan følge det. Den monitorerer, jo i stedet for at I monitorerer. Hvordan tror I patienterne har det med det?

PHYSIOTHERAPIST 6: Jeg kunne godt forestille mig, at der er nogle ældre der ikke ville synes så godt om det, også er der nogle af de yngre, som måske gerne vil omfavne det teknologisk lidt mere.

Og være mere selvhjulpne ikke, altså, ikke og kunne gøre ting selv. Og være mere selvstændige, øhm, men det afhænger rigtig meget af den enkelte, ikke. Og deres forståelse, øhm, så det er meget individuelt igen. Øh, men jeg tror at vi vil se de næste 10-20 år, at der vil komme flere der vil blive måske mere glade for det, altså. Øh, og varetage det selv i form af at de for noget teknologi der ligesom kan, at de kan bruge ikke. Hvad enten det er iPads, eller iPhones eller hvad ved jeg ikke.

PHYSIOTHERAPIST 5: Vi har også IKURA ude hos os, og der er det også dem der er lidt yngre, som for tilbudt det, sådan primært. Det plejer at være dem der er mere modtagelige for det i hvert fald. Det er også et rimeligt kompliceret system.

PHYSIOTHERAPIST 6: Der er mange af de gamle, da afskriver det helt. ”Nej nej nej nej nej nej, det er slet ikke noget”.

RSH: Er det sådan en teknologibarrierer, eller en vidensbarriere i virkeligheden der måske ligger der?

PHYSIOTHERAPIST 6: Ja.

RSH: Men hvad med det med at den, du var selv også lidt inde på det der xx, med at den… Vil de hellere have… Kan de acceptere det i stedet for at der står en fysioterapeut, som monitorerer og siger at det er godt eller dårligt? Og der er en eller anden teknologisk dims der ligesom, som måler dem også ender ud med et resultat, som så fortæller det at det har været godt eller dårligt.

PHYSIOTHERAPIST 1: Altså, jeg vil helst ikke sammenlignes med en BandCizer. Nej, jeg synes jeg kan lidt mere og noget andet end den kan. Ikke, for den kan bare tælle. Og det synes jeg i hvert fald… Også i forhold til IKURA, som vi nu kører ikke. Altså, den kan også bare… Det er noget teknologi, der bare kan tælle. Øh, også kan det også rette, men det kan ikke være pædagogisk. Øhm, og det kan ikke se at nu fungerer øvelsen helt forkert. Altså, den har jo bare de kriterier og køre ud fra, som den nu har. Der er altså nogle grænser, så jeg kan ikke sammenligne mig selv med den.

RSH: Men hvad tror du patienterne tænker om det?

PHYSIOTHERAPIST 1: Jamen nogle synes det er helt fint, og andre bliver så irriteret over at den retter dem flere gange. Fordi den er sat til at rette efter, måske man har lavet øvelsen, som den ser den forkert efter tre gange. Også er der bare noget som, altså hvor systemet så ikke er fuldstændig… Altså, maskinen er ikke fuldstændig tilpasset øvelsen, så de bliver rettet flere gange end de egentlig skal. Altså, der ligger nogle fejlkilder i det også ikke, også ”når nu siger den igen at jeg laver det forkert, men jeg gør jo som jeg skal”, og, altså på den måde.

RSH: Mmmm.

PHYSIOTHERAPIST 1: Men altså, jeg tror jo bedre teknologien bliver, jo bedre bliver det, men det kan aldrig… Jeg synes ikke teknologien kan erstatte mig som fysioterapeut. Med de øjne jeg har, og altså mødet med mig som person. Der ligger mange andre ting i det også.

RSH: Ja. Er det noget I andre kan nikke genkendende til?

Alle: Ja.

PHYSIOTHERAPIST 4: Det lyder i hvert fald til… Nu har vi ikke IKURA hos os, vi har haft nogle ude for fortælle om IKURA, men øh…

PHYSIOTHERAPIST 3: Vi er så småt i gang (med IKURA)… Men jeg tænker, hvis det var mig så tror jeg at hvis jeg havde øh… Hvis jeg var på arbejdsmarkedet, så ville jeg synes det var rart og få den teknologiske fordi man ikke kan få fri fra arbejde og sådan noget ikke også. Og det kan det være en lettelse for nogle, hvis man kan fornemme på patienten at det er en lettelse, at vi bare kan snakke over telefonen, så er det godt. Men hvis man har en af de der kroniske smertepatienter, som alligevel er sygemeldt og som ville have rigtig meget… Som gerne vil bruge terapeuten på ligesom og læsse noget over på, så må det være enormt frustrerende og skulle snakke ind i en telefon. Altså, hvis ensomheden er ved og…

PHYSIOTHERAPIST 1: Jeg synes også… Altså, men går jo glip af den der gruppedynamik som der jo også er. Og det med og møde andre, som er i gennem det samme som en selv. Og altså, man kan spejle sig i nogle andre også. Altså, det mister man jo hvis at man ikke kommer ind. Øhm, så det er bare noget andet, som måske også kan give et resultat, men… Ja… Jeg synes, jeg har det sådan at det er godt, men det er heller ikke kun godt.

PHYSIOTHERAPIST 2: Og det er slet ikke godt til alle.

PHYSIOTHERAPIST 1: Ja, og det er ikke sådan at man bare kan sige, at det passer til alle. Det passer til nogle, ja. Og det passer til dem der måske er kede af at skulle gå fra arbejdet, eller som gerne vil være i sommerhuset og på den måde ikke lige kan komme ind. Og hvor vi så også kan se, at det ser fornuftigt ud, du vil godt kunne træne med det her. Øhm, men det passer bestemt ikke til alle, og det er det jeg synes bliver lidt vanskeligt når kommunen måske siger, nå men det skal passe til alle.

PHYSIOTHERAPIST 2: Eller det skal passe til så og så mange. Altså, det er mindre og mindre vores egen vurdering, der ligesom kan spille ind.

PHYSIOTHERAPIST 1: Også begynder det og pille lidt ved vores etik også. Hvad kan jeg selv være med til som person? Jeg har… Vi har nogle måltal om at der skal være så og så mange med, men jeg synes faktisk at den ikke passer til fru Jensen. Jeg ville, ud fra min fysioterapi-viden, ville jeg hellere have at hun skulle komme på et traditionelt hold. Men jeg har jo også en chef, der står og prikker mig på skulderen og siger, ”nu er der ikke nok med i den gruppe”. Og der bliver jeg nød til og være, synes jeg, patientens advokat i det her. Så vidt muligt.

RSH: Så det, det er selvfølgelig et tænkt eksempel, men det kan skabe noget konflikt i virkeligheden?

PHYSIOTHERAPIST 2: Ja.

RSH: Er det noget I ser for jer?

Alle: Ja.

PHYSIOTHERAPIST 2: Ja, og ens egen rolle. Er det så sjovt og være fysioterapeut egentlig? Hvis det skal være på den måde.

Alle: Ja.

PHYSIOTHERAPIST 1: Skal jeg så være it-mand fremover? Ikke, også sidde og kigge på alle de her ting. Eller skal jeg være den der abe, der står og siger du skal lave det der? Den øvelse og den…

PHYSIOTHERAPIST 2: ”Så kigger jeg på computeren og tjekke om…”

RSH: Det er ikke et scenarie I synes godt om?

PHYSIOTHERAPIST 2: Nej.

PHYSIOTHERAPIST 1: Altså, som sagt til nogle kan det passe, og til andre passer det ikke.

RSH: Men jeg tænkte også jeres rolle. Hvis nu det var en udvikling.

PHYSIOTHERAPIST 1: Ja. Nej, jeg ville ikke synes det var så sjovt og være fysioterapeut så.

PHYSIOTHERAPIST 6: Men man kan jo også sige, det skal jo bare give mening jo. Det er jo ligesom, øh, vi havde et eksempel for kort tid siden hvor man har fået nogle iPads ude på vores arbejde. Hvor at det bliver, det er en pulje iPads, som er kommet til overs. Så skal de prøve og implementeres, men det er jo ikke fordi nødvendigvis at der er et mål med det, eller at man kan se at der er noget arbejde der bliver nemmere ved det. Det er fordi, at der er nogle der sidder og har en ide om, at det skal man gøre fordi, at så'n, det er den vej det går. Men hvis ikke man har et højere mål med, at det skal gøre dét her tidsbesparende. Øh, men det, vi skal bare prøve og hægte nogle op på et projekt.

PHYSIOTHERAPIST 5: Det var faktisk et fejlkøb af de der.

PHYSIOTHERAPIST 5: Ja, som vi så er blevet læsset på. Også skal man prøve og køre det ind på hold, så borgerne eksempelvis kan bruge en iPad til og monitorere deres egen træning. Men man skal jo ikke gøre det, bare for og gøre det. Man skal gøre det, fordi man har en plan med… Fordi vi tænker at det skal gøre det mere nemt for den enkelte borger og varetage deres egen træning, og et eller andet. Man har et eller andet specifikt mål med det ikke. Men det skal ikke bare være, nu for I de her, også skal I ligesom prøve og finde en løsning på at de implementeres i holdtræningen. Hvad er målet? Jamen det er sådan lidt diffust.

PHYSIOTHERAPIST 2: ”Men de er meget smarte og I har dem” (haha).

PHYSIOTHERAPIST 5: Ja, men man skal bare være meget skarpe på, hvad det er man vil opnå med det, og hvad man… Med hvilke borgere. Man skal måske have sådan en kasse inklusion eksklusion meget skarpt på, hvem er det vi tænker det er brugbart til.

RSH: Vi springer lige hurtigt videre til det næste. Øh, lige før pausen der snakkede vi lidt inde på det her… Der er en læge og en fysioterapeut i hver sin sektor, og der er en patient der er fanget i midten. Så det går lidt på den her, med den her model som er illustreret her. Hvor at denne her den afviger fra normalen, det er jo at når de kommer ind her (peger), så er de garanteret en operation. Øh, de bliver re-evalueret, så det er det der med at beslutningen bliver taget på et bedre grundlag, i og med at de har prøvet noget træning. Øh, så jeg vil gerne høre hvad I tænker om den her model, hvor I jo ligesom er med i denne her proces. Om I har nogle tanker omkring det? At de bliver henvist til jer, også kommer tilbage til kirurgen.

PHYSIOTHERAPIST 3: Der er jo tryghed i det. Altså, at de ved at de kommer tilbage uanset hvordan det er gået.

RSH: Ja, det er patienten. Jeg tænkte mere på med jer, jeres rolle i den her.

PHYSIOTHERAPIST 5: For vi at vide om de skal opereres eller ej?

RSH: Øh, ja i sidste ende har jeg selvfølgelig de data.

PHYSIOTHERAPIST 5: Ja, okay.

PHYSIOTHERAPIST 2: Jeg ved ikke om det er svar på spørgsmålet, men jeg tænker at det er godt der bliver afprøvet noget, øh, inden beslutningen om operation bliver taget og alle de risici der følger med en operation.

RSH: Ja.

PHYSIOTHERAPIST 4: Jeg er også godt tilfreds med og være en del af den her rolle her. Ikke, inden man så bliver re-vurderet igen, ikke. Altså, det har jeg det helt fint med.

RSH: Kan du prøve og sige noget mere om det?

PHYSIOTHERAPIST 4: Øh, jamen og være den øh, at man ikke øh, at man ikke direkte efter første konsultation siger, "jamen du skal have et nyt knæ, eller du skal dit og dat". At man lige for, øh, nu afprøver vi det her træning, og ellers bliver re-vurderet igen. Altså, det her træning det virkede ikke for mig, eller det virkede for mig ikke. Og det øh… Jeg synes det er et meget fornuftigt forløb det der.

PHYSIOTHERAPIST 3: Det er jo deres tryghed der gør, at der er rart for os. Ikke, altså, det er rart for at vide at patienten føler sig tryg, fordi han kommer tilbage uanset hvad.

RSH: Ja. Hvordan… Hvad med den her… Der er jo blevet taget en beslutning her (peger) også bliver der taget en beslutning når de så kommer tilbage igen. Øh, føler I med det her set-up at I bidrager mere til det, ift. det her med, vi snakkede om den her kommunikation med GOP'er hvor der måske ikke helt bliver talt samme sprog.

PHYSIOTHERAPIST 6: Gider du lige sige spørgsmålet igen?

RSH: Ja. Det er om I føler I bidrager mere til den beslutning der ender med at blive taget her (peger)?

PHYSIOTHERAPIST 1: Måske et bedre udgangspunkt for at tage den beslutning. Men nu er det jo stadigvæk et projekt. Så vi ved jo heller ikke om det lige præcis er den rigtige øvelse de bliver sat til ift. og evaluere om de skal have (TKA)... Men det er jo en del af projektet tænker jeg.

RSH: Ja, men hvis vi tænker uden om øvelsen her, men mere det her set-up (modellen).

PHYSIOTHERAPIST 1: Ja. Jeg synes da det er godt, og prøve noget ikke-kirurgi før at man skærer.

PHYSIOTHERAPIST 2: Men om det i princippet kunne foregå her på hospitalet at de blev instrueret eller om de kommer ud hos os, det ved jeg ikke om gør den store forskel. Men at de positive det ligger i at der er et tilbud inden at beslutningen bliver taget.

RSH: Ja, så øh, principielt er det lige meget om det gik over sektorer, eller blot det at der sker en fysioterapeutisk intervention?

PHYSIOTHERAPIST 2: At der sker noget andet. Ja, det tror jeg egentlig, men, det er det jeg tænker, men jeg ved det ikke.

RSH: Hvordan ser I andre på det? Er det det samme?

PHYSIOTHERAPIST 4: Ja, sådan har jeg det også. Fordi vi ser jo også folk som øh, hvor operation ikke var det store, ikke var den store succes. Og som skal i brisement eller som skal, eller som bliver ved med og hæve, eller som så går der kage i den der operation. Og det synes jeg, det er vigtigt med træning inden. Afprøve træning inden en øh, operation.

PHYSIOTHERAPIST 2: Mmmm. Jeg tænker så fordele ved at det foregår ude i kommunen er måske også, hvis man kigger på det lidt i det lidt større perspektiv. Det der med hvis de skal fortsætte med noget træning, også efterfølgende. Om det så er fordi de har undgået operationen eller post-operativt, så er de måske mere ude i noget der ligner et civilsamfund, end at det hele det foregår på hospitalet. Så det kunne der måske være nogle fordele med.

RSH: Ja, prøv og sig noget mere om det.

PHYSIOTHERAPIST 2: I forhold til at det ikke er så øh… Hvis alt foregik herude på hospitalet ville det måske være endnu sværere og komme herfra også fortsætte med noget træning ude i et lokalt center eller i nogle af de andre lokaltilbud. Hvor det måske giver noget andet, så og komme hen på et træningscenter, som ikke er så, jeg tænker det i hvert fald, så institutionspræget som et hospital er. Øh, og vi har nogle andre, øhm, kendskab til de tilbud som der findes i området. Det tror jeg kunne være en fordel, eller det håber jeg da har en, noget at sige. Om ikke lige i det her projekt så måske senere hen.

RSH: Ja. Og hvad med altså, hvad med når, hvad siger vedkommende så når de bliver henvist til enten noget mere træning eller prøver en operation og kommer tilbage til jer. Tror du så at den kommunikation der ville komme ud jer, ville være sådan lidt mere på, I ville være mere på samme linje eller på samme niveau i kommunikationen?

PHYSIOTHERAPIST 2: Altså ift. det vi for og vide fra kirurgen?

RSH: Ja, ift. hvordan det er nu, hvor det kan være lidt uklart sådan som jeg forstår det. Øh, om det så vil være… I og med at der har været en proces her, hvor der har været noget kommunikation mellem jer og en kirurg også kommer der kommunikation igen. At den på et eller andet niveau er oprettet.

PHYSIOTHERAPIST 6: Ja, også for os, men os, tror jeg, for den enkelte borger, at de vil føle at der er blevet, altså der er blevet taget godt hånd om dem. At de øh, at alle muligheder er afprøvet, og at man, at man har fået et rigtig godt forløb. Man har fået det bedste tilbud, men at alle muligheder er ligesom afprøvet, og derfor ender det enten med operation, eller det gik bare skide godt uden operation. At øh, de må immervæk føle at de for et bedre tilbud, tænker jeg, end sån de bare… Også rent kommunikativt, at de kan føle, at vi er ligesom med på klumpen og øh, de er med på klumpen, inden på hospitalet. Det kan godt føles sådan mere gennemsigtigt forløb tror jeg. Kunne jeg forestille mig.

RSH: Ja, er der… Kunne der være nogle ulemper ved det her? Kan I se nogle af dem?

PHYSIOTHERAPIST 2: De (patienterne) kunne godt tænke at vi var nogle idioter eller et eller andet, eller sådan. Nu har vi jo skulle instruere dem i én eneste øvelse. Hvis den ikke rigtigt har virket. Øhm, at de måske bare har fået endnu mere ondt, også skal de ud til os igen og træne. Så kunne det jo godt være at de er lidt forudindtagede. For mig, tænker jeg, så nu har jeg, i og med at jeg har set dem, måske en lidt bedre forståelse eller indsigt i hvad der er foregået forud for en eventuel operation. Det kan godt være at det har noget at sige, hvis de så kommer tilbage.

PHYSIOTHERAPIST 5: Ja, hvis de synes det var et dårligt forløb og de så ikke tager imod tilbuddet efterfølgende eller et eller andet.

RSH: Nåå, ja ja. Så de simpelthen siger nej tak?

PHYSIOTHERAPIST 5: Ja.

RSH: Okay, så er det det sidste spørgsmål. Det er sådan lidt med hvordan det her (modellen), hvordan I ser det kan passe ind i jeres daglige hverdag sådan praktisk som den er nu. Øh, for nu kører det jo på forsøgsbasis, men om det er… Hvordan I ser det kan passe ind?

PHYSIOTHERAPIST 1: Jeg synes det synes det passer fint ind, så længe der følger penge med i spillet ikke. Så er det jo fint.

RSH: Så længe der er penge, så er der også tid?

PHYSIOTHERAPIST 1: Jamen sådan er det jo.

PHYSIOTHERAPIST 2: Og nu er du der til at vi kan skrive til dig. Og du besvarer hurtigt mails osv., det er jo noget andet når vi skriver til kirurgerne, eller prøver og fange nogle herude (hospitalet), så går der langt mere ventetid imellem før vi for svar på vores spørgsmål. Så det kræver at der er en… At kommunikationsvejene er korte og ikke er så omstændige. Ehm, så hvis det ligesom var en del af hele samarbejdet med hospitalet, som det er i lige det her projekt, så ville det gøre nogle ting nemmere. Fordi vi har jo mail-adresser og kan få fat på de forskellige hospitaler osv., men det er ikke så simpelt som bare at skrive en mail altid.

RSH: Nej. Så det ville være en stor del af at det skulle kunne fungerer? Det er at der skulle være en så nem kommunikation som der er I vores tilfælde.

PHYSIOTHERAPIST 2: Ja.

PHYSIOTHERAPIST 6: Det at man har en tæt kontakt. Ja, det er også det der gør forskellen at man kan øh… Hvis det er lige er et problem, at man hurtigt lige kan høre dig eller…

RSH: Ja. Hvad hvis nu at øh, det ikke var mig. Hvis jeg havde en masse andet og lave, og der gik for lang tid. Hvad ville det så betyde? Sådan at I ikke fik svar på mailsne lige med det samme.

PHYSIOTHERAPIST 2: Ja, igen så bliver det hele lidt mere omstændigt. Trækker lidt for langt ud.

PHYSIOTHERAPIST 5: Ja, også hvis borgeren selvfølgelig føler at de for svar på det. Sån hvis de har nogle spørgsmål vi ikke kan komme med svar på. Og de måske også synes at det er et lidt dårligere forløb.

PHYSIOTHERAPIST 2: Nu har du også svaret hurtigt. Nu har jeg måske skrevet til dig lige inden jeg har skullet møde en eller anden patient. Og i forhold til og kunne, kunne måske besvare nogle af deres spørgsmål. Hvis jeg så selv ikke havde noget og få svaret, og sad i situationen med en patient og sagde, ”ved du hvad det bliver jeg lige nød til og følge op på, og ringe til dig senere, og fortælle dig hvordan det er du skal forholde dig”. Så vil det bare blive for, ja, usammenhængende. Ja, det tror jeg. Eller der tror jeg vi hjælper det på vej i hvert fald, ved at stille de bedst mulige betingelser om at kunne svarer på spørgsmål med det samme.

RSH: Ja.

PHYSIOTHERAPIST 1: Altså, umiddelbart så virker det jo meget enkelt synes jeg det her system. Og jeg kan ikke egentlig ikke se at der skulle være så mange problemer i det. Øhm, men selvfølgelig, jeg kunne se hvis det nu var den patient der kommer ud… De bliver jo instrueret første gang hos os, øhm, og hvis vi så fandt ud af, at det her det går bare overhovedet ikke. Det kan slet ikke lade sig gøre. Det er for mange smerter, eller hvad ved jeg. Også er der jo også lang tid de skal gå og vente før de kommer ind og snakke med en kirurg igen. Så går der de der 12 uger. Så kunne det være sådan noget med, at de måske skulle, ligesom tages ind igen tidligt. Men ellers så tænker jeg egentlig, at det er meget simpelt.

RSH: Ja, er der nogle flere kommentarer til det her? Nej. Så er der et enkelt punkt der hedder ”andet”. Hvis I skulle have et eller andet, som vi ikke har været inde på, som I godt kunne tænke jer at vi lige snakkede om.

PHYSIOTHERAPIST 2: Nej, men det er måske lidt mere i tråd med det xx lige spurgte om. Hvis vi så ser en der er alt for smerteplaget til at vi kan give en ordentlig instruktion, og tænke at det er, at det kan lade sig gøre. Hvad vi, om vi så, ja hvad vi så gør? Jeg ved ikke om det overhovedet bliver aktuelt, men…

RSH: Nej, øh, ja det er jo et spørgsmål til mig. Øh, jamen altså, min umiddelbare fornemmelse det vil være at vedkommende ikke er, ikke kommer med i projektet så. Fordi der vil være, altså så er de så meget en kandidat til kirurgi, at kirurgen vurderer at det er den bedste løsning.

PHYSIOTHERAPIST 1: Men der kan også være etniske borgere, altså etniske kvinder specielt, som har, altså giver meget udtryk for smerte. Altså, som det kan være rigtigt svært at vurdere hvor meget gør det ondt nu. Og er det… Altså, det synes jeg i hvert fald er et problem i dagligdagen nogle gange. At de siger ”meget meget ondt” ikke, også hvis skal score på 0-10, så er de oppe i 10 eller 20 ikke. Altså, fordi at de bare synes at det gør så ondt. Og det, ja det kan være måske lidt mere vanskeligt og finde ud af.

RSH: Ja, men ift. projektet så… Jamen den umiddelbare snak vi har haft om det, det er at dem der har sådan ekstremt dårligt, øh, de… Der bliver måske taget lidt anderledes hånd om dem. For det kan være at de har så svære knogledeformiteter, at der kan man sige at det kan man ikke træne væk, fordi knoglen er helt ude af form.

RSH: Ja, jamen så vil jeg da sige tak. Det var, øh, det var godt.

Alle: Selv tak.

RSH: Så er ideen jo, at vi følger op på det her når projektet er slut, hvis I har lyst. Og der går jo så noget tid ikke, kan I godt fornemme i hvert fald i xx og xx. Forhåbentligt så, hvad hedder han, xx, den ene kirurg har ambulatorie i dag, så måske ligger der nogle i indbakken når jeg kommer tilbage herfra. Men altså, de er i gang. Jeg venter bare på og få sendt nogle til her.

PHYSIOTHERAPIST 2: Og hvor mange er det der sådan skal kunne inkluderes i hele projektet?

RSH: 140, ca. 47 til hver kommune.

PHYSIOTHERAPIST 1: Udover piloterne?

RSH: Ja, udover piloterne. Så vi mangler 139 nu.

PHYSIOTHERAPIST 2: Og hvor længe regner i med, hvor lang tid tror I det tager og få alle dem?

RSH: Øh, vi har estimeret med 1½ til 2 år. Og jeg tror vi kommer op på 5 kirurger der inkluderer, når alle ligesom er sat i gang. Men lige nu er der kun været en i gang, for lige at få en fornemmelse af hvordan det kører, også er der to der blev startet i sidste uge og den ene var der så ikke, og den

anden fandt ikke nogle. Og det er en opgave der ligger på mine skuldre og være meget mere, altså sådan minde dem om det. Øh, for de har jo masser at se til. Øh, og der er også en række andre projekter de sådan skal have i mente. Så jeg har nogle udfordringer med at få adgang til noget data, sådan så jeg kan ligesom lave sådan en kode ud for hver patient, der betyder ”er potentiel deltager til det her projekt”. Også skal jeg, altså sådan helt simpelt bare vise mit ansigt og nævne det overfor dem.
