# Supplementary material for: Perceived facilitators and barriers among physical therapists and orthopedic surgeons to pre-operative home-based exercise with one exercise-only in patients eligible for knee replacement: A qualitative interview study nested in the QUADX-1 trial
Source: PLoS One. 2020 Oct 23;15(10):e0241175. doi: 10.1371/journal.pone.0241175 (PMC7584251; doi:10.1371/journal.pone.0241175)
Supplement: S8 File — (PDF) [file pone.0241175.s008.pdf]

Anonymous transcribed single interviews with orthopedic surgeons (in original language; Danish)

ORTHOPEDIC SURGEON 1

RSH: Sådan så er den i hvert fald i gang. Øh, ja, også har vi jo... Tanken er sådan ca. tre kvarter, det er det der er blevet sat af. Har du nogle spørgsmål inden vi sådan går igang?

ORTHOPEDIC SURGEON 1: Nej.

RSH: Så tager vi den bare løbende.

ORTHOPEDIC SURGEON 1: Hvad spørger du om?

RSH: Ja...

ORTHOPEDIC SURGEON 1: Det finder vi ud af?

RSH: Ja, jamen jeg kan ligesom tage overskrifterne. Så der er ligesom noget der er øh, det det, det er et område der handler om det her med at koordinere konservativ og kirurgisk behandling før operationen. Så er der lidt omkring den rolle i som ortopædkirurger har i denne her model, med også ligesom visitere til træning, hos dem I i virkeligheden kunne sætte kniven i med det samme. Så er der det med, at når de så kommer tilbage, med revurdering på baggrund af et forhåbenligt bedre beslutningsgrundlag, hvor du ser nogle af de ting. Også er der lidt ind omkring hvad det er det forhåbenligt giver at deltage for patienten og for jer. Der er lidt omkring self-managment altså de ligesom, de for nogle redskaber til måske selv og kontrollere deres sygdom, som måske i virkeligheden fjerner dem fra jeres operationsstue, hvis det virker. Øh, også er der det her med, at hvis de bliver opereret, om det så giver en bedre rehabilitering eller ændrer rehabiliteringen. Så det er ligesom overskrifterne.

ORTHOPEDIC SURGEON 1: Ja.

RSH: Øh, det første omkring det her med koordineringen. Og når jeg refererer til modellen, så er det det heroppe (peger på papir), den her ting vi prøver af. Så det første spørgsmål det går på omkring, hvad dine tanker er omkring at kandidater til TKA tilbydes konservativ behandling i form af træning inden en evt. opreration? Hvad dine umiddelbare tanker er omkring det?

ORTHOPEDIC SURGEON 1: Det står i lovgivningen at vi skal. Så, ej, det skal de også. Man kan sige det er øh, det er et gratis forsøg. Jeg ved ikke om det virker, det tror jeg ikke der er nogle der gør, men det giver god mening, fordi det er for tiden godt givet ud for langt de fleste. Så er der nogle individuelle kandidater der formegentlig ikke er egnet til genoptræning, hvor man bør opererer med det samme. Men det er fåtal.

RSH: Hvad mener du med at tiden er godt givet ud?

ORTHOPEDIC SURGEON 1: Det vil være godt givet ud, altså hvis du skyder operationen med tre måneder for at afprøve et genoptræningsforløb, så kan det godt være at det virker og det kan også godt være at det ikke virker. Men for patienten vil det være tre månender godt givet ud, for at afprøve en behandlingsmetode som måske ikke er super veldokumenteret, men er et forsøg værd, så at sige. Hvis man kan undgå operationen med tilhørende komplikationer.

RSH: Ja, så det er blandt andet omkring, altså nu sagde du komplikationer?

ORTHOPEDIC SURGEON 1: Ja.

RSH: Ja, præcis. Øh, så det er de umiddelbare fordele, det er at det næsten, det er sådan gratis. Kan man kalde det det?

ORTHOPEDIC SURGEON 1: Det er komplikationsgratis, altså på den måde, at det ikke koster ikke patienten noget at deltage i det (træning) fordi det er komplikationsfrit.

RSH: Hvad hvis man skal se, er der nogen ulemper du ser ved det?

ORTHOPEDIC SURGEON 1: Ved genoptræningen?

RHS: Ja, eller det her med at tilbyde dem træning også revurdering.

ORTHOPEDIC SURGEON 1: Man kan kigge på det samfundsøkonomiske i det. Det ved jeg så ikke om jeg har tallene for, men man kan diskutere, hvis man ved at et resultat efter kirurgi er bedre, dvs. at patienterne bliver løftet op på et højere niveau af kirurgi ift. træningen. Så kan det godt være at du har komplikationener ved kirurgien du ikke har ved træningen, men samfundsøkonomisk kan det godt være mere gavnligt at operere patienter, fordi du vil have en bedre succesrate på trods af at nogle komplikationer er der. Giver det mening?

RSH: Ja, det giver mening. Men også når man snakker sundhedsøkonomisk her, det er jo væsentlig dyrere at få en operation.

ORTHOPEDIC SURGEON 1: Ja, men det er jo ikke sikkert at det er det på længere sigt.

RSH: Nej, hvis du tager den helt lange bane, så tænker du stadigvæk at du kan være besparende, ok. Ja.

ORTHOPEDIC SURGEON 1: Fordi hvis du nu gør en patient rask med en operation, men operationen er dyrere, men genoptræningen det er ikke en kurativ behandling, det er vedholdelsestræning, så hvis du træner i tre måneder så kurerer du ikke din slidgigt, det er noget der skal vedligeholdes. Og det kan godt være at patienten kan træne sig selv, men hvis du ikke rykker patienten op på det samme niveau ved genoptræningen som du gør ved operationen, så i løbet af 10 år, så kan det godt være at operationen faktisk er en billigere løsning. Samlet set. Ikke for den enkelte patient som har fået en komplikation og har fået et elendigt resutat, men set over samlet. Jeg ved det ikke, men man skal opveje de her ting.

RSH: Nej, det er interessant fordi du har ret i, at de patienter der i virkeligheden... Hvis de oplever at træningen virker for dem, så når de har trænet i tre måneder, så er de ikke

færdige. Så skal de træne resten af livet. Øh, men her så træner de jo så faktisk usuperviseret. Så økonomien i det hvis de kan klare det hjemme derfra den er jo rigtig god, men det er rigtig nok hvis løftet (i funktion) ikke er tilstrækkeligt... Det er en god pointe.

ORTHOPEDIC SURGEON 1: Ja.

JWK: Hvornår vil I kunne sige noget om det?

ORTHOPEDIC SURGEON 1: Om økonomien?

JWK: I det her projekt...

ORTHOPEDIC SURGEON 1: Spørger du mig?

JWK: Jeg spørger jer begge to?

ORTHOPEDIC SURGEON 1: Formegentlig aldrig. Det tror jeg er en alt for kompliceret beregning til at man kan udtale sig om.

RSH: Ja, det er svært.

ORTHOPEDIC SURGEON 1: Der har været en god debat omkring det i Ugeskrift for Læger mellem Ewa Roos og Søren Skou. Og Krogsgaard (Michael). Altså det var meget sjovt fordi, at de havde begge to ret, men de kiggede på det fra meget forskellige ting. Og det er netop her de diskuterede med cost-benefit af genoptræning, om man kan sige det ene eller det andet, og det kan man ikke, så...

RSH: Nej, og der er i hvert fald en pointe i det her der hedder, superviseret/ikke-superviseret fysioterapi. For der er to, der er økonomien vidt forskellig ikke.

ORTHOPEDIC SURGEON 1: Præcis.

RSH: Så hvis man ligefrem, hvis det her virker med meget usuperviseret træning, så vil det jo være væsentlig billigere end GLAD-konceptet som er det Søren og dem arbejder med, som er meget superviseret ikke.

ORTHOPEDIC SURGEON 1: Ja.

RSH: Hvis vi så tager det her. Hvordan, sådan selve modellen her, og det du har oplevet nu i en måneds tid i dit ambulatorie, hvordan passer det ind i din dagligdag? I din daglige praksis?

ORTHOPEDIC SURGEON 1: Altså for mig er det jo fuldstændig ligegyldigt hvordan jeg dikterer at patienten skal henvises til kommunal genoptræning, gerne i GLAD-regi, eller at jeg henviser tilgenoptræning i det her regi. Fordi det er fuldstændig ligegyldigt. Fordelen ved det her (QUADX-1), det er der ved du hvad du for, fordi vi kan ikke henvise direkte til GLAD. Jeg synes næsten det er er nemmere, fordi ved GLAD skal man bruge relativ lang tid på at forklare patienterne hvad GLAD det er, og hvordan de kan opsøge det. Fordi man kan ikke henvise til GLAD. Og hvis det

ikke bliver GLAD-træning, så tror jeg at spændet mellem dårlig og god genoptræning er ret bredt. Her (QUADX-1) ved du præcis hvad du for. Logistisk set er det fuldstændigt...

RSH: Ja, så lidt i tråd med det. Øh, hvis man kigger på det lidt mere organisatorisk, hvem føler du der skal have ansvaret for at det her det kører?

ORTHOPEDIC SURGEON 1: Fysioterapeuten.

RSH: Hvis nu at jeg ikke var her, og det her det faktisk kørte (reelt offentligt tilbud), så ville du skulle sende henvisningen ud til kommunen. Så det ville være fyssen i kommunen, der havde ansvaret for at det fungerede?

ORTHOPEDIC SURGEON 1: Ja.

RSH: Okay, så i virkeligheden sådan som det fungerer nu med genoptræningsplaner osv. I dikterer følgende også skal den samles op derude?

ORTHOPEDIC SURGEON 1: Ja.

RSH: Okay, ja.

ORTHOPEDIC SURGEON 1: Det har ikke noget med os at gøre. Jeg tror i princippet ville vi nok foretrække, i en perfekt verden, at genoptræningen foregik hos os, hos vores fysioterapeuter fordi jeg tror, at de måske i gennemsnittet er bedre end dem ude i kommunnen uden at vide særlig meget om kommunen. Men man hører meget forskelligt, altså nogle har haft superfine genoptræningsforløb og nogle har fået massage i tre måneder, som sikkert er rart, men jeg tvivler på at det hjælper på deres slidgigt.

RSH: Det er klart.

ORTHOPEDIC SURGEON 1: Øhm, men i princippet er det den behandlende fysioterapeuts ansvar, om det så ligger ude i kommunen eller på hospitalet, der er ansvarlig for at forløbet bliver gennemført.

RSH: Okay. Øhm, jeg skal lige... Hvad var det jeg tænkte på der... Jo, det var det der med at have det i huset. Så hvis nu det foregik i fysioterapien hernede, det ville du se som en fordel? Fordi at du ville have mere kontrol med...

ORTHOPEDIC SURGEON 1: Nej, jeg ville på ingen måde have behov for at kontrollere. Jeg ville ikke blande mig i det overhovedet. I (fysser) ved hvad I laver og det er ikke... Hvis man nu sammenligner med skuldergenoptræning eller et eller andet. Så kan det godt være at du har en set-back hvor du gerne vil have, at lægen er indover og vurdere, skal vi gå mere til det eller mindre til det. Men slidgigt enten virker det, eller også virker det ikke. Så jeg tror, det er ikke fordi man som læge har behov for at være indvolveret, men jeg tror vores fysioterapeuter i huset vil være bedre til at varetage træningen end dem ude i kommunen, fordi at træningen vil være meget mere ensrettet.

RSH: Okay, ja.

ORTHOPEDIC SURGEON 1: Hvis genoptræningen ude i kommunen var ens over det hele, så ville det være fuldstændig ligegyldet om det lå ude i kommunen eller i huset.

RSH: Ja, okay.

ORTHOPEDIC SURGEON 1: Det er ikke fordi jeg har brug for, eller andre læger, har brug for at blande sig i det før forløbet er gennemført.

RSH: Nej, okay. Øh, lidt videre på det organisatoriske. Det er om det, om den model, og det er jo stadig meget ungt, og det er derfor vi laver interviewet nu, men føler du at det på nuværende tidspunkt er noget der vil kunne implementeres?

ORTHOPEDIC SURGEON 1: Ja ja, altså som sagt jeg synes, om du bruger modellen eller om du bruger almindelig genoptræning. Nu jeg ved ikke hvordan de andre kirurger de gør, så er det lidt mere svært, men jeg tror at de fleste af os tilbyder patienten i det mindste genoptræning, hvis de ikke har været igennem et genoptræningsforløb. Udover at patienterne enten siger at de ikke ønsker det, eller man konkluderer at de på ingen måde er egnede til at varetage et genoptræningforløb. Men hvis det er nogle der er kandidat til genoptræning, om du bruger den model eller bare henviser til kommunen er det i princippet for os fuldstændig det samme, fordi du ser at patienten skal til genoptræning medblik på knæstabiliserende styrketræning med fokus på quadricepsstyrke, ses til kontrol efter tre måneder. Det er jo præcis det samme som det her, så...

RSH: Ja, der hvor vi i hvert fald i teorien synes den her (modellen) den afviger lidt, det er at der er en standardiseret opfølgning tre måneder senere. Nogle gange bliver folk jo henvist, er mit indtryk, ud til kommunen uden måske at have et fast holdepunkt for hvornår de snakker med jer igen.

ORTHOPEDIC SURGEON 1: Ja, det tror jeg er meget kirurgindividuelt. Jeg plejer at se patienterne igen. Også fordi, så, medmindre at jeg 100% ved at patienten ikke er kandidat på operation, men så vil han heller ikke være kandidat i det her projekt. Man kan godt se en der har begyndende slidgigt eller slidgigtsymptomer, men det på forhånd har sagt, at selvom genoptræning ikke hjælper, så vil jeg stadigvæk ikke operere dig. Også giver det ikke noget mening at give kontrol, men så vil han heller ikke være egnet til det her. Men hvis du har en der er kandidat til kirurgi, så tror jeg de fleste vil af os (kirurger) vil følge denne her (model). Dvs. at se patienten efter endt fysioterapeutisk forløb.

RSH: Ja, okay. Øhhh, også er det sådan lidt mere generelt, måske lidt lavpraktisk. Om der er nogle ting du, faktorer som du synes vi skal være opmærksomme på for, at det her (projektet) det kører?

ORTHOPEDIC SURGEON 1: Compliance, men det giver sig selv.

RSH: Compliance, hos patienten? Ikke hos jer (kirurger)?

ORTHOPEDIC SURGEON 1: Ja, hos patienten.

RSH: Hehe, ja ok. Men det er jo så der...

ORTHOPEDIC SURGEON 1: Men der skal også være compliance hos, men det er... Men på den måde vil du lave et flowchart, det står i retningslinjerne at patienten skal være tilbudt konservativ behandling før kirurgi. Så hvis du ikke gør det, så kan man sige, så skal du argumentere i journalen. Jeg tror langt de fleste har skrevet, "har prøvet genoptræning uden effekt" eller "ønsker ikke genoptræning". En af de her to ting, eller "vurderes ikke egnet til genoptræning".

RSH: Ja.

ORTHOPEDIC SURGEON 1: Men det tror jeg ikke rigtig har indflydelse på logistisk. Fordi på det tidspunkt er skibet sejlet. Altså hvis vi nu har besluttet os for at operere patienten uden at sende ham til genoptræning, så er det jo for sent bagefter og sige, "du skulle have fået genoptræning".

RSH: Ja, det er rigtig. Det skal selvfølgelig fanges.

ORTHOPEDIC SURGEON 1: Man ville heller aldrig opdage det, så.

RSH: Men i virkeligheden så leder det her, det her med timing, det leder lidt hen til det næste område, som handler om jeres rolle som ortopædkirurger i det her. Øh, og lidt omkring hvilke barrierer der ligger hos kirurger for konservativ og operativ behandling. Øh, så hvilken rolle mener du, at du som kirurg har i det her... Med og koordinere konservativ og kirurgisk behandling?

ORTHOPEDIC SURGEON 1: Det er nok, at man skal... Altså for det første huske at tilbuddet er der, og man som udgangspunkt bøt benytte sig af det. Øhm, men måske også skarpere, hvis man havde nogle skarpere selektionskriterier på hvem er egnet til genoptræning, fordi man har nogle... Man kan godt have en egen ide om hvilken patient, ikke er egnet til genoptræning, men det er ikke evidensbaseret. Så hvis man nu har en, det ved jeg ikke, en 80-årig mand med et fuldstændigt smadret varus-knæ med bone-on-bone i alle tre kamre, der er i panodil-adolol behandling. Så nogle gange kan man tænke, "det er fandme åndsvagt at jeg henviser ham til genoptræning i tre måneder", fordi det vil være spild af tid. Altså, det bliver næppe bedre af det, men jeg har ikke noget evidensbaseret grundlag for at sige det. Jeg er ret sikker på at det ikke hjælper, men jeg tror ikke du kan vise en artikel, der viser sammenhæng med slidgigt og plus/minus effekt af genoptræning. Men det er nok det største indflydelse vi har. Det er netop at selektere patienterne. Man kan godt være fuldstændig blind og sige, alle skal til genoptræning, men det ville også være ressourcespild. Og så også for patienter, fordi nogle gange har du også nogle der siger, "jeg er ude af arbejdsmarkedet nu her, jeg bliver fyret hvis jeg ikke kommer tilbage på arbejdet inden for tre måneder". På samme måde så skal du overveje, skal han tilbydes genoptræning med måske 50% succesrate, eller skal man operere ham nu her med 80% succesrate.

RSH: Ja, så det er i virkeligheden... Der er også noget... Det er lidt blødere end at vi sætter to hårde kasser op hedder "konservativ" og "kirurgisk", men der er også individet indblandet i det i sidder sammen med i virkeligheden?

ORTHOPEDIC SURGEON 1: Jamen, det er der også klart. Det samme gælder for smertestillende, som parallel til det her. "Der står at patienten har prøvet smertestillende", men igen hvis du har den samme der har daglige belastningsrelaterede smerter, har spist ikke panodil, men siger at han har prøvet det engang uden at det havde nogen som helst effekt. Så kan du godt finde på at operere patienten uden at have givet ham panodil/ipren i tre måneder, fordi højst sandsynligt så kan det godt

være at du alligevel skal operere ham om tre måneder, også vil det være spild af tid. Eller han vil rende rundt med det (smerter) indtil da. Så for alle patienter vil det formegentlig være en individuel vurdering, men derfor skal vi stadigvæk huske på, at som udgangspunkt så skal man have været igennem et konservativt behandlingsforløb inden operationen. Med mindre andre forhold gør sig gældende.

JWK: Må jeg spørge om noget?

RSH: Ja, selvfølgelig.

JWK: Det er fordi, du sagde rent faktisk noget spændende, "man skal huske at tilbuddet er der". Jeg tænker, er der noget som RSH kan gøre fra projektets side af, for at I husker det?

ORTHOPEDIC SURGEON 1: Jeg tror at vi allesammen godt husker det.

JWK: Det gør I?

ORTHOPEDIC SURGEON 1: Ja.

JWK: Okay, så der er ikke noget der?

ORTHOPEDIC SURGEON 1: Nej. Ikke engang dit projekt. Det kan vi jo til gengæld godt finde på at glemme (uden for projekter?). Nu skriver du en seddel op, så vi på ingen måde er i tvivl. Men jeg tror ikke der er nogle kirurger, der på nogen måde glemmer at der er en konservativ genoptræning mulighed. Altså, det ligger helt indlejret.

JWK: Nej, jeg tænkte bare sådan specifikt. Det er nemlig nemt at glemme projekter. I er med i mange projekter tænker jeg. Så jeg tænker om der var noget man kunne gøre for RSH's?

ORTHOPEDIC SURGEON 1: Lige netop til det her projekt, så tror jeg ikke du kan gøre mere. Du gør rigeligt. Bliv ved med at gøre det, fordi så glemmer vi det ikke når der står "husk at inkluder til projektet".

JWK: Jeg tænkte også på de der selektionskriterier. Er det fordi de er, fra projekside af ikke skapt defineret, i forhold til...

ORTHOPEDIC SURGEON 1: Nej, de er relativt skarpt defineret til projektet. Hvad tænker du på?

JWK: Det er fordi du sagde, at der kan være noget med selektionskriterier, fordi det også bliver en individuel vurdering og en relationel vurdering ift. om man kommer ind og ud. Så tænker jeg igen, er der noget/nogle knapper man kan skrue på?

ORTHOPEDIC SURGEON 1: Nå, men vi kan sagtens gøre det i projektet. I projektet er det netop skarpere defineret end det er i virkeligheden. Fordi i projektet er det defineret at alle patienter, der er kandidater til operation, men har ikke prøvet genoptræning, skal tilbydes genoptræning. Det er ret skarpt. Det kommer du ikke uden om, men i det virkelige liv kan du godt have en patient, som ikke har været igennem genoptræningsforløb, som man stadig ikke mener skal igennem et genoptræningsforløb. Netop fordi at han har et mega smadret knæ. Eller en kørestolsbruger, hvor

det ikke giver mening at henvise. Men i følge projektet skulle vedkommende stadigvæk igennem. I det her tilfælde vil der nok ikke inkluderes og skrive at, da patienten alligevel ikke egnede sig til genoptræning For ikke at spilde din (RSH) tid, men...

JWK: Okay, det var bare for at jeg forstod, om der var noget man kunne skrue på for projektets side af. Om der var noget I manglede noget.

ORTHOPEDIC SURGEON 1: Nej, jeg synes det er ret klokkeklart.

RSH: Så i virkeligheden som jeg hører det her, så øh, som kirurger der sidder med en patient inden operation, så er din vurdering at I har, I vejer den operationelle kasse og den konservative lige tungt til at starte med når vedkommende træder ind af døren?

ORTHOPEDIC SURGEON 1: Hehe. Ej, det gør vi nok ikke. Ellers ville man ikke være kirurg. Øh, jeg tror langt de fleste af os, har en ide om hvilke patienter man gerne, man godt kan hjælpe og ikke kan hjælpe. Også er der en del patienter hvor man tænker, at din succesrate ved operationen vil være væsentligt lavere. Og dem vil man være væsentligt mere tilbøjelige til at skubbe over i genoptræningskassen. Men det bliver en meget subjektiv vurdering, så det er svært at pendsle det helt ud, men det er klart, hvis igen, hvis du har en patient med fuldstændig klokkeklare artrosesymptomer og klokkeklar radiologisk slidgigt. Så nogle gange vil en del kirurger, sikker også mig inklusiv, tænke "hvorfor skal vedkommende træne, når vi ved at såfremt en patient er sund og rask og ikke kommer til at have komplikationer, så vil vi kunne afhjælpe ham med god sandsynlighed og han kommer til at have et godt resultat". Men hvis du har en dame med BMI på 40 og fibromyalgi, og noget slidt knæ, men ikke rigtigt, der har et lidt mere broget symptombillede, men i princippet er kandidat til operation fordi hun har klinisk artrose, og der er noget på røntgenbilledet. Så vejer konservativ behandling formegentlig væsentlig mere, fordi det er en patient du vil være meget gladere for at skulle undgå og skulle operere.

RSH: Ja.

ORTHOPEDIC SURGEON 1: Giver det mening?

RSH: Ja, det giver rigtig god mening.

ORTHOPEDIC SURGEON 1: Så er det svært at sige hvad for en der vejer mest. Det er meget patientindividuelt, men jeg tror at vi vil betragte begge to som behandlingsmuligheder.

RSH: Ja, og det var lidt det spørgsmålet det i bund og grund gik på. Nu stilte jeg det lidt hård op, men øh...

ORTHOPEDIC SURGEON 1: Ja, og så kan man sige at den ene er mere evidensdokumenteret med evidens end den anden.

RSH: Ja. Nu har, vi har været lidt inde på det, men nu stiller jeg bare spørgsmålene igen, så er det mere præcist på det. Så det, hvilken patient ser du som en god kandidat til at gennemgå et træningsforløb inden operation?

ORTHOPEDIC SURGEON 1: Patienter som er motiveret for det, og som ikke har alt for meget

funktionel indskrækning. Fordi på et eller anden tidspunkt vil jeg umiddelbart gætte på, at det er for sent. Øhm, som har noget funktionelt niveau. Så i princippet er det nogle der er motiveret for det.

RSH: Okay. Hvad hvis de er øh, bare kommer ind og beder om en operation, men at du ikke synes at de er operationsklare?

ORTHOPEDIC SURGEON 1: Ja, så ville jeg sige nej.

RSH: ...og tilbyde dem?

ORTHOPEDIC SURGEON 1: Genoptræning.

RSH: Okay. Også er det op til dem selv?

ORTHOPEDIC SURGEON 1: Og hvis de så siger nej... Ja... Og smertestillende.

RSH: Ja, selvfølgelig. Også er der et (spørgsmål) omkring, om der er nogle patienter du mener der vil, øh måske have gavn af træning, men som du vælger at operere direkte alligevel?

ORTHOPEDIC SURGEON 1: Ja, men dem er der også nogle af. Men der er nogle patienter der sige, "jeg gider ikke og træne". Altså, "jeg har hørt om det, jeg ønsker ikke et genoptræningsforløb, jeg vil gerne blive opereret". Og du kan godt fælge hesten til vandet, men du kan ikke tvinge den til at drikke. Så kan det godt være, at jeg kan henvise patienten til genoptræning, og siger jeg insisterer på det. Men hvis han kommer tilbage tre måneder efter og siger, "jeg har ikke trænet, nu vil jeg gerne blive opereret", så er kriterierne opfyldt, men så har man igen spildt tre måneder, både hans og min tid, så...

RSH: Ja, så er de såkaldte "gratis" måneder de er i virkeligheden blevet spildt?

ORTHOPEDIC SURGEON 1: Ja. Altså, det er ikke fordi, at det på ville gøre noget. Hvis patienten på forhånd siger, "at jeg gider ikke træne jeg ønsker operation", og han er informeret omkring fordele og ulemper rent ud sagt.

RSH: Så i virkeligheden, så det næste der har jeg tænkt lidt ind omkring hvilke hindringer du ser på systematisk at bruge træning til behandlingen af knæartrosepatienter? Men er det i virkeligheden så lidt det med motivationen? Er det en af dem du oplever?

ORTHOPEDIC SURGEON 1: Ja. Det er motivation også kan der godt være enkelte faktorer. Det er jo en hindring for de her patienter hvor det ikke virker. Og det er kun et problem, hvis folk er i arbejde.

RSH: Men de er vel nød til at prøve det, for at de ved om det virker? Kan du følge mig? Det er hvis du sidder og skal tilbyde dem det...

ORTHOPEDIC SURGEON 1: Jaja, præcis men jeg tænker ikke, hvis du stiller det helt skrapt op, og patienten er i øh jobtruet og har brug for at komme tilbage på arbejdsmarkedet hurtigt nok, så spiller det alligevel en rolle for ham, om du vælger behandling med en 80-90% succesrate, som til

gengæld er sådan en "high risk, high reward" behandling. Eller at man udskyder hans operation med tre måneder med noget der måske/måske ikke virker med mindre succesrate. Til gengæld som er komplikationsfrit. Fordi hvis du er pensionist så vil du helt sikkert sige "ja, det vil jeg gerne fordi jeg er ligeglad". Men hvis du ved at du bliver fyret, så spiller arbejdet en større rolle, så jeg vil gerne blive opereret nu her.

RSH: Ja, godt. Har du noget mere her Jeanette?

JWK: Nej, det er meget interessant faktisk. Meget komplekst.

RSH: Ja. Går videre til en ny overskrift, som handler lidt omkring øh, at det bliver, når de kommer ind anden gang, så er vi herovre (peger på modellen), om beslutningen bliver taget på et bedre grundlag? Så der er en sætning her der lyder, "at formålet med projektet er at vurderingen af den bedste behandling til patienten er baseret på et så godt grundlag som muligt, dvs. at konservativ behandling er prøvet af før operationen". Tror du at dette vil give jer et bedre/bredere beslutningsgrundlag ift. om operation er den bedste behandling eller ej?

ORTHOPEDIC SURGEON 1: Ja, det vil det alt andet lige. Altså, for så har du flere informationer, også har du afprøvet... Jeg tror ikke det vil ændre på dit beslutningsgrundlag om patienten er kandidat til operation eller ej, for det har du besluttet uanset om han har været til genoptræningsforløb eller ej. Så hvis du har klinisk radiologisk symptomgivende slidgigt, så er det kandidat til operation uanset om genoptræning har været forsøgt eller ej. Så kan man godt fjerne de symptomer med genoptræning. Man kan skrive genoptræning som parallel til smertestillende. Og hvis det har haft effekt, så er patienten ikke kandidat til operation. Men i princippet har du jo truffet beslutningen om at patienten ville kunne blive opereret på forhånd.

RSH: Ja, men det er netop det om de så...

ORTHOPEDIC SURGEON 1: Du kan fjerne operationsindikationen, men du kan ikke tilføje det på det baggrund. Hvis det giver mening. Du kan godt gøre patienten rask ved genoptræning, men hvis du ikke synes han er kandidat til operation før genoptræningen, så tror jeg næppe han vil være kandidat til operation efter genoptræningen.

RSH: Nej, okay. Så det i virkeligheden, så er du med på præmissen om at du godt kan fjerne operationsindikationen på baggrund af... (træning)

ORTHOPEDIC SURGEON 1: Jajaja, ellers så ville du jo ikke sende dem til genoptræning. Så giver det jo ikke mening.

RSH: Nej, sådan kan man selvfølgelig sige det. Kunne der være nogle ulemper ved det? Altså, nu det var klare fordele det her, sådan som jeg ser dem i hvert fald.

ORTHOPEDIC SURGEON 1: Ved genoptræning?

RSH: Ja, at hvis nu at genoptræningen faktisk hjælper, så har du hjulpet patienten på en mindre invasiv, potentiel billigere måde afhængigt af hvordan man ser det. Kunne der være nogle ulemper set fra kirurgens synspunkt?

ORTHOPEDIC SURGEON 1: Hvis det ikke virker.

RSH: Ja, så virker, hvad hedder det øhm, fysioterapi eller genoptræning ikke, men så står de kirurgiske tilbud jo sådan set stadig ved magt.

ORTHOPEDIC SURGEON 1: Nå, jeg tror ikke det kommer til at påvirke resultatet, fordi på tre måneder så falder dit funktionsniveau næppe særlig meget. Så på den måde spiller det ikke den store rolle. Men så har du bare spildt igen tid og penge, at det ikke har virket. Med hjemmetræning spiller det ikke en stor rolle, men genoptræningen ude i kommunen er en relativ heftig udgift.

RSH: Ja.

ORTHOPEDIC SURGEON 1: Men ved hjemmetræning så er det kun hvad, en instruktion og opfølgning så er det ikke særlig omkostningsfuldt.

RSH: Nej.

ORTHOPEDIC SURGEON 1: Men rent funktionelt så tror jeg ikke det har nogle udgifter. Hvis der er nogle patienter der siger de har fået det værre af genoptræningen, men det havde de sikkert også fået hvis de havde vadet rundt på gaden i tre måneder og ventet på operationen.

RSH: Ja, det ved vi jo ikke.

ORTHOPEDIC SURGEON 1: Jeg tror ikke at det gør noget værre i hvert fald, under alle omstændigheder.

RSH: Nej, men i virkeligheden så øh, jo du var lidt inde på, at det kunne godt skabe et bredere beslutningsgrundlag, men i og med at de i virkeligheden har indikationen, så kommer det ikke måske til at rykke...

ORTHOPEDIC SURGEON 1: Det styrker din indikation, men det kommer ikke til at rykke det den anden vej.

RSH: Godt. Øh, så hvad tror du patienterne tænker om det her med og få tilbudt noget træning, også blive re-vurderet?

ORTHOPEDIC SURGEON 1: Det er mega individuelt.

RSH: Ja...

ORTHOPEDIC SURGEON 1: For/før(? 24.35) vi snakker med dem. Nogle synes, de fleste, jeg vil gætte på 75%, slag på tasken, vil gerne prøve konservativ træning, fordi de er tilbageholdene med operation pga. komplikationer og risici. Også er der også nogle der siger, at jeg vil ikke træne. Eller fordi de har prøvet en eller anden form for træning, som de ikke synes hjælper.

RSH: Ja. Øh, og hvad... Jeg skal lige finde på at formuere denne her. Jo, hvad karakteriserer dem, har du sådan et billede af dem, der er motiveret for det og dem der ikke er motiveret for det (træning)? Er der et eller andet simpelt der lige umiddelbart springer i hovedet?

ORTHOPEDIC SURGEON 1: Ikke umiddelbart. Altså, de fleste der ikke er motiveret for det, det er folk i arbejde (men som er sygemeldte?) som har brug for at komme tilbage til arbejder, som har brug for en lidt hurtigere løsning. Og som har prøvet en eller anden form for træning før, som ikke er decideret genoptræning. Fordi man kan godt sige, som person der dyrker en del motion, du cykler noget. Nu ved jeg godt vi snakker om quadricepsstyrkende øvelser som udgangspunkt, men hvis patienten siger han går i fitness center og træner benpres med videre og cykler, og det har ikke haft noget som helst effekt, så er de relativt skeptiske for at blive henvist til tre måneders yderligere genoptræning af quadricepsmuskulaturen.

RSH: Ja, så dem der allerede er ret aktive de tænker "kan det hjælpe mig overhovedet der her?"

ORTHOPEDIC SURGEON 1: Præcis.

RSH: Hvad med sådan noget med vægt. Er der et billede af at dem der vejer mere, som jo også er en ting vi ved lidt om påvirker symptomer deres symptomer, at de er mindre motiveret eller mere motiveret?

ORTHOPEDIC SURGEON 1: Det ved jeg ikke. Det tror jeg ikke jeg har noget belæg for.

RSH: Nej.

ORTHOPEDIC SURGEON 1: Min erfaring er, at dem der vejer en del, ikke er særligt motiverede for vægttab. Jeg tror ikke jeg kan huske en eneste patient der har sagt, "jamen, jeg vil gerne prøve og tabe mig". Alle sammen siger, "jamen jeg kan ikke tabe mig, fordi jeg ikke kan gå". Det er så noget vrøvl, men altså... Ja...

RSH: Hehe, ja.

ORTHOPEDIC SURGEON 1: Og så kan man nogle gange sparke til dem med højrebenet, også håbe på at de taber sig alligevel i forbindelse med genoptræningen, men det er næppe der problemet ligger henne så, nej jeg har ikke...

RSH: Nej, det var bare om der var en karakteristika der, fordi der er jo...

ORTHOPEDIC SURGEON 1: Måske, men igen jeg har ikke noget belæg for at sige det. Så bliver det ren empiri og min egen holdning.

RSH: Jaja, men det er også den jeg gerne vil have.

ORTHOPEDIC SURGEON 1: Okay, jaja, men så er det nok formegentlig fordi folk med høj vægtklasse har dårligere funktionsniveau, også kan de dårligere være med til genoptræning. Fordi der er også nogle der siger, "jeg kan ikke være med til genoptræningen fordi jeg har så ondt, og ikke kan bruge mit ben". Som regel er det overvægtige.

RSH: Så i virkeligheden er der lidt en barriere der for nogle af dem?

ORTHOPEDIC SURGEON 1: Måske, igen, men jeg har ikke noget belæg for at sige det, så...

RSH: Okay. Har du noget Jeanette?

JWK: Nej.

RSH: Så er der et nyt punkt, og det er under den overskrift jeg har kaldt "self-management". Så det er når patienterne gennemgår denne her konservative behandling, så modtager de også redskaber. Altså de modtager en, i det her projekt, en træningsform som de i virkeligheden kan klare derhjemme selv. Og de for også lidt læring i smertehåndtering, i hvert fald nogle retningslinjer for hvordan de skal gøre det, øh ift. deres knæ. Øh, og det kan jo f.eks. være at de oplever, at hvis de træner eller hvis de håndterer deres knæsmerter på en anden måde, så bliver det nemmere for dem i hverdagen. Mit spørgsmål går på hvad du tænker omkring det her med, at patienterne bliver så at sige uddannet til selv bedre at håndtere deres sygdom.

ORTHOPEDIC SURGEON 1: Det er jo fint. Øh igen, mig bekendt findes der ikke nogle undersøgelse der viser, at træning med en fysioterapeut er bedre end selv-træning instrueret. Hvorimod der findes ret mange undersøgelser der viser, at de er fuldstændig ligeværdige, så på den måde har man fjernet en udgift igen. Også kan du måske selektere patienter der har brug specialiseret genoptræning. Mens artosesmerter, I må rette mig, men alt hvad jeg har læst omkring det, det viser at selv-træning er lige så godt som superviseret træning. Så på den måde er det fint, også er det sikkert godt at patienten tager ansvar for din egen træning, hvis det er det du mener? At han selv bliver i stand til at håndtere sin egen sygdom, men det bliver meget individuelt. Nogle vil være gode til det, og andre har det bedst at "det her er mit problem, løs det". Også er de ikke helt så imødekommende overfor forslaget omkring, at du skal selv løse det ved at træne. Så jeg tror ikke det er alle der er glade for den løsning, eller at det er den optimale øvelse for alle. Men det er klart en god løsning for en del.

RSH: Ja, så som kirurg tænker du, at hvis det var et, at du også kan sælge denne her ide til dem? Ser du det som realistisk at du tænker nu, det her det er i virkeligheden et tilbud til dig, hvor du for nogle redskaber, som du selv kan bruge sådan sådan at du slipper for fysioterapeuten, kirurgen, lægen.

ORTHOPEDIC SURGEON 1: Det er svært, fordi generel patientkompliance tror jeg er relativt ringe på alle områder. Så ulempen ved det her er netop, at du skal sikre at patienten træner selv. Og hvordan man gør det, det ved jeg ikke. Jeg tror godt det kan sælges til patienten, fordi nu kan jeg huske at der er nogle stykker faktisk der har spurgt mig, behøver jeg rende til fysioterapeut med jævne mellemrum? Også blev de glade når jeg sagde, "nej du bliver instrueret også skal du selv lave øvelserne derhjemme". Så der er klart en teoretisk fordel ved det, men jeg ved ikke om det er en praktisk fordel også, fordi du vil ikke sikre at der er compliance ved træningen.

RSH: Men du har faktisk oplevet at når du henviser til det her (QUADX-1), at de er glade for at de kan klare det selv der hjemme?

ORTHOPEDIC SURGEON 1: Ja. Men det er rent logistisk, fordi folk gider ikke rende frem og tilbage til en fysioterapeut for at lave en øvelse, de selv føler de vil kunne lave hjemme.

RSH: Nej nej, fremragende.

ORTHOPEDIC SURGEON 1: Men på samme måde har jeg oplevet, det er så ikke i dit projekt, men ved andet genoptræning hvor patienterne føler at de er blevet afsluttet for hurtigt, og blevet vist videre til selv-øvelser. Hvor det har haft den omvendte effekt. Igen jeg tror det er meget individuelt og hvordan folk er indstillet.

RSH: Ja, det, jeg tror at dem der er motiveret de synes, at de kommunale tilbud de generelt er korte, fordi de vil gerne blive ved i dem. Men faktisk, jeg har haft nævnt det her, men du hører selvfølgelig om mange projekter, men da jeg præsenterede det her første gang, vi har jo faktisk et mål for adherence til træningen eller tilslutningen, om de laver den. For vi har sat denne her sensor på elastikken. Så objektivt så finder vi ud af om de faktisk laver det. Så det bliver jo, vi kommer til faktisk til at få et billede af om de laver der her derhjemme ikke. Ja, det bliver mildest taly spændende.

JWK: Eller om det er konen der laver det, ja.

RSH: Ja det er så ulempen. Det kan jo være...

ORTHOPEDIC SURGEON 1: Da vores portører på afdelingen de fik sådan en skridttæller på et eller andet tidspunkt, for at finde ud af hvor mange skridt de går. Også inden for de første to dage så sidder de begge to sådan her på gangen (ORTHOPEDIC SURGEON 1 viser at portører overestimerer deres gang), for at vise at den var ?? (kan ikke forstå), så...

JWK: Hehe, ja.

RSH: Ja, jamen det er jo den klare ulempe. Altså, det kan også være børnebørnene der finder elastikken og sådan noget, men...

ORTHOPEDIC SURGEON 1: Men ja...

RSH: Der er nogle... Vi har faktisk prøvet at tage højde for det der, denne her gang, øhm. Det var umiddelbart nogle fordele ved det her med, at de for nogle redskaber. Kunne du se nogle ulemper ved det?

ORTHOPEDIC SURGEON 1: Det er igen compliance. Men det finder du så ud af. Altså, det er frihed under ansvar. Så hvis du er selv ansvarlig for din egen træning så er der heller ikke nogle der skubber til dig, så.

RSH: Nej. Hvis man kigger mere på det sådan, hvad mulighederne er for det her, med at de for nogle redskaber selv og der... Kan du se at det kunne have gang på jord ift. at hjælpe de her patienter og, at I (kirurger) måske ville se færre af dem, der ikke behøver komme ind til jer i virkeligheden?

ORTHOPEDIC SURGEON 1: Tænker du hvis du sammenligner selv-træning mod superviseret træning, eller?

RSH: Ja, bare generelt at patienterne de for nogle redskaber til selv og håndtere de her ting.

ORTHOPEDIC SURGEON 1: Igen, jeg tror det er meget individuelt. Jeg tror at nogle er rigtig gode til det, og nogle er bliver mere forvirrede af det. Så jeg tror at for mig er det ret svært at svare entydigt på det. For nogle, ja.

RSH: Ja. Tænker du noget der Jeanette?

JWK: Nej.

RSH: Så når vi ned mod noget af det sidste her. Og øh, det er det her, så er vi nået i virkeligheden herover på den side her (peger på modellen, efter opr).

ORTHOPEDIC SURGEON 1: Ja.

RSH: At de har potentiel bedre genoptræning. Så hvis en intervention har en effekt her (før opr), men de stadigvæk ønsker at få en operation, både fordi I føler at indikationen stadig er der og de stadig har ønsket. Teoretisk i hvert fald, så vil de jo få et løft her (før opr), som kan hjælpe dem til en lettere rehabilitering. Øh, så hvad, hvilke scenarier eller hvilke tanker har du omkring...

ORTHOPEDIC SURGEON 1: Ja, du tænker på på præ-hab i det her tilfælde har en effekt på outcome efter operation?

RSH: Ja, det er mere hvad du tænker om, det er fordi jeg har skrevet, to scenarier, men det ene scenarie det er jo at de vælger det fra, den kan vi lige tage bagefter, men den første her den var egentlig tænkt, at de har en lettere rehabilitering kva at de har fået noget træning. Hvad du...

ORTHOPEDIC SURGEON 1: Det ved du jo heller ikke. Altså, man kan argumentere for at, jeg tror der har været nogle studier der viser at præ-rehab virker, men du kan altid diskutere; er det det faktum at der er præhabilitering der har virker eller bare den samlede øgede mængde træning. Så hvis du sammenligner en patient der har fået tre måneders træning før operationen, og lad os sige tre måneders træning efter operationen. Også sammenligner du ham med en patient der ikke har trænet før operationen, men de er fuldstændigt identiske patienter, altså har trænet tre måneder efter operationen. Også kigger på hans outcome. Så ham der har fået præ-rehab vil klare sig bedre hypotetisk set, men så ved du ikke om det er fordi han er blevet opereret og genoptrænet før operationen at han er blevet bedre, eller fordi han samlet set har trænet i seks måneder ift. at den anden han trænede at tre måneder. Giver det mening? Så bør du i princippet sammenligne en patient der har trænet i seks måneder efter operationen med en der har trænet tre måneder før operation. Fordi så har du den samlede mængde genoptræning har været den samme. Og den måde er der ikke nogle der har stillet det op på, så...

RSH: Ej, det... Jaanejej...

ORTHOPEDIC SURGEON 1: Men selvfølgelig så kan du, så har du rykket genoptræningen til før operationen. Det er sikkert fint, igen jeg tror ikke at det skader, men jeg ved ikke om det hjælper.

RSH: Nej, det er netop det om, hvis... Lad os sige at det hjælper, hvad du har af tanker omkring det?

ORTHOPEDIC SURGEON 1: Jeg tvivler hvis jeg skal være helt ærlig, men...

RSH: At det (træningseffekt) kan bære igennem (til efter opr)?

ORTHOPEDIC SURGEON 1: Ja. Jeg tror at de studier man har vist, at den quadriceps-setback man for i forbindelse med operation, ikke på nogen måde kan blive genopvejet af den eventuelle gevinster ved operationen.

RSH: Nej, heller ikke hvis det er præ-operativt (træning)?

ORTHOPEDIC SURGEON 1: Ja, præcis, hvis du træner præ-operativt. Igen, jeg tror også fordi hvis, jeg tror ikke hvis træningen hjælper, så på tre måneder tror jeg at du kan opnå noget særlig stor effekt på, jeg ikke engang hvad det skulle være på, i forbindelse med resultatet efter operationen. Jeg har svært ved at se hvad det skulle have hjulpet. Mere muskelstyrke måske?

RSH: Ja, det er jo netop det at, øh, det der drastiske tab (i muskelstyrke) de har i quadriceps post-operativt, at hvis man kunne mindske det, så ville...

ORTHOPEDIC SURGEON 1: Det finder vi ud af. Jeg tror ikke på det, men jeg har ikke noget belæg for det.

RSH: Nej. Og det andet scenarie, det er jo selvfølgelig, øh hvad hedder det, nej det, det har vi jo været inde på.

ORTHOPEDIC SURGEON 1: Til gengæld skader det 100% ikke, så du ved, det er et gratis forsøg. Hvis det hjælper så er det jo super fint.

RSH: Ja. Jeg tror, jeg har egentlig ikke mere til denne her. Har du det Jeanette?

JWK: Nej, det har jeg faktisk heller ikke.

RSH: Også har haft jeg spurgt ind til hvad det er vi skal gøre for at du (ORTHOPEDIC SURGEON 1) kan huske at inkludere patienter. Hehe...

ORTHOPEDIC SURGEON 1: Det er fint nok, du har sat det der lable på, det skal jeg nok.

RSH: Er der ellers noget i forbindelse med det her, som set fra en kirugs synspunkt, som vi ikke lige har nævnt nu, som du...

ORTHOPEDIC SURGEON 1: Det ville være nemmere hvis vi kunne henvise alle patienterne, altså det der med kommunen (kun patienter fra København, Brøndby og Hvidovre) er bøvlet, men jeg forstår godt det er en logistisk udfordring. For os (kirurger) ville det være klart nemmere, hvis du kan henvise alle der skulle til, henvises til genoptræning, de skulle henvises til projektet, så at sige.

RSH: Nåå, så gruppen den var bredere?

ORTHOPEDIC SURGEON 1: Ja. Præcis, altså det ville gøre det nemmere for os. Nu har du gjort

det, så det er fint for os. Den der med 90, hvor der står 90 så skal der henvises. Så det andet er de andre der skal henvises ud i kommunen. Men hvis det ikke eksisterede så kunne man bare indføre en regel, og sige at alle patienter, som ikke har prøvet genoptræning og som er motiveret for det skal henvises til genoptræning. I stedet for at henvise til GLAD-træning, så henviser vi til træning i projektet.

RSH: Ja, okay.

JWK: Er der en barriere i det RSH?

RSH: Det ville blive en mere broget skare vi ville få ikke. Så vi ville have lide sværere ved at kontrollere hvem det er. Eller sagt på en anden måde, så skal vi have samarbejde med ALLE kommuner...

ORTHOPEDIC SURGEON 1: Ja ja, præcis, det er derfor jeg sagde, at jeg forstår godt det logistiske. Jeg synes heller ikke du skal løse det, fordi det skal være ligesom, det skal kunne betale sig. Jeg tror ikke det er indsatsen værd, men det er bare...

RSH: Fordi også i projektrammen så er det meget godt at vi ikke har mere end tre og holde styr på samarbejdsmæssigt ikke. Så det er jo en model vi prøver af, så hvis nu det her skulle rulles ud i større, så er det klart så ville det være nemmere at I (kirurger) bare kunne sige, uanset hvor patienten kommer fra.

ORTHOPEDIC SURGEON 1: Jamen jeg synes også i forvejen at det her er klart det nemmeste af de projekter vi har at inkludere i. Fordi, ikke at det ikke rigtigt er et projekt du inkludere i, i princippet vælger du som kirurg, hvilket du også har lov til, at vælge, hvilken genoptræning du henviser patienten til. Om du henviser til det her eller ude i kommunen er for mig fuldstændig hip som hap. At der virker at ringe til dig og sige at ham her, han skal træne.

RSH: Når du sidder dernede også du har, for nu har du jo lige pludselig en mulighed der hedder... Hvis nu du tegner det helt skarpt op, så var der før der var nogle, dem, de var ikke, de har ikke TKA, eller ikke slidgigt i knæet, så det er en hel kategori for sig. Så er der dem som har for lidt slidgigt til at du vil skære i dem, så dem kalder vi GLAD-kandidater. Også var der en kasse der hed, kandidater til operation. Hvor nu så står der en kasse i midten i virkeligheden...

ORTHOPEDIC SURGEON 1: Nej, det er der ikke. De patienter som ikke er kandidater til operation, de hører heller ikke til det her. Du kan godt henvise dem til GLAD, men selvom at... Før har du de samme tre kasser, dvs. patienter som slet ikke har slidgigt, som har et helt andet problem, som ikke har slidgigt, dem ser vi bort fra. Også er der dem med slidgigt som er kandidater til operation, og som ikke er kandidater til operation. Vi antager at ingen af dem har prøvet træning, så kan du godt henvise alle sammen til GLAD, også bagefter se om de skal opereres eller ej. Men hvis du før GLAD-træningen ikke var kandidat til operation, så bliver du næppe kandidat til operation efter GLAD-træningen.

RSH: Nej.

ORTHOPEDIC SURGEON 1: Så siger jeg at GLAD-træningen kan lige så godt blive erstattet af det her i mine øje. Det er jo fuldstændig hip som hap.

RSH: Ja, okay.

ORTHOPEDIC SURGEON 1: Men hvis du ikke er kandidat til operation, så ville jeg henvise til GLAD-træningen og afslutte patienten, men så kan han ikke indgå i det her, fordi han ikke er kandidat til operation. Giver det mening?

RSH: Ja, jamen det er det jeg mener med at øh (ORTHOPEDIC SURGEON 1 afbryder).

ORTHOPEDIC SURGEON 1: Det er stadigvæk tre kasser, men øh, så er der bare erstattet GLAD med det her (QUADX-1), hos de patienter der skal opereres. Eller måske skal opereres.

RSH: Ja okay, så før det her projekt så dem der, der var alligevel nogle du tænkte du ville operere, men du henviste til træning først?

ORTHOPEDIC SURGEON 1: Jaja, præcis det samme. Præcis det samme som det her.

RSH: Ja. Godt, jamen jeg har sådan set ikke mere, hvis I ikke har mere.

ORTHOPEDIC SURGEON 1: Alright. Adieux.

RSH: Adieux, tak for det xx. Jeg håber vi snakkes ved senere

ORTHOPEDIC SURGEON 2

RSH: Øh, ja så overskrifterne de lyder på den her koordinering af konservativ og kirurgisk behandling før operation, så det er omkring modellen. Så er der jeres rolle som ortopædkirurger i det her (modellen), hvilke barrierer der kan være for det. Øh, også er der en overskrift omkring det med at I herovre revurderer dem, på baggrund af at de har prøvet træning, hvad du tænker om det. Også er der lidt omkring at patienten i det her træningsforløb, i virkeligheden for nogle redskaber til selv at klare deres tilstand, potentielt, hvad du synes om det. Også er der lidt omkring at hvis de når hertil (efter opr), om det måske kan gøre deres rehabilitering bedre. Øh, men for at vende tilbage til starten, så er der det omkring koordinering af konservativ og kirurgisk behandling. Jeg vil gerne høre dine tanker er omkring, at kandidater til en total knæ-alloplastik tilbydes konservativ behandling i form af træning inden en eventuel operation. Hvilke fordele du evt. kan se i det?

ORTHOPEDIC SURGEON 2: Ja, altså der kan være en fordel i at de har bedre muskelstyrke, øh hvis de har været igennem det. Altså efter vores referenceprogrammer, der hedder det jo også at man skal have forsøgt konservativ behandling før man skriver dem op. Og reelt er der ikke så mange der har været til det (træning). Jeg henviser bestemt ikke konsekvent, men det eneste jeg egentlig er ret konsekvent med, det er at de skal have prøvet smertestillende behandling. Altså medicinsk smertestillende behandling.

RSH: Ja. Er der en særlig grund til at du ikke gør det særlig konsekvent?

ORTHOPEDIC SURGEON 2: Mmmjjjaaaaaa, altså, nej måske sådan lidt tvivl på hvor meget effekt der egentlig er i det. Altså, folk der tager smertestillende og har meget ondt og har tydelig artrose og den slags, jeg kan ikke, jeg tror ikke rigtig på at der er nogle af dem man slipper uden om (at operere).

RSH: Nej ikke allesammen, men nogle måske?

ORTHOPEDIC SURGEON 2: Måske.

RSH: Måske. Ja, så det er lidt, det er i virkeligheden fordi, at er det en gråzone effektmæssigt.

ORTHOPEDIC SURGEON 2: Ja, altså men det er sådan en blanding af vurdering af hvor mange gener de angiver at have, og os hvordan det ser ud på røntgenbilledet.

RSH: Så ift. dig selv som kirurg, hvad kunne fordelen være ved at for det her tilbud (træning før re-vurdering)?

ORTHOPEDIC SURGEON 2: Jamen, det er jo som jeg sagde før. Altså, måske kan det være en fordel ift. at de har bedre muskelstyrke, og det kan godt være at der er nogle der undgår operation. Man kan sige, det er jo ret omdiskuteret, og der kører også en hel del debat i øjeblikket (Roos vs. Krogsgaard). For at sige det pænt ikke. Om det overhovedet er, om det er rimeligt. Og jeg vil sige, hvis de (patienterne) har mange gener, de har smerter, de har artrosen, og man ved at det hjælper for mange med en knæ-alloplastik. Så tænker jeg også, det kan godt være at det (træning) hjælper, men derfra til at det holder lang tid, det har jeg sku meget stor tvivl på. Jeg har ikke noget at have det i

som sådan, men øh...

RSH: Nej, det man kan sige det er i hvert fald at, hvis, lad os sige at de (patienerne) har effekt af træning, så vil det jo være en livslang aktivitet de skulle fortsætte med. Øh, fordi 12 ugers træning, hvis du så ikke træner i 12 uger efter det, så er det (effekten) jo vasket ud. Så det jo i hvert fald være en intervention de skal fortsætte med. Det tror jeg måske er en af de ting der blive negliceret lidt. At fordi TKA'en er jo for evigt, eller, jeg ved ikke hvor længe de holder, 15 år eller et eller andet.

ORTHOPEDIC SURGEON 2: Ja.

RSH: Nå, så det var lidt omkring dig. Hvis du så skulle se det fra patientens synspunkt. Det her med at, øh, at de bliver tilbudt konservativ behandling før (operation). Hvad tror du de har af holdning til det?

ORTHOPEDIC SURGEON 2: Altså, der er nogle der er skeptiske ift. træningen, men der er også mange der er meget forbeholdne overfor operation. Og der vil jeg sige, at der har det jo en rigtig god (virkning?), så føler de at der sker noget og måske hjælper det også. Og jeg vil da godt sige, altså der hvor det her (QUADX-1) det har den helt store betydning for dem jeg ser. Det er dem, hvor der er noget slidgigt, de tager ikke specielt meget smertestillende, og hvor jeg har fornemmelse af at de har lidt urealistiske forventninger til forløbet i forbindelse med en operation. Altså, det nemmeste det er jo bare at skrive dem allesammen op, men så skal man kunne gå og se på dem hver tredje måned i meget lang tid bagefter. Fordi det ikke lever op til hvad de havde ventet. Og der vil jeg sige, der tror jeg at det her (QUADX-1), der kan det være en hjælp, dem der ikke har så meget artrose, men hvor man kan sige der er ikke andet og tilbyde.

RSH: Nej, når dem der har urealistiske forventninger, er det fordi, er din oplevelse at de kommer ind og tror at det er et "quick fix". Altså at operationen er klaret også nærmest dagen efter så er de fit for fight uden forløb efterfølgende.

ORTHOPEDIC SURGEON 2: Ja. Ja, eller siger at tage smertestillende det vil de ikke, øh og ja, hvor der ikke er de store forandringer, og hvor man tænker det her det, de er ikke klar over hvad det handler om. Så giver det (træning) et pusterum, og måske hjælper det også. Så det kan man jo håbe på.

RSH: Så det giver et pusterum til patienten, til at tænke en ekstra gang over hvad operation betyder, fordi I ligesom forfæller dem hvad konsekvensen vil være, øh, og også rent post-operativt forløb mæssigt. Øh, og men gør det, det så også nemmere for jer at tage snakken med dem anden gang, at de trods alt...

ORTHOPEDIC SURGEON 2: Det tror jeg egentlig nok. Altså, nu har jeg ikke haft nogle igennem fra det her (QUADX-1) endnu, der er kommet tilbage. Øh, men ellers generelt der har været nogle der har været igennem de der, nej fordi der er man sku tit afsluttet, har jeg ikke (spg til sig selv?).

RSH: Tænker du på GLAD?

ORTHOPEDIC SURGEON 2: Jo, det kan godt være, ja ja jeg tænker på GLAD, hvor mange jeg egentig ser der. Der ved jeg ikke om det er helt så konsekvent at de kommer igen. Eller om man

afslutter dem til det. Det er jeg lidt i tvivl om.

RSH: Ej, det er her hvor vi i hvert fald afviger fra GLAD, fordi jeg ved at der bliver henvist en del til GLAD herovre (peger på modellen) med dem der ikke er kandidater, men de for ikke nødvendigvis en opfølgning. Så de kommer lidt ud at svømme indtil de selv henvender sig igen.

ORTHOPEDIC SURGEON 2: Nej præcis. Men man kan så sige, at hvis de kommer igen og de stadig har ondt, så kan man sige så er det (træning) prøvet af også.

RSH: Ja. Øh, hvis man så tænker på det her sådan som, at det kører i øjeblikket med, at jeg sætter nogle mærker i din kalender, og du kan ringe til mig. Hvordan fungerer det her/passer ind i din daglige praksis ved at der er det her tilbud?

ORTHOPEDIC SURGEON 2: Ja, det fungerer rigtig godt. Det synes jeg.

RSH: Så hvis nu at det var rullet ud i større skala. At det faktisk var et koordineret tilbud med kommunen hvor det ikke var et projektfysioterapeut der stod for det, men faktisk at du kunne ordinere det her forløb. Ville det også kunne fungere?

ORTHOPEDIC SURGEON 2: Ja. Det ville gøre det nemmere, fordi at altså, det er jo lidt det problem vi har ift. GLAD-projektet. Der er jo i princippet er det samme ikke, men, eller næsten, men altså træning i hvert fald som du kan henvise til. Men det er en tand sværere, og de skal selv finde det og det er ikke alle steder at det er der, og det er forskelligt hvor meget de skal betale osv.

RSH: Så det ville være nemmere og en fordel, hvis det var rullet mere sammen al koordination osv.

ORTHOPEDIC SURGEON 2: Ja. Det synes jeg.

RSH: Hvis nu, sådan rent organisatorisk set, hvem, hvis ansvar skulle det så være at det her fungerede?

ORTHOPEDIC SURGEON 2: Hvad tænker du? Om de bliver henvist eller...

RSH: Ja, I sørger selvfølgelig for at diktere også henvise, men hvor skulle ansvaret ligge? Er det, ville det være sådan en lægefaglig opgave eller en fysioterapeutisk opgave, eller er det en administrativ ting?

ORTHOPEDIC SURGEON 2: At de bliver henvist eller at de for træning...?

RSH: Ja, at de kører, at kommunikationen over sektorgrænser fungerer.

ORTHOPEDIC SURGEON 2: Det er vel næsten på fysioterapeutsiden, altså. Det ved jeg sku ikke rigtigt. Altså, hvem har ansvaret for at de kommer til træning, at de kommer tilbage, eller...?

RSH: Ja, om de kommer til træning, det er jo i virkeligheden deres eget ansvar. Men det er mere om, at tiderne bliver booket på begge sider af sektorgrænserne osv. Men det skal der i

virkeligheden nok sidde en eller anden koordinator og gøre jo. Dårligt spørgsmål, he.

ORTHOPEDIC SURGEON 2: Det er lidt svært. Altså, hvis du tænker sektorgrænser, tænker du det, altså...

RSH: Primær og sekundær. Kommune og hospital.

ORTHOPEDIC SURGEON 2: Altså, hvad er det vi gør nu? Der sender vi en genoptræningsplan... Når det er GLAD. Altså, det kan vi jo godt. Det er jo sådan set ikke...

RSH: Så det ville i virkeligheden være samme model, som I bruger nu ikke?

ORTHOPEDIC SURGEON 2: Jo, det synes jeg. Og den opfølgende tid, den kan vi jo også booke med det samme hos os selv.

RSH: Ja, så i virkeligheden samme organisation som nu?

ORTHOPEDIC SURGEON 2: Ja.

RSH: Så er der lige sådan. Er der noget du tænker omkring det her (QUADX-1), nu har det kørt noget tid? Jeg ved godt du ikke har oplevet nogle opfølgninger, men da jeg præsenterede det for dig, og nu har du oplevet det en lille smule. Hvilke faktorer, har du tænkt over noget du synes jeg skal være opmærksom på?

ORTHOPEDIC SURGEON 2: Nææ, det synes jeg egentlig ikke. Jeg synes det, nej der det, og det gør det rigtig nemt når du ovenikøbet har kigget listerne igennem og set hvem der er potentielle kandidater. Så nej det synes jeg egentlig ikke.

RSH: Nej, ok. Så går vi videre til et nyt emne. Og det handler mere om din rolle som ortopædkirurg i denne her koordination af præ-operativ træning, altså konservativ og kirurgisk behandling. Og hvilke barrierer der kan ligge for konservativ behandling hos en kirurg, som jo er uddannet til det operative. Øh, så hvilken rolle føler du, at du har i det her med at koordinere, eller veje de to tilbud overfor hinanden?

ORTHOPEDIC SURGEON 2: Jamen det ved jeg sku ikke. Altså, det afgørende det vel egentlig og få, som vi var inde på tidligere og få forventningsafstemt. Gøre klart hvad det er det handler om med operation. Øhm, også jo også noget med man... Altså, jeg skal selv have en tro på, at det er det rigtige med træning frem for operation. Fordi hvis det er et eller andet med, jeg tænker, at jeg kan godt sende til træning, men der kommer præcis samme resultat tilbage, fordi knæet er slidt ned. Selvom de ikke har prøvet at træne, altså. Øh, også tænker jeg lidt, så er det egentlig bare og udskyde en, den behandling de bør have i tre måneder.

RSH: Ja. Okay, hvad så med, hvis de (patienterne) kommer ind, og de er meget uvidende omkring deres, rækken af tilbud der faktisk er, ja, eller forskellige behandlingsmuligheder. Ser du det som din rolle, også og nævne for dem, at der findes træning?

ORTHOPEDIC SURGEON 2: Ja, klart, klart. Jaja, ja, ja. Altså, når de kommer, så må man se hvad muligheder der er.

RSH: Ja. Så det er, på trods af at du i virkeligheden er uddannet til den ultimative behandling til sidst, så de andre ting bør også nævnes fra din side?

ORTHOPEDIC SURGEON 2: Ja, det gør det. Også tage op også hvad der er afprøvet ikke, altså. Og der er jo mange der rent faktisk har taget, ikke så mange der har trænet specifikt, men rigtig mange der har taget smertestillende.

RSH: Ja. Altså, ja. Dem der kommer ind ikke og har trænet (ikke prøvet træning endnu), er det i højere grad dem der, det bliver nævnt overfor at det er en mulighed? End dem der har trænet en hel masse.

ORTHOPEDIC SURGEON 2: Jeg vil sige, at hvis det er en der har svære forandringer, og tager fast smertestillende, og har mange gener. Der vil jeg sige, der har jeg selv meget meget lidt tro på, at man kan træne sig ud af det.

RSH: Okay. I de tilfælde der nævner du det (træning) måske ikke?

ORTHOPEDIC SURGEON 2: Nej.

RSH: Nej, øh det leder lidt ind på det her. Så den patient, som du vil se som en god kandidat til at gennemgå det her (træning). Det er i virkeligheden en hvor du har lidt mere tro på det? Både ud fra hvad de fortæller og har prøvet, og deres radiografiske (forandringer)...

ORTHOPEDIC SURGEON 2: Ja.

RSH: Øh, ja vi har også snakket lidt om hvem du. Og dem der ikke vil have gavn af det, det er dem, hvor forandringerne er for store til, at du har tro på det også (at træning vil have effekt)?

ORTHOPEDIC SURGEON 2: Ja.

RSH: Så er der sådan en lidt varm kartoffel. Hvilke hindringer ser du som, for jer som kirurger, i og bruge træning som en fast behandling til de her patienter? Der tænker jeg på hele din faggruppe. Ikke bare dig personligt.

ORTHOPEDIC SURGEON 2: Hvad sagde du, en gang til. Hvilke hindringer?

RSH: Hvilke hindringer der kunne være for, at det her (træning) bliver systematisk tilbudt til patienten?

ORTHOPEDIC SURGEON 2: Altså, for mig som faggruppe, jamen så er det vel egentlig lidt at øh, at vi selv skal have lidt tro på det, før vi begynder og gøre det, når vi har det andet alternativ.

RSH: Mmmhhhm.

ORTHOPEDIC SURGEON 2: Og, ja altså det er lidt det samme som jeg sagde før synes jeg, ikke. Med at hvis man, hvis man ikke, hvis vi ikke har troen på det, og vi ved at der findes en behandling.

RSH: Ja...

ORTHOPEDIC SURGEON 2: Så er det jo en barriere.

RSH: Det er det absolut. Jeg tænkte lidt på, at hvis nu at øhm. Lad os sige at det faktisk er ret effektivt, og der er mange patienter der er glade for det. Hvis det så betød at der var mindre arbejde til jer, ville det? Jeg ved vi er ude i ekstremen.

ORTHOPEDIC SURGEON 2: Åh ja eehhh, øhm, det tænker jeg sku ikke rigtigt på. Altså, det er ikke de overvejelser der ligger der. Det er det ikke.

RSH: Nej, okay. Så er der lidt, hvis vi går her hen i, på tidslinjen, når du ser dem anden gang. Øh, så formålet med projektet det er jo som sagt, at vi ligesom giver, at patienten er vurderet på det bedste grundlag, hvor konservativ behandling er prøvet af inden de eventuelt bliver tilbudt operation. Føler du også at det vil give dig et bedre grundlag, når de kommer ind? Du ved hvad det er de har, de har gennemgået den her træning. De skal re-vurderes. Gør det det også nemmere for dig at tage snakken (om operation) med dem så? Qua deres...

ORTHOPEDIC SURGEON 2: Ja, det gør det jo nok. Ja, altså, på samme måde som når de har prøvet fast smertestillende af i en periode. Øh, også kan man sige, at hvis de har prøvet det her af også (træning), og de stadigvæk har gener, så kan man sige, så gør det det jo så lidt nemmere og skride til operation.

RSH: Ja, så det med...

ORTHOPEDIC SURGEON 2: Selvom man kan sige. Altså, hvor man stadigvæk kan have sine tvivl ikke. Altså, der er mange hvor man stadig har fornemmelsen af at, det kan godt være de har trænet, og de synes de har utåleligt ondt, øh, men de kliniske fund og de radiologiske fund er beskedne. Og der kan man da godt tænke, "hva, hvordan skal det ende det her?", ikke. Men man kan sige, der, det er prøvet af. Og hvis de så siger, "jamen noget må der gøres", så er det det (operation). Så på den led kan det, da godt gøre det nemmere.

RSH: Ja, så i virkeligheden at der er nogle check-bokse der er klikket af?

ORTHOPEDIC SURGEON 2: Ja.

RSH: Er der noget rart i det, når man sidder overfor patienten? At man kan, nu nævnte du selv de nationale retningslinjer, jeg ved ikke hvor meget de ligger i baghovedet og kører men, man har listen over at de skal prøve smertestillende, de skal prøve konservativ også til sidst (operation). Er det, er det noget du tænker over, eller er det sådan...?

ORTHOPEDIC SURGEON 2: Mmmjjaannnaaa, altså jeg tænker ikke så meget specielt, at nu lever jeg op til retningslinjerne. Det er ikke så meget det. Det er mere det der med om man tror det er det rigitge at gøre.

RSH: Ja, for patienten?

ORTHOPEDIC SURGEON 2: Ja.

RSH: Vi har været en lille smule inde på det, men bare for at være på den sikre side. Hvad tror du patienterne vil synes om det her (modellen)?

ORTHOPEDIC SURGEON 2: Jamen, der er mange der er glade for det, fordi de netop selv er lidt forbeholdne overfor operation, også føler de at der sker et tiltag.

RSH: Ja. Dem der er, når du sidder nede i ambulatoriet og du har det her tilbud til dem, dem der er mest for det, er det dem, er der er tendens til at det er dem der er bange for operationen?

ORTHOPEDIC SURGEON 2: Ja.

RSH: Det er mest dem der er for det. Dem der kommer ind og er indstillede på operation, er de sværere og overtale?

ORTHOPEDIC SURGEON 2: Jeg kan sku, jeg kan ikke huske hvor mange jeg har haft af dem. Altså, det kan jeg ikke svare på. Jeg kan ikke huske det specielt.

RSH: Men der er en tendens til, at det er dem der er bange...?

ORTHOPEDIC SURGEON 2: Ja, det er mere dem der kommer, og som ikke rigtigt ved hvad det egentlig er med en knæ-alloplastik. Og, altså, som er rimeligt uforberedte når de kommer. Der er det godt, ja.

RSH: Ja. Øh, så er der jo det her. Sådan som træningen er sat op her, så er det jo én øvelse, som de kan lave derhjemme selv. Så de er ret, øhm, der bliver lagt en del ansvar over på dem selv for selv at få gjort noget ved det her. Så de for i virkeligheden, hvis de kan håndtere det, så for de sådan set et redskab til selv og kunne styre deres smerter. Fordi som sagt så ville de, hvis de træner i 12 uger, og oplever det faktisk gør deres knæ-symptomer bedre, øh også se kommer ind her (peger på model ved t1) og snakker med dig, og I siger nej tak til operation i fælleskab. Øhm også de går videre også og glemmer lidt og træne, i og med at de ikke er med i projektet mere, så er det faktisk noget de kan hive frem igen og kan opleve at de kan regulere det her. Er det en god ting synes du, at man kan give dem et redskab.

ORTHOPEDIC SURGEON 2: Ja, selvfølgelig. Ja, vist er det det. Ja, og det vil jeg da også sige til dem ikke, at "husk og bliv ved", og ?? og så videre. Jeg forestiller mig, som du selv er lidt inde på, at der er rigtig mange der, når de har overstået de 12 uger ligesom tænker "det var det". Altså, jeg ser det ligesom slankekurer, ikke. Som folk de kæmpe sig igennem også går der et halvt år, også er de oppe igen.

RSH: Ja, så det er virkeligheden en potentiel ulempe ved det, at det trods alt er noget de skal, at det er livslangt for dem det her. Der er ikke nogen kort vej.

ORTHOPEDIC SURGEON 2: Ja.

RSH: Og det, ser du det som en barriere for at de, altså lad os sige at de gennemfører

projektet og er en af dem der hopper fra operation. Også konkluderer, vi at det er rigtig godt, men så ser du dem et halv år senere alligevel, fordi de er stoppet med at træne. Det er en faldgruppe?

ORTHOPEDIC SURGEON 2: Ja, det kan man sagtens forestille sig. Og der er jo så stor forskel på patienterne. Der er jo rigtig mange der meget har den holdning, at når de kommer her så bliver tingene gjort for dem.

RSH: Ja.

ORTHOPEDIC SURGEON 2: Og de selv er lidt overraskede over hvor meget de selv skal træne, også efter operationer. Øh, også er der andre der, som udgangspunkt er meget opmærksomme på deres krop og deres træning osv ikke. Der er jo nogle, der også når de kommer nu, går i fitness, øh fast eller et eller andet ikke.

RSH: Jo jo. Oplever du at der er nogle der ligefrem, hvis altså du siger "der er svær slidgigt og en operation det vil være det rigtige for dig", og du så fortæller dem om den træning de så skal ligge efterfølgende, at de så siger nej tak pga. det?

ORTHOPEDIC SURGEON 2: Nej.

RSH: Trods alt ikke?

ORTHOPEDIC SURGEON 2: Nej nej.

RSH: Så den er de villige til at bide i sig?

ORTHOPEDIC SURGEON 2: Ja. Nej, det har jeg ikke været ude for.

RSH: Nej, det ville også være, det ville overraske mig en lille smule. Ja, så noget af det afsluttende her, det er omkring, vi har været lidt inde på det. Men om du, hvis træningen har effekt her (før opr), men de trods alt stadigvæk, den har ikke haft stor nok effekt, så de kommer stadigvæk ind til dig og siger "det er ikke blevet markant bedre". Og I bliver enige om at operationen, det er nok den rigtige vej at gå. Har du så tro på at den intervention der ligger her (pre-op), konservativt med træning, kan gøre deres rehabilitering bedre? Eller hurtigere, nemmere.

ORTHOPEDIC SURGEON 2: Måske lidt. Jeg tror vi er ude i de små størrelser. Altså, ift. hvor meget nemmere det skulle være. Og i virkeligheden også hvad man måler på. Om det er bevægelighed eller, knæ-score, smerte-score. Altså, hvor hurtigt det/de kommer ikke. Deeeeet...

RSH: Nej, det er jo også et ubelyst område.

ORTHOPEDIC SURGEON 2: Jeg vil sige. Ja, og man kan sige. Et eller andet sted så tænker jeg, det er sku heller ikke helt så vigtigt altså. Øh, hvis der var en markant forskel ikke, men et eller andet sted så ser jeg, så er træningen og den træning de for, det er jo mest et spørgsmål om at få dem i gang, og få dem hurtigt i gang. Specielt det sidste ikke, fordi hvis du måler på det, mener jeg så stadigvæk det gælder ikke, efter et år der kan du ikke måle hvem der har fået træning, og hvem

der ikke har fået det.

RSH: Øhhh. Nå, nej, post-operativt?

ORTHOPEDIC SURGEON 2: Post-operativt træning.

RSH: Nej, det var det xx bl.a. var med til at vise.

ORTHOPEDIC SURGEON 2: Men igang skal de jo. Ja.

RSH: Ja. Så du tvivler i virkeligheden lidt på om det her kan bære igennem og gøre en forskel?

ORTHOPEDIC SURGEON 2: Ja, jeg tvivler lidt på om det hjælper bagefter. Men det er ikke det samme, som at jeg siger. Jeg vil da sikkert sige til dem, at det sikkert også kan hjælpe bagefter. For hvis de selv har en positiv tilgang, altså en tro på at det hjælper uanset hvad, og de selv tænker "jeg er i bedre form til det". Altså, hvis de selv har troen på, at de kan noget og gør noget. Så tror jeg også på, om det så er placebo eller hvad, men at det hjælper dem.

RSH: Ja. Jeg har jo også interviewet de fysioterapeuter der hjælper ude i kommunerne med at instruere. Og de var blandt andet inde på, jeg tror alle tvivler lidt på om det kan bære igennem, og ligefrem at de har et højere udgangspunkt postoperativt. Men om ikke andet så har de en eller anden form for en forståelse, og måske en, startet en træningskultur op, så de ved hvad det er og kender øvelserne, og sådan noget ting. Så er vi selvfølgelig ude over det fysiologiske, det er mere en kultur-indstillingsmæssig ting.

ORTHOPEDIC SURGEON 2: Ja.

RSH: Øh, så afslutningsvis her. Har du, øhm, kan jeg gøre mere for, at det er nemmere for dig at huske patienterne?

ORTHOPEDIC SURGEON 2: Nej. Nej, det synes jeg ikke. Det er helt som det skal være, ja.

RSH: Godt. Så, min fornemmelse er at, da jeg startede projektet op, så når man kommer med noget nyt, så er det jo altid at man ligger noget nyt på jeres bord, og noget nyt i skal forholde jer til. Jeg fornemmer lidt der har været sådan en ændring i holdning, også lidt fra din side, at du er blevet lidt mere positivt stemt eller åben overfor det. Er det forkert tolket?

ORTHOPEDIC SURGEON 2: Tjooo, ja lidt. Altså, men det er da rigtig nok, altså i starten når der kommer de der nye tiltag, så er det da, uanset om det er det her et projekt, eller det er et af de andre knæ-projekter, så er det da sådan i hups hvad nu, og hvad skal jeg huske og hvad skal jeg gøre. Altså, indtil man har prøvet det nogle gange, og finder ud af at hvad forretningsgangen er, og hvor

man skal ringe og hvad der skal inkluderes. Altså, indtil man lige har prøvet det af. Og det er da rigtig nok. Når det er på plads, så er det nemmere. Men lige i starten er det da "nå ok, nu skal vi også...".

RSH: Ja ja.

ORTHOPEDIC SURGEON 2: Så, men mere ligger der ikke i det.

RSH: Så det er i virkeligheden mere fordi, du har fået en ide om hvilke størrelse er det jeg har med at gøre her?

ORTHOPEDIC SURGEON 2: Ja ja. Og finde ud af rutinen når det er der.

RSH: Ja ja, nå det er godt. Øh, har du noget. En eller anden kommentar, noget vi ikke har været inde på....?

ORTHOPEDIC SURGEON 2: Nej, det synes jeg ikke rigtig.

RSH: Nej, okay.

ORTHOPEDIC SURGEON 2: Nææ, det synes jeg ikke jeg kan komme på, nej.

RSH: Alright, jamen det var det xx.

ORTHOPEDIC SURGEON 2: Godt.

ORTHOPEDIC SURGEON 3

RSH: Godt så er vi i gang. Så det første det drejer sig omkring det her med koordinering af konservativ og kirurgisk behandling før en evt. operation. Så hvad er dine umiddelbare tanker omkring, at kandidater til en total knæ-alloplastik tilbydes konservativ behandling i form af træning inden en eventuel operation?

ORTHOPEDIC SURGEON 3: Det er jo fint nok. Hmm, jeg tror ikke rigtig på at det hjælper, men det er så en anden sag.

RSH: Nej, kan du uddybe det?

ORTHOPEDIC SURGEON 3: Jamen det er ud fra de ting der ligger, de videnskabelige undersøgelser der ligger. Uden at jeg har nærlæst dem. Der kører jo en hvis debat, bl.a. i Ugeskriftet, f.eks. med Michael Krogsgaard. Ej, jeg tvivler på at det hjælper, men det kan jo aldrig skade.

RSH: Nej. Hvis øh, hvis nu der skulle, er der, kan du se nogle fordele ved at de for tilbudt det her træning?

ORTHOPEDIC SURGEON 3: Man kan sige, træning er jo altid godt. Har de sukkersyge, er de fede, hvad ved jeg, så er træning altid godt. Men det kan også være at det bringer dem i bedre stand hvad angår knæet inden de bliver opereret, men det har jeg ikke nogen viden om. Det er jo rent gætteri, det er der jo ingen undersøgelser om.

RSH: Nej. Det er mest rent ift. kirurgi, jeg tænker på det her om det er interessant.

ORTHOPEDIC SURGEON 3: Det ved jeg ikke.

RSH: Så du ved ikke om der kunne være en fordel ved at de trænede, eller har en holdning til det?

ORTHOPEDIC SURGEON 3: Teoretisk set ja, men jeg ved det ikke.

RSH: Nej, okay. Potentielle ulemper?

ORTHOPEDIC SURGEON 3: Ingen, ingen.

RSH: Nej. Øh, og det er jo så... Hvis nu, det er jo sådan umiddelbart, så snakke vi her om fordele for patienterne. Det er at de kan blive i bedre form. Jeg går ud fra at det også er det du tænker.

ORTHOPEDIC SURGEON 3: Mmmmm.

RSH: Hvad med dig som kirurg? Er der nogle fordele ved, at du kan tilbyde dem træningen inden de evt. bliver opereret?

ORTHOPEDIC SURGEON 3: En fordel for mig? Det forstår jeg ikke.

RSH: For dig som kirurg. Er det godt at du har et ekstra behandlingstilbud?

ORTHOPEDIC SURGEON 3: Æhhh, næ, jeg forstår det egentlig ikke, altså spørgsmålet. "Godt jeg har et ekstra behandlingstilbud"?

RSH: Ja. Ser du det som en fordel, at du også kan, udover at du kan tilbyde... (kan ikke høre)

ORTHOPEDIC SURGEON 3: Lad mig sige det sådan her. De patienter som kommer og har så lidt forandringer på røntgenbilledet, f.eks. fra artroskopørerne, der siger "nu kan jeg ikke komme videre, du må over og snakke med en af alloplastik lægerne", så kan jeg parkere dem på træningen. Fordi jeg ikke vil operere dem.

RSH: Ja.

ORTHOPEDIC SURGEON 3: Det kan være en fordel. Så må de ud og træne, også parkerer jeg dem derude. Fordi jeg ikke mener de skal opereres med en knæ-alloplastik. Men de kan komme fra artroskopilægerne, der siger til dem "jeg kan ikke gøre mere for dig, nu må du over til alloplastik-lægerne". Og øh, så kommer de over til mig, og de har ikke artrose. Også vil jeg jo ikke operere dem. Men de har nærmest fået at vide derovre, at de skal opereres. Så kan jeg parkere dem på træningen.

RSH: Og "parkere dem på træning", er det så en midlertidig løsning, eller hvordan?

ORTHOPEDIC SURGEON 3: Det ved jeg ikke, for jeg afslutter dem.

RSH: Du afslutter dem?

ORTHOPEDIC SURGEON 3: Ja, så jeg skal ikke operere dem, så skal de jo ikke komme hos mig mere.

RSH: Nej. Jeg tænker på dem som du faktisk ser som en mulig kandidat, altså de kommer ikke fra, nødvendigvis fra artroskopierne, men fra egen læge og har faktisk også radiologisk forandring osv. Er det en fordel at du kan tilbyde dem træning?

ORTHOPEDIC SURGEON 3: Mmmmnnnnnjjjjaaaa, altså som jeg siger til dig. I bund og grund så tror jeg ikke rigtig på at det hjælper.

RSH: Nej.

ORTHOPEDIC SURGEON 3: Så kan det være svært at se fordelen.

RSH: Og er det baseret på dine erfaringer med dem der f.eks. har været til GLAD og kommer ind til dig, eller er det baseret på videnskab?

ORTHOPEDIC SURGEON 3: Dem er der så trods alt ikke så mange af. Men jeg vil sige, at med den, dem jeg har set, så kommer de fleste og vil opereres eller også ringer de ind og vil ikke opereres, efter at de har været til GLAD-træning. Det gør de, det synes jeg.

RSH: Ja, okay. Så der er, også baseret lidt på, at du har oplevet patienter der har været ude og prøve, også har det ikke haft tilstrækkelig effekt.

ORTHOPEDIC SURGEON 3: Ja.

RSH: Øh, okay. Hvis vi snakker om det her som model. Hvordan passer det ind i din daglige praksis, med at du kan henvise til noget træning også kommer de ind til en revurdering. Jeg tænker sådan på dit daglige virke nede i ambulatoriet?

ORTHOPEDIC SURGEON 3: Jamen det passer... Jeg forstår ikke rigtigt spørgsmålet.

RSH: Om det er nemt og tilgængeligt for dig, eller om det er en ulempe eller sådan en større arbejdsbyrde?

ORTHOPEDIC SURGEON 3: Det er en ulempe i den forstand, at hvis der kommer flere patienter i ambulatoriet. Vi har forrygende tralvt i forvejen og ikke plads til patienterne. Så det at en patient skal komme til flere besøg er en ulempe kan man sige. Men det er jo rent, det er fordi der simpelthen er så travlt. Ja, patienterne kommer til at vente længe.

RSH: Ja, okay. Så det med at du ser dem en gang, og booker dem en gang til....

ORTHOPEDIC SURGEON 3: Ja. Hvis nu jeg bare satte dem op til operation, så havde jeg sparet et besøg, kan man sige, ikke. Men det er jo, du forstår hvad jeg mener ikke? Det er fordi jeg har et meget tralvt ambulatorium, hvor jeg faktisk ikke har plads til alle de patienter jeg skal se. Og mange af dem må vente for længe.

RSH: Ja. Hvad hvis det ikke var en tidsmæssig ting, men i den perfekte verden, hvor der faktisk var tid til at du lave femten ugers opfølgninger til dem der (kan ikke høre)?

ORTHOPEDIC SURGEON 3: Jamen, så var det jo fint nok.

RSH: Ja, så rent lavpraktisk så er det en tidsmæssig ting, der gør at det er besværligt.

ORTHOPEDIC SURGEON 3: Ja, det må man sige.

RSH: Og hvis man skal tænke på det sådan lidt mere organisatoriske, hvem har så ansvaret for at det her det fungerer? Er det, altå en koordination mellem kommune og hospital, vil det være jer som kirurger, eller er det den kommunale genoptræning der har ansvaret for at det ville fungerer?

ORTHOPEDIC SURGEON 3: Ah, hvad?

RSH: Af koordinationen mellem at de bliver henvist til noget genoptræning, også skal genbookes herinde 15 uger senere. Så det der med at de skifter sektorgrænser.

ORTHOPEDIC SURGEON 3: Øh, det forstår jeg heller ikke, hehe.

RSH: Hehe. Jeg prøver lige at omformulere det. Hvis, det her med at de er herovre (peger på modellen)... (xx afbryder).

ORTHOPEDIC SURGEON 3: Altså jeg ser jo bare patienten, også sender jeg dem til træning, også for de en tid efter 15 uger. Så jeg gør jo ikke mere.

RSH: Ja, så hvem har ansvaret for at det fungerer?

ORTHOPEDIC SURGEON 3: Jamen, er det ikke dig der booker tiden efter 15 uger, eller er din min sekretær. Det ved jeg ikke engang.

RSH: Nej, altså hvis det ikke var et projektsammenhæng, og det faktisk var noget som var implmenteret, så ville du mene det var sekretærens job.

ORTHOPEDIC SURGEON 3: Jaja, så er det sekretæren der skal holde styr på det. Jeg skal lave mindst muligt i den sammenæng.

RSH: Ja. Og det med at der kommer besked til kommunen, det er også sekretærens ansvar?

ORTHOPEDIC SURGEON 3: Ja, det skal det være. Det skal være ude af mit. Jeg skal bare ordinere det, også er der nogle der skal tage sig af det, og sætte det i værk.

RSH: Mmm. Er din fornemmelse at det her, det ville kunne være noget man faktisk implementerede? Hvis nu vi finder ud af at denne her trial den har et positivt outcome. At vi faktisk oplever nogle patienter, at de enten vælger fra eller har en bedre rehabilitering.

ORTHOPEDIC SURGEON 3: Hvis det viser sig, at der er patienter som for det så meget bedre, så de ikke skal opereres, altså så man kan dokumentere at de har en effekt af det her. En blivende effekt, det skal jo ikke vare tre måneder, eller seks måneder. Det skal være en langsigtet effekt. Så ville det være rigtig fint, absolut. Men det skal være en langsigtet effekt. Det er jo det der kan, problemet med træning, hvor lang tid holder det egentlig. Så ville det være fint. Og hvis man også har det sådan, at hvis det også viser sig at det har en effekt på dem der opereres, så de post-operativt har en bedre rehabilitering. Så ville det også klart være noget man skulle bruge.

RSH: Så ville du også være positivt stemt overfor det?

ORTHOPEDIC SURGEON 3: Jaja, selvfølgelig. Men jeg skal vide det.

RSH: Ja, det er klart. Hvis vi så, nu snakker vi lidt projekt generelt, hvis der er, hvilke faktorer vil du sige at jeg skal være opmærksom på i det her projekt? Nu når du har snuset lidt til det. Er der noget du har, du tænker, det skal vi lige være obs på?

ORTHOPEDIC SURGEON 3: Nææ, altså. Nej, jeg har ikke lagt mærke til noget, nej.

RSH: Okay. Så går vi videre til en anden overskrift, og det handler mere om din rolle som kirurg i denne her koordination af de to forskellige tilbud. Øh, og det er lidt omkring, om der ligger nogle barrierer i at du er kirurg? Om du har nogle barrierer overfor det at tilbyde træning lige så nært som en operation?

ORTHOPEDIC SURGEON 3: Det har jeg ikke. Altså, vi kører et projekt, og hvis jeg så nævner det for patienten, det har jeg ikke. Det er okay.

RSH: Så du tror ikke at der er, at du er lidt mere pro en operativ kirurgisk behandling end en konservativ behandling, hvis nu du skulle vælge mellem de to?

ORTHOPEDIC SURGEON 3: Det øhm, det er så individuelt. Det kommer også an på patienterne, hvad har de været igennem før, hvor lang tid har de haft ondt og så videre. Men som du selvfølgelig kan forstå ud af det første jeg har sagt, så tvivler jeg på at det hjælper. Men dermed skal det jo ikke være uforsøgt.

RSH: Nej. Øhm, så jeres rolle, det er det jeg fisker lidt efter, hvordan jeres rolle er i at koordinere mellem de her to tilbud. Så når patienten kommer ind til dig. Ser du så altid, inden du har snakket med dem, der er to muligheder, der er noget konservativt og der er noget kirurgisk behandling at tilbyde. Eller er du typisk foruindtaget?

ORTHOPEDIC SURGEON 3: Nej, jeg er fuldstændig fordomsfri.

RSH: Okay. Så hvis vi ser, at der så er en patient, som faktisk er en god kandidat til at deltage i det her projekt. Hvordan vil du beskrive vedkommende? Altså en der ville have godt af et træningsforløb, inden en evt. operation.

ORTHOPEDIC SURGEON 3: Patient med knæsmerter, let til moderat artrose, der ikke har forsøgt konservativ behandling før.

RSH: Ja. Og hvis de så har prøvet konservativ behandling uden effekt?

ORTHOPEDIC SURGEON 3: Tja, altså, de kan godt prøve det igen, men sandsynligheden, i mine øjne, for at det hjælper er selvfølgelig mindre, end hvis de ikke har prøvet det før.

RSH: Ja, okay. Så er der lidt den modsatte. Om der er nogle patienter du ser dernede, hvor du tænker, de her de vil ikke have gavn af at prøve træning.

ORTHOPEDIC SURGEON 3: Ja, det kan der godt være. Det er patienter med svær artrose, som har møj-ondt, ikke kan sove om natten og stort set ikke kan noget. De skal ikke træne.

RSH: Så dem ville du i virkeligheden...

ORTHOPEDIC SURGEON 3: ...og som har haft ondt i lang tid ikke. Og som har et knæ der er brændt sammen rent ud sagt.

RSH: Ja. Så der vil du ikke, det ville du måske være tilbøjelig til at sige "du behøver ikke prøve træning af først, lad os lave en operation med det samme"?

ORTHOPEDIC SURGEON 3: Ja. Det er jo typisk patienter som har for meget smertestillende ikke. De kan ikke komme op og ned af trapperne dårlig nok, altså. Jeg vil sige, men det er jo, jeg vil sige at de undersøgelser der ligger på, at der måske er en effekt (af træning), det som jeg stadig mener er et sjus om, det er let til moderat artrose. Og ved svær artrose der tror jeg ikke det har nogen gang på jord.

RSH: Ok, nej. Øh, så den næste den drager/driver lidt videre i samme. Så det er ift. hvilke hindringer du ser ift. systematisk og bruge træning som behandling til knæ-artrose patienter.

ORTHOPEDIC SURGEON 3: Det er selvfølgelig et spørgsmål om patienterne de nu også gør det. Om der er, hvordan compliance er ikke. Fordi man kan jo godt sende patienterne hjem og sige de skal træne. Spørgsmålet er så, for de virkelig gjort det eller ej, ikke? Det er jo problemet i det. Og det er også problemet når de selv-træner derhjemme. Du har ikke nogen kontrol på om de virkelig gør det eller ej. Det er jo det, der kan være problemet med det ikke.

RSH: Jo, absolut. Er der nogle hindringer set fra patienternes synspunkt? Altså, hvis du tilbyder dem træning, møder du så modstand nogle gange?

ORTHOPEDIC SURGEON 3: Altså, der er jo patienter, der har så ondt så de, som er nået dertil mentalt, også via det de har talt med deres egen læge, at de er nået dertil at operation ligesom er deres eneste mulighed.

RSH: Mmmm, okay.

ORTHOPEDIC SURGEON 3: Øhm, også er det så spørgsmålet om de forstår hvordan de skal træne. Det er jo ikke alle der nødvendigvis forstår det, selvom de måske siger ja og har fået instruktionen.

RSH: Ja. Hvad med, er der nogle der kommer ind og sådan nærmest har en bestillingsseddel på en knæalloplastik? Oplever du det?

ORTHOPEDIC SURGEON 3: Arrrrgghh, det er ikke så mange. Men der er nogle stykker ja. Og det er jo de sværeste at tale fra at blive opereret, hvis jeg ikke mener at de skal. Hehe.

RSH: Det er klart. Men der er faktisk ikke så mange af dem i virkeligheden? De kommer typisk ind og er åbne overfor mulighederne?

ORTHOPEDIC SURGEON 3: Jamen der kommer jo. Det er så bredt et spektrum, at der kommer patienter ind hvor egen læge siger "jeg tror patienten har artrose", og til at patienten dårligt kan gå ikke. Så det er et meget bredt spektrum af patienter vi ser.

RSH: Ja.

ORTHOPEDIC SURGEON 3: Øhhm, men det er ikke så mange der kommer og, hvor de har fået at vide af den der nu sender dem ind, at de bare skal have et kunstigt knæ. Men der er nogle selvfølgelig.

RSH: Okay. Okay, så går vi videre til en ny overskrift der hedder, der handler om re-vurdering af det bedste behandlingstilbud på et bedre beslutningsgrundlag. Der mener jeg det med, at når du ser dem anden gang så har de prøvet konservativ behandling i form af træning. Om det gør det til en nemmere beslutning, eller om dit beslutningsgrundag er bedre når de ligesom har prøvet det her (træning)? Altså, hvorvidt operation er den bedste behandling eller ej?

ORTHOPEDIC SURGEON 3: Jeg kan i hvert fald sige til dig, at hvis patientens gener er uændret, og de stadig har så ondt at operationsindikationen er der, altså så er der i hvert fald ikke nogen tvivl om at det er den rigtige behandling. Hvis de ikke har haft nogen effekt af træning.

RSH: Så det ville gøre beslutningen nemmere, at de kommer ind anden gang og tingene (knæ-gener) har ikke ændret sig, men de har prøvet konservativ. Ville du så være mere sikker på din beslutning?

ORTHOPEDIC SURGEON 3: Jamen, altså man kan sige det sådan; jeg føler mig altid nogenlunde, altså jeg synes altid at jeg føler mig sikker på min beslutning. Selvfølgelig er der gråzoner, fordi du har patienter som radiologisk har knap så meget artrose, som dem med radiologisk svær artrose. Men man kan sige det sådan, at såfremt en patient har gennemgået snarere sagt al konservativ behandling og stadig væk har mange smerter, så er man i hvert fald på meget sikker grund. Men der er jo altid, man kan altid føle sig mere eller mindre sikker. Så jo mere konservativ behandling en patient har gennemgået, uden at der har været effekt af det, jo mere sikker er operationsindikationen. Og det her (quadx-1 træning) er jo et led i konservativ behandling. Så ja, det ville det være.

RSH: Yes. Hvad tror du patienterne tænker om det her med, at de ligesom re-vurderet efter at have prøvet konservativ behandling? Altså, at du fortæller dem "du er kandidat til operation, jeg synes du skal prøve træning først"

ORTHOPEDIC SURGEON 3: Jaaa, jeg vil sige sådan. Jeg har jo ikke haft ret mange patienter igennem det her. Det er jo ganske få, og jeg tror....

RSH: Det er også din hovedantagelse jeg gerne vil høre.

ORTHOPEDIC SURGEON 3: Hvad? Så skal jeg have spørgsmålet igen.

RSH: Jeg vil gerne høre hvad du tænker om det, selvom du ikke, altså dine tanker til det inden du har prøvet det særlig meget.

ORTHOPEDIC SURGEON 3: Jamen så stil lige spørgsmålet igen.

RSH: Hvad tror du patienterne tænker om, at de bliver tilbudt træning inden en evt. operation, når du har ligesom sagt til dem "du er kandidat men jeg synes du skal prøve træning først".

ORTHOPEDIC SURGEON 3: Jamen der er jo nogle patienter, som ikke vil (træne)? Også vil de jo hellere opereres, fordi det er jo det de er nået til, i det forløb de nu har været igennem ikke. Og andre...

RSH: Jamen hvis de ikke har prøvet træning før?

ORTHOPEDIC SURGEON 3: Ja ja, derfor kan der godt være patienter der godt vil operes ikke.

RSH: Jo.

ORTHOPEDIC SURGEON 3: For de kommer og har møg ondt.

RSH: Ja.

ORTHOPEDIC SURGEON 3: Øhm, jamen altså, jeg ved spilme ikke, nogle patienter tænker måske at det er en ekstra mulighed. At man prøver at træne sig ud af det, også for at kunne undgå og blive opereret. Det er vel den vej de vil se det. Fordi mange af patienterne, og os, og trods alt også bange for at blive opereret. Nogle vil helst undgå operation, hvis de kan. Så kan det måske være muligheden for dem.

RSH: Ja. Så det jeg hører det er, at det handler også lidt om patientens indgangsvinkel til det der hedder operation i virkeligheden?

ORTHOPEDIC SURGEON 3: Ja. Fordi det er jo forskelligt, kan man sige.

RSH: Ja. Så dem der er bange for eller forbeholdne overfor operation de vil typisk også være mere åbne overfor...

ORTHOPEDIC SURGEON 3: De vil måske være mere åbne overfor at prøve og træne sig ud af det, hvis de kan.

RSH: Ja. Og er det noget du oplever?

ORTHOPEDIC SURGEON 3: Jeg må indrømme jeg kan ikke huske nogle, men alle patienter er jo nervøse for at blive opereret.

RSH: Okay, ja. Så der er altid en...

ORTHOPEDIC SURGEON 3: Det er alle (bange for at blive opereret).

RSH: Ja, det er måske forståeligt nok. Ja, så vil jeg gerne høre lidt om hvad du tænker omkring det her med, at i og med at vi giver dem noget træning, og det er en øvelse, én, kun én øvelse, så det er relativt nemt at huske. Men vi giver dem ligesom et redskab, som de kan bruge til og håndtere deres knæ-symptomer. Hvad dine tanker er omkring, at vi ligesom uddanner patienten i selv og håndtere det?

ORTHOPEDIC SURGEON 3: Øhm, jamen det er vel fint. Hehe. Altså jeg mener, hvis det er noget patienten selv kan gøre, og det er en enkelt øvelse, og som de selv kan gøre derhjemme så er det jo fint. Alt hvad de kan gøre selv uden og få hjælp til det er jo fint. Men ulempen, ulempen, er at du ved ikke om de for gjort det. Og vi ved ikke om de for gjort det. Hvis de går ned i et træningscenter,

så har man mere kontrol med og ved om de for trænet eller ej, ikke. Også har de nogle til at skubbe på dem. Det er der også nogle der har brug for.

RSH: Men hele princippet i, at de for et redskab som måske hjælper dem, og de sådan set kan klare sig selv, den er du åben overfor?

ORTHOPEDIC SURGEON 3: Ja, det er fint. Jo mere de kan gøre selv, jo bedre er det.

RSH: Ja. Alright, så går vi videre til den næste her. Øh, og det drejer sig om, hvis vi er kommet herover i modellen, efter en evt. operation. Og det er om de har en potentiel bedre rehabilitering. Altså der er ligesom to muligheder, den ene den hedder "nogle vil måske udskyde deres operation", dvs. at de faktisk oplever en effekt (af træningen). Og det andet scenarie det er at de vælger stadig at blive opereret, men det løft de evt. har fået af træningen gør at deres rehabilitering er hurtigere eller nemmere. Hvad er dine tanker omkring de to scenarier?

ORTHOPEDIC SURGEON 3: Der er, jeg ved. Altså, der er måske nogle som kan få det bedre, som udskyder operationen. Det ved, det ser man jo. Om det så er træningen eller noget andet, det ved jeg så ikke. Og om det har effekt på, at du for en bedre rehabilitering, det ved jeg jo ikke noget om. Men det er da muligt.

RSH: Nej, men det er mere om. Hvis hypotetisk set...

ORTHOPEDIC SURGEON 3: Ja, hypotetisk set, så kan man da godt forestille sig det.

RSH: Ja, men synes du det er en god eller en dårlig ting?

ORTHOPEDIC SURGEON 3: Jamen, det er da kun godt, hvis man for bedre rehabilitering. Det er jo selvindlysende.

RSH: Ja. Hvad så med det her med, at de udskyder deres operation. Nu kalder vi det "udskyde" fordi, måske er det uundgåeligt at de skal opereres. Hvad synes du om det, er det spild af tid at udskyde det?

ORTHOPEDIC SURGEON 3: Jeg siger altid til patienterne, at hvis de kan leve med tilstanden, så er det bedre at vente til de ikke kan leve med tilstanden. Det er kun fint.

RSH: Okay. Så det ville være, i virkeligheden, en god ting jo længere man kan skubbe beslutningen "final treatment"?

ORTHOPEDIC SURGEON 3: Ja ja, absolut. Absolut. Det vil kun være godt. Altså, ideelt set så var det bedste hvis vi ikke operede patienterne. Forstå mig ret. Fordi der er altid en risiko. Så, så længe de kan leve med tilstanden så skal de jo ikke opereres. Men det er jo forskelligt for patienterne. Nogle kan leve med dét, og nogle kan leve med dét her. Så, men ideelt set så var det jo kun godt, hvis jeg ikke skulle operere dem. Fordi de kunne leve med det, som de havde det.

RSH: Ja, okay. Så dem der bliver opereret det vil altid være dem, hvor det er sidste løsning?

ORTHOPEDIC SURGEON 3: Ja, det er dem der siger, "Nu kan jeg ikke leve med det mere, jeg kan simpelthen ikke holde det ud. Livet er en jammerdal".

RSH: Ja, okay. Så er det det. Øhh, mens jeg lige tænker over om jeg har glemt noget, så er der noget jeg kan gøre for at du bedre husker at inkludere patienter, end det jeg allerede gør?

ORTHOPEDIC SURGEON 3: Næ, det tror jeg ikke. Altså, jeg tror også at jeg normalt altid husker det, men der er jo nogle af dem som har en "90", men de skal opereres, også er der jo ikke nogen grund til at kalde dig. Fordi de skal opereres, ikke.

RSH: Nej, og dem... Jo, er det fordi, at de så har prøvet konservativ allerede?

ORTHOPEDIC SURGEON 3: Det kan være fordi de har prøvet konservativ behandling, eller at de simpelthen har det så dårligt, at det bare er den eneste vej. Det er der nogle der er, simpelthen, ikke. Også ligesom mentalt inden de kommer, og via det de har været igennem, er det jeg siger "jeg skal bare opereres". Dem er der nogle af, det er jo langt fra alle, ikke. Men du kan ikke gøre andet end det du gør. Det er at du kommer, har highlighted 90, skrevet på sedlen også den seddel du kommer med. Så skal jeg nok huske det.

RSH: Ja. Altså, den patient du snakker om der. Jeg kan jo ikke lade være med at tænke, om de alligevel godt kunne blive inkluderet. Øh, fordi...

ORTHOPEDIC SURGEON 3: Mmmmhhh. Det er så min lægelige vurdering ud fra det de har været igennem, og den snak jeg har med patienten. Den snak jeg har med patienten. Det er jo altid en snak. Og det er jo ikke sådan, det er jo meget sådan, patienter er jo individuelle, jeg kan ikke putte dem i kasser, vel. Og nogle passer, nogle, for nogle er det bare det rigtige (operation). Men det er altid, man skal altid veje ting for og imod. Tale med patienten, finde ud af hvad er det for en patient, hvor er de henne, hvordan er deres hoved? Det er jo ikke kun, jeg opererer jeg ikke knæ, jeg opererer patienter.

RSH: Ja, jeg er helt med. Øh, og det, jeg vil jo, jeg er jo interessret i at få patienter med. Så det jeg i virkeligheden hører dig sige, det er at der er nogle af dem, de er ikke trænerbare?

ORTHOPEDIC SURGEON 3: Ja, joo, det kan godt være at man kunne træne dem. Men i deres hoved og med det de har været igennem, der er de bare nået dertil, at det er ikke en løsning for dem mere. Fordi at de er mentalt, og knæ-mæssigt, osv, derhenne hvor de skal opereres. Men det er igen også dem, det vil jo normalt være dem der har svær artrose.

RSH: Jamen jeg er jo interesseret i dem, der har svær artrose.

ORTHOPEDIC SURGEON 3: Ja, det ved jeg godt. Det ved jeg godt. Men sådan er der altså nogle patienter, hvor det er der de er.

RSH: Ja. Jeg tabte lige... Der var lige en god pointe der. Øh...

ORTHOPEDIC SURGEON 3: ...og det er jo en snak med dem om, hvor de er henne. Om træning overhovedet eller vil de forsøge noget andet. Det kan også være vægttab, altså. Nu ved jeg godt, det har ikke noget med dig at gøre, hvor det bare ikke er en mulig løsning for dem. De kan ikke, hvad

ved jeg, har prøvet over år.

RSH: Ja ja. Ja, med vægttabet?

ORTHOPEDIC SURGEON 3: Ja, f.eks. ikke.

RSH: Ja, jamen der var lige et eller andet før med træning og hvorfor de ikke skal tilbydes det. Men, det er fordi at det lyder på mig, som at der alligevel er nogel som, hvor de ikke ville være helt afvisende overfor det det, eller de ville godt kunne udføre det, men at du kigger på "det hele menneske" i virkeligheden, og vurderer at det vil ikke være en...

ORTHOPEDIC SURGEON 3: Også lytter jeg til dem. Hvor de er henne, ikke.

RSH: Ja, i deres liv eller tilstand med knæet?

ORTHOPEDIC SURGEON 3: Ja. Og hvad de har prøvet før.

RSH: Ja.

ORTHOPEDIC SURGEON 3: Og hvad, hvor de er henne.

RSH: Ja. Det er fordi jeg tænker. Hvis nu de er. De har prøvet at smertedække sig selv, de har prøvet at tabe sig, og de har måske prøvet at få nogle binyrebarkhormon-indsprøjtninger eller et eller andet, men de har faktisk ikke prøvet og træne. Så ville de jo stadigvæk være ret interessante.

ORTHOPEDIC SURGEON 3: Mmm, men det har de fleste af dem også. Men det kan godt være, at der er nogle af dem der ikke har prøvet og træne. Det kan godt være. Men det er jo et spørgsmål om, som jeg siger til dig, hvad har de inde i hovedet inden de kommer.

RSH: Ja.

ORTHOPEDIC SURGEON 3: Men jeg taler normalt altid træning med patienterne, atlså. Det er jo uanset hvad.

RSH: Okay. Så du spørger ind til om de har prøvet?

ORTHOPEDIC SURGEON 3: Ja, og hvad gør du. Nogle går i center, og har prøvet at gå, de har altid gået lange turer, nu kan de ikke mere. De kan ikke engang gå stavgang, gå med stokkke eller hvad ved jeg. Altså, hvor man ved. De har ligesom prøvet de ting. Og det behøver jeg ikke være, at de har gennemgået et formaliseret træningforløb hos en fysioterapeut. For man kan jo træne på mange måder.

RSH: Ja, korrekt. Er der noget vi ikke har været inde på, som du tænker du gerne vil sige ift. det her?

ORTHOPEDIC SURGEON 3: Nææ. Ikke udover at, vi har snakket om det her, træning er godt for

meget. Altså, sukkersygepatienter har godt af at træne. Hvis de for lidt sved på panden, øh, alt. Så, så træning er altid godt. Det tror jeg også jeg fik sagt.

RSH: Ja, det gjorde du. Så når du sælge træning nogle gange til en patient...

ORTHOPEDIC SURGEON 3: Det er en del. Det siger jeg altid til dem. Så siger jeg, et ting er knæet, men så er der resten, og det har også godt af at træne. Så der er mange, der er sideeffekter derudover, ikke. Det er den måde jeg siger til dem på. En ting er hvad vi gør ved knæet, men så er der også resten. Træning er godt for alt. Vægttab. Sukkersyge. Alt. Det er jo måden jeg sælger det på til patienterne, når jeg sætter dem til at træne. Det er sideeffekter.

RSH: Oplever du at det har en øget effekt? For de kommer jo typisk til dig med knæet.

ORTHOPEDIC SURGEON 3: Det kan jeg ikke måle. Det ved jeg ikke. Det kan jeg ikke måle, om det har en øget effekt.

RSH: Du kan ikke vurdere, om de lyser lidt op. Nå, jeg kan også tabe mig, jeg kan også...

ORTHOPEDIC SURGEON 3: Nej, det kan jeg ikke måle på.

RSH: Du har ikke en fornemmelse af om det...

ORTHOPEDIC SURGEON 3: Nej, det ved jeg ikke. Hehe.

RSH: Hehe, men der er vel en grund til at du gør det?

ORTHOPEDIC SURGEON 3: Jamen det er fordi, jeg tror det, men jeg ved det ikke.

RSH: Nej, okay. Du tror det motiverer dem?

ORTHOPEDIC SURGEON 3: Jeg tænker bare, at jeg kan motivere dem mere til og prøve og gøre det ved at sige sådan.

RSH: Ja.

ORTHOPEDIC SURGEON 3: Fordi, nogle gange er det også motivation, ikke, som er en hæmsko.

RSH: Jo. Ja, for træning?

ORTHOPEDIC SURGEON 3: Ja, altså. Nogle af dem er ikke vandt til at træne. Der er mange patienter i den aldersgruppe, som ikke er vandt til at træne. De har aldrig gjort det. Så det der med og. Altså, nu om dage er der massere der dyrker motion, også i 40erne og 50erne ikke. Men den generation, når de er i 60erne, der er mange af dem, der sku aldrig har dyrket motion. Så det er lidt sværere for dem tror jeg, mentalt.

RSH: Så det er i virkeligheden en barriere, tror du?

ORTHOPEDIC SURGEON 3: Ja, det tror jeg.

RSH: Fordi de aldrig har prøvet det simpelthen?

ORTHOPEDIC SURGEON 3: Ja, hvis man er vandt til at træne, eller løbetræne, eller man cyklede, eller hvad ved jeg, dyrke meget motion. Så er det jo meget nemmere, tror jeg, at gå ind i et træningsforløb. Ja, det tror jeg.

RSH: Så det er sådan en alder og kultur ting i virkeligheden der ligger lidt her.

ORTHOPEDIC SURGEON 3: Ja, det tror jeg. Men der er selvfølgelig også i min aldersgruppe nogle, der aldrig for sved på panden. Jeg tror, at hvis man har trænet tidligere, så tror jeg det er nemmere at gennemgå sådan et træningsforløb. Man har nemmere ved at motivere sig, tror jeg.

RSH: Ja. Okay, spændende. Jamen, øh, det var sådan set, vi har nået det hele igennem nu xx.

ORTHOPEDIC SURGEON 3: Det var godt.

RSH: Så lad os holde her.

ORTHOPEDIC SURGEON 3: Jep.

ORTHOPEDIC SURGEON 4

RSH: Jamen det første går på den her koordinering af konservativ og kirurgisk behandling før en operation, så det er det her med modellen. Så jeg kunne godt tænke mig at høre dine tanker om at kandidater til TKA tilbydes konservativ behandling i form af træning, inden en evt. operation?

ORTHOPEDIC SURGEON 4: Jeg tror mit udgangspunkt det er, at øh når de kommer ind, hvis jeg har set dem første gang, så er jeg ret hurtig til at spørge om de har fået øh, selvfølgelig hvad deres symptomer er, og i forhold til hvad vi skal gøre, hvor meget smertestillende spiser de og også om de har prøvet træning. Hvis de ikke spiser smertestillende, ikke har prøvet træning og har et ikke meget udpræget artroseknæ, så vil jeg altid sende dem til træning først. Så træning er øøh... - sidder meget løst hos mig. Og det er næsten obligatorisk, med mindre de kommer med noget som er så klokkeklart, hvor de har trukket den så langt, at det er nærmest knogle mod knogle, ellers er træning en del af forløbet hos mig.

RSH: Ja. Hos alle, mere eller mindre?

ORTHOPEDIC SURGEON 4: Ja.

RSH: Så det her, det er jo så en koordination af konservativ og kirurgisk behandling, så en fordel i det for dig, er det at du slipper for at operere en som måske vil have bedre gavn af noget andet først, eller hvordan?

ORTHOPEDIC SURGEON 4: For mit vedkommende er der ikke noget subjektivt i det med, at jeg slipper, jeg kan rigtig godt lide at operere og det er jo sådan det jeg laver og jeg synes det er rigtig sjovt. Men jeg synes da, det at prøve at finde ud af – hvem skal opereres af dem. Træning har, hvad kan man sige, har nok to formål for mit vedkommende, det ene er at jeg synes jeg oplever at mange af dem har det bedre, mange af dem kommer også, specielt i den lille grad af artrose, og siger at de faktisk har fået det bedre og det er, i hvert fald ikke for nuværende, er operation aktuelt. Det er typisk patienter som ikke har prøvet at træne så meget før og som heller ikke kender deres egen krop så godt og som ikke kan skelne mellem, hvad kan man sige, en manglende muskelstyrke, stivhed i og omkring led og så reelle artrose symptomer. Ligesom vi i virkeligheden heller ikke, måske selv kan det. Så for mig er det, et mål til at se – skal vi bruge kirurgi. Men det er nok også nogle gange ligeså meget et mål til at se hvad deres motivation er. Fordi, øh, hvis de overhovedet ikke har lyst til at træne og, så skal der gås anderledes til deres, måske til deres forventninger efter operation. Så jeg bruger det nok også ligeså meget til en, hvad skal man sige, personlighedsanalyse af dem. Rent post-træningsmæssigt også.

RSH: Ja. Så en ressourcescreening..?

ORTHOPEDIC SURGEON 4: Ja, jeg ville nok kalde det en ressourcescreening. Absolut.

RSH: Nu sagde du, at du havde oplevet umiddelbart, at det virker på dem der har mild/let artrose, har du noget indtryk af, har du prøvet at give træning til dem der har svær artrose?

ORTHOPEDIC SURGEON 4: Ja, jeg synes øh, at det afhænger af, til de svære artrose, afhænger det rigtig meget, er min oplevelse, at det afhænger relativt meget af deres øhh, personlighed også. Jeg synes de er, uanset hvad, er de meget mere afklaret både for deres eget vedkommende men også i forhold til at de skal, at den alliance der er mellem patienten og jeg, når vi har prøvet træning. Vi er et andet sted henne, vi kan også se hvordan artrosen den bølger lidt frem og tilbage for de fleste patienter, vi kan også se hvor vi er henne, vi har et mere stabilt mønster, så vi bedre kan lave en konklusion.

RSH: Altså fordi du simpelthen har fulgt dem over en længere periode?

ORTHOPEDIC SURGEON 4: Ja.

RSH: Okay.

ORTHOPEDIC SURGEON 4: Så til dit spørgsmål, jeg synes at det virker som om de har rigtig meget at vinde på relativ milde artrose karakterer, men når de kommer og har svære bevægeindskrænkninger, svære smerter, mere eller mindre konstante smerter, så oplever jeg at de ikke har, helt ligeså meget eller ligeså meget effekt af behandlingen, og det kan også være fordi at de har sværere ved at gennemføre behandlingen.

RSH: Ja. Det var jo nogle af fordelene ved at træne. Kan du tænke på nogle ulemper ved at tilbyde dem træning?

ORTHOPEDIC SURGEON 4: Nej. Det kan jeg faktisk ikke. Altså, jeg kan godt tænke på ulemper som, at de overholder ikke træningen, de er måske, hvis de er arbejdsdygtige, så går der yderligere et halvt år ved at prøve noget der måske ikke virker. Men jeg kan ikke som sådan i alliancen der hedder patient – behandling- kirurg, om det er mig eller nogen anden, så kan jeg ikke se nogen umiddelbart fordele ved ikke at prøve træning af. Baseret på at du får mulig gevinst, du får en bedre alliance og du får en fornemmelse af hvad deres motivation er for at træne.

RSH: Yes. Og det er jo så, det er meget for dig, vil det være de samme synspunkter du tænker der er for patienterne i det her, med at prøve træning af først?

ORTHOPEDIC SURGEON 4: Det vil jeg håbe. Men altså, det kan jeg jo dybest set ikke rigtig svare på. Men jeg synes relativt hurtigt, at jeg - tvinger ikke træning til nogle som ikke har lyst til det. Men langt de fleste når det beskrives, at vi laver træning, det virker på en stor del af patienterne, de skal samtidig måske spise noget smertestillende, men det er en begrænset periode med det formål, at de kan få trænet. Jeg tror hvis man stoppede der, så tror jeg det vil være demotiverende for dem, men når man siger " vi tager en kontrol om seks måneder", så ved vi hvor de er, så føler de også at de har en form af bagklods, for hvornår de skal komme igen og forklarer dem at det tager 4-6 måneder inden vi ser nogle resultater og så er de også indforstået med det. Men der er meget informationsarbejde i det her. I virkeligheden er det meget nemmere

ambulatoriemæssigt at sætte dem til operation, det kræver meget mere information at sætte dem til træning.

RSH: Ja, men en vigtig ting det er, det her med, at de får ikke af vide ”træning – vi ses ikke mere”, det er mere, at vi siger træning og så ses vi igen senere. Så det der med at de bliver holdt i hånden indtil et nyt punkt det er en vigtig ting?

ORTHOPEDIC SURGEON 4: Det tror jeg er meget meget vigtigt. Det er en meget vigtig milepæl i deres behandling, at de skal ses igen.

RSH: Yes. Så er der sådan en lavpraktisk ting. Det her, sådan som det her projekt er udformet, hvordan kan du se det passe ind i din daglige praksis?

ORTHOPEDIC SURGEON 4: Jamen jeg synes det passer ind. Ej, jeg vil sige, nu har jeg jo kontaktet dig en del gange hvor jeg tænkte kan det her være en kandidat, kan det ikke være en kandidat. Det er selvfølgelig en del af, at man lige finder ud af præcis hvad det er for nogle patienter. Nogle gange synes jeg at jeg har svært ved at passe det ind, fordi enten så har de været til træning og så er de motiveret for at få lavet kirurgi eller også så kommer de og de har ikke været til træning. Men hvis de ikke har været det, så er de ikke kandidat for det her program, hvis jeg tænker de skal træne før de skal opereres. Fordi det her skal jo kun være, hvis jeg tænker ”nu sætter jeg kniven” - og så tænker ”nej, det gør jeg ikke, jeg tager det her projekt”. Så der hvor de kommer og ikke har trænet, måske har spist noget smertestillende men hvor de skal til GLAD træning eller anden kommunal genoptræning, der er det her ikke et alternativ. Eller hvis jeg tænker, at de er for tykke, vi kan ikke operere dem nu, de skal tabe sig, der er det her heller ikke et alternativ. Så det er en relativ mindre del af patienterne som, hvor jeg synes det her passer til, hvis det defineres så hårdt som at, det skal være, altså jeg skal virkelig tænke ” nu sætter jeg kniven – ej det gør jeg ikke, jeg tager det her program”.

RSH: Så i virkeligheden, så fordi det skal være kandidater til kirurgi, så er der faktisk nogle overvægtige der ryger fra, fordi du vil ikke operere en der er for overvægtig, på grund af risikoer og så videre og manglende potentiel effekt før de har tabt sig?

ORTHOPEDIC SURGEON 4: Ja, hvis jeg ser dem første gang. Det kommer an på hvordan det har været. Hvis jeg ser dem første gang, at de har et BMI over 40, så siger jeg at jeg vil have et BMI under 40 og er rimelig, øh, er rimelig, hvad kan man sige, hård med det. Det betyder ikke at jeg ikke kan bløde op hvis der er forsøgt og der ikke er nogen effekt og de ligesom ikke kommer videre og deres bevægeindskrænkning bliver mere og mere, så de ikke rigtig, så vi ligesom låser os ind i en dårlig spiral. Men jeg er rimelig specifik omkring det der med at vægten, at fedme er et problem for operation. På alle parametre, både for patienten, både teknisk og de får meget beskrevet hvad den øgede risiko er og at det ikke er relateret til sådan den måde vi gør det her på stedet, men relateret til deres fedme. Men jeg tror ikke det var svar rigtigt på dit spørgsmål, du spurgte om jeg – jeg ikke bruger det her?

RSH: Ja. Det er mere der er jo sådan en pamflet af muligheder kan man sige, så er der GLAD, så er der operation og så er der lige pludselig også QUADX-1 og så, det er mere, hvem passer lige ned i denne her kasse, det hører jeg som at være en lille udfordring?

ORTHOPEDIC SURGEON 4: Ja. Det er også en udfordring for mig. Fordi, at de er i det forløb hvor de kommer, hvor jeg ser dem for eksempel første gang. Så siger jeg ” jamen du skal til træning”, jamen så er det jo ikke, så er det ikke det her jeg skal sende det til. Medmindre at jeg tænker ” jeg opererer dem med det samme”. Hvis jeg ligeså snart, at jeg tænker ”de skal til træning, før vi overhovedet skal tage stilling til om de skal opereres” - så er det ikke det her. Kommer de så seks måneder efter og har ingen effekt haft, så er det der man skal tænke- det her, men der er langt de fleste patienter ikke rigtig motiveret for så meget mere træning. Og hvis de har alle parametre i øvrigt i orden og det er noget som er frivilligt at indgå i det her, så øh.. eller alle øvrige parametre for operation, kirurgi. Så bliver det nok sådan at man slutter på at det er operation.

RSH: Ja. Dem, den patient der ville passe ind ville være den der kom ind og var kandidat og ikke havde prøvet træning endnu.

ORTHOPEDIC SURGEON 4: Ja. Men dem er der bare, i den måde jeg arbejder på, i hvert fald der er der færre af dem.

RSH: Ja.

ORTHOPEDIC SURGEON 4: Der ville det have været en fordel hvis man, for eksempel patienter der kommer fra almen praksis af, der er beskrevet ”har prøvet fysioterapi, har prøvet GLAD træning, har fortsat ondt, er interesseret i muligheder” og der ville man sige – det er vel perfekt, der kunne man sætte det der op.

RSH: Ja. Okay. Så er der sådan lidt mere en organisatorisk ting, hvem ville du sige har ansvaret for at det her det fungerer, altså at du kan sende nogle til QUADX-1 træning?

ORTHOPEDIC SURGEON 4: Ehm., det er… det har, altså der er det i ambulatoriedelen, hvor de bliver set i ambulatoriet, det jo sådan set, det er jo os der sidder med det, som kirurgerne der henviser og så er der selvfølgelig den del der foregår ude i kommunen. Jeg ser det ikke som om at nogen, på nogen måde har nogle større udfordringer end GLAD eller almindelig kommunal genoptræning.

RSH: Så det ville være den samme måde, at I skriver en henvisning og så kører det den der vej?

ORTHOPEDIC SURGEON 4: Ja. Fuldstændig. Det ville ikke være noget der overhovedet, altså udover at skulle skrive ”GLAD træning” eller udover at skulle skrive ”tages op på [utydelig tale]” hvis det skal bruges til nogle andre [utydelig tale] eller kommunal genoptræning så ville jeg bare skulle skrive ”QUAD-1 træning” i henvisningen, det var sådan set det.

RSH: Allright. Så går vi lige lidt videre til næste kategori, som handler om din rolle som ortopædkirurg og det er det her med rollen som, i den her præoperative, altså netop at det er træning før operation, hvor stor en rolle du spiller der, eventuel for, hvilke barrierer og nu [utydelig tale], om der er nogle barrierer hos dig, som ortopædkirurg, for at tilbyde træning modsat operation?

ORTHOPEDIC SURGEON 4: Neeej, jeg vil nærmere sige der er en modsat, der er barrierer for at tilbyde operation uden at have prøvet træning, derfor så, det giver mig på mange måder en beskrivelse af hvordan at patienten er. Hvis jeg er lidt i tvivl om hvordan, hvad der bliver det bedste og hvordan deres compliance vil være. Medmindre at de kommer med nogle klokkeklare, en klokkeklar årsag, har været udredt før, er været undersøgt før, bragende artrose og kan gå 50 meter. Jamen så ser jeg ikke at de skal have den store, have den store trænings..

RSH: Nej. Så den her fordom, jeg ligesom ligger op her, at "I er pro kirurgi og mod træning" det er i virkeligheden, som jeg hører det, så er træningen det er, som det også har været tidligere nævnt, det er i virkeligheden et redskab i at evaluere patienten til det bedste behandlingsforløb?

ORTHOPEDIC SURGEON 4: Ja. Det er det i hvert fald for mig.

RSH: Det er sådan du bruger det, ja. Kan du prøve og, igen, måske beskrive hvad du vil sige er en god kandidat til at gennemgå et træningsprogram inden en operation?

ORTHOPEDIC SURGEON 4: Ja, altså det her eller hvilket som helst træningsprogram?

RSH: Helst inden i denne her kontekst

ORTHOPEDIC SURGEON 4: Ja altså, den perfekte patient er den, som har været til træning, har spist smertestillende, fortsat ondt, er interesseret i om der findes anden mulighed end kirurgi.

RSH: Ja. Er der så, dem.. Hvis du så kan prøve at beskrive den patient, du mener, ikke vil have gavn af at træne, men som bør opereres med det samme?

ORTHOPEDIC SURGEON 4: Jeg tror at der er nogle patienter som øh, hvor man ikke kommer i mål og det er typisk patienter som kommer, patienter for eksempel med en udpræget comorbiditet, patienter der har forsøgt, eller patienter hvor compliance ikke er, eller hvor det ikke ligger i kulturen at de træner. Men hvor man så også forklarer dem at," jamen med dig er der en, på grund af comorbiditet, en øget risiko", " du kommer og siger at du gerne vil opereres, du har enormt ondt, dine symptomer er klassiske for artrose, du har enormt ondt og du sidder stille i sofaen. Og selvom jeg har sagt til dig at du ikke må sidde stille i sofaen, så sidder du stille i sofaen." Der ville jeg kunne sige " Vi opererer dig ikke fordi du skal ud og gå 18 huller på golfbanen, eller vi opererer dig ikke fordi du skal have et stort funktionelt niveau, vi opererer dig fordi du skal af med dine smerter og det accepterer vi, hvis du ikke kommer til og få et højt funktionelt niveau. Men det er simpelthen en smertebehandling, mere end en funktionel behandling at operere dig." De patienter ville jeg, ville jeg, ville jeg nok ikke...

RSH: Så det handler igen om…

ORTHOPEDIC SURGEON 4: Personligheds...

RSH: De to forskellige patienter her, dem der er god kandidat til træning og ham her der ikke er en god kandidat til træning, deres knæ kan sådan set godt være ens og deres symptomer kan være ens, men det jeg hører dig sige det er, at deres evne til at gøre noget andet end at få en operation er udslagsgivende for det der.

ORTHOPEDIC SURGEON 4: Ja. Det er meget en.. Men jeg vil tro det hedder en 90/10 fordeling, i hvert fald. Det falder meget ud til, at hvis de ikke har prøvet træning før, så ryger de stort set altid til træning.

RSH: Ja. Så den største hindring for at bruge træning systematisk i behandlingen af de her patienter, det er faktisk deres træningskultur eller motivation for det?

ORTHOPEDIC SURGEON 4: Ja.

RSH: Mere end noget andet.

ORTHOPEDIC SURGEON 4: Det synes jeg er, det synes jeg måske at de, eller at de har en.. Jeg vil sige der er ikke som sådan nogen, det er en minoritet hvor jeg tænker ” vi kommer ingen steder med træning her” eller at de kommer og siger ” jeg har så ondt at jeg ikke kan lave noget overhovedet” og ”jeg har forsøgt” eller.. Jamen okay, vi opererer dig på en smerteindikation, men igen, ikke på en funktionel indikation om at du skal ud og kunne lave rigtig meget. Det er en smertebehandling. Så det er en anden type patienter end den almindelige artrosepatient. Ved den almindelige artrosepatient der har jeg ikke nogen hinder for at sende dem, eller der har jeg ikke noget der gør at jeg ikke sender dem til træning først.

RSH: Nej. Så de patienter hvor det bliver en ren smertebehandling, der bliver de heller ikke sat i udsigt, at de skal kunne mere, andet end bare ikke have ondt, altså de bliver ikke..

ORTHOPEDIC SURGEON 4: Jeg stiller dem i udsigt, at vi stiler efter at du kan mere, men jeg siger med den, for eksempel hvis de kommer med en svær comorbiditet, eller de kommer [utydelig tale], så siger jeg til dem, ja så siger jeg, ”dels er den øget risiko ved kirurgi og dels er det ikke sådan at jeg med, hvad det kan være nedsat lungefunktion, nedsat hjertefunktion, at de kan regne med at hvis, at de nødvendigvis får et, et et voldsomt bedre funktionsniveau bagefter.

RSH: Nej.

ORTHOPEDIC SURGEON 4: Og det er jeg meget specifik med at forklare dem. Og at det er en risiko som er baseret på at deres, den historie de kommer med og de symptomer de kommer med i forvejen, at der kan vi ikke løfte det op til at de får en normal baggrundsbefolknings funktion.

RSH: Ja. Godt. Så går vi videre til næste kategori og det handler om, så hvis vi kigger på modellen, så er vi så øh herovre, så det er når de kommer tilbage efter at have fået træning.

Når I snakker med dem på det tidspunkt, så det er sådan en revurdering af "hvad er det bedste behandlingstilbud til dig nu. - Er det at fortsætte træning fordi du oplever at det er tilstrækkeligt for dig i forhold til smerte og funktion eller øh har det ikke haft en tilstrækkelig effekt og er operation måske det vi skal gøre for dig her." Så øh, det der er tanken i projektet det er, at når I tager den snak herovre, så skal det være nemmere at tage beslutningen om kirurgi fordi de har været igennem træningsforløbet. Så mener du, at det er med til at give jer et bedre og bredere beslutningsgrundlag for at tage beslutningen?

ORTHOPEDIC SURGEON 4: Ja. Det synes jeg absolut det er. Jeg vil sige at inden, når jeg sender dem til træning første gang jeg ser dem, der er det nok meget min egen beslutning, der er det klart en fornemmelse af eller der er det både en behandling, men også ligeså meget for at jeg får en, jeg får selv en - lidt en fornemmelse af hvor patienterne er i det halve år de træner, om hvordan deres compliance er. Når de kommer tilbage her, så vil jeg høre dem, " Hvordan er det gået? Synes du at der er en fremgang, er det det samme? Har du trænet? Hvor meget har du trænet? Øh, Spiser du mindre smertestillende? Fordi det kan godt være at de siger "jamen jeg har ligeså ondt", men til gengæld spiser de ingen smertestillende. Jamen så har de jo formegentlig ikke ligeså ondt. Så ligesom prøve at penetrere, okay har der været en effekt af det. Når der så har været en effekt af det, så er jeg nok mere, så lægger jeg, hvad kan man sige, beslutningsgrundlaget lidt mere ind i mellem os, patienterne selv. Siger " jamen hvor synes du at vi er henne nu? Skal vi operere? Skal vi fortsætte med træning? Er du motiveret for det?" Øh, og der øh, det synes jeg er, det er min oplevelse, at der er mange af dem ret afklaret for hvor det står henne.

RSH: Okay. Så det bliver også nemmere i virkeligheden og have en dialog..

ORTHOPEDIC SURGEON 4: Meget nemmere

RSH: .. Fordi der er flere valgmuligheder

ORTHOPEDIC SURGEON 4: Meget nemmere. Og mange af dem kommer, synes jeg, jeg tror de kommer og er overrasket over hvor godt de har det. Og dem vil jeg jo have, dem vil man jo, synes jeg i min verden, have opereret på en måske for løs indikation.

RSH: Ja. Så i virkeligheden, så kan træningen, oplever du, være med til at nogle du, hvis du var lidt hurtig, havde opereret i virkeligheden fandt ud af, at det var måske meget godt at vi ventede med den beslutning fordi..

ORTHOPEDIC SURGEON 4: Men det tror jeg ikke er sådan jeg oplever, at der er nogen kirurger der er hurtige, jeg synes at det, jeg synes at det er en generel ting at øh, at træning har et øh. Vi spoler tilbage, jeg tror vi måske ser forskelligt, jeg tror ikke vi alle sammen ser de samme patienter. Jeg tror der er nogen der måske ser nogle patienter som, det kan være en kirurg ser nogle typer patienter som måske skal opereres tidligere eller nogen der ser nogen, ja nogle patienter der kører meget efter, hvad kan man sige, standard protokollen: træning først, operation bagefter. Men af dem jeg ser, som kommer tilbage og siger " jeg skal ikke opereres", der er der en vis del der har enormt god effekt af træning.

RSH: Ja. Det er jo klare fordele ved det her, har du oplevet nogle ulemper?

ORTHOPEDIC SURGEON 4: Nej

RSH: Ved at tilbyde dem træning?

ORTHOPEDIC SURGEON 4: Nej. Nej det synes jeg ikke at jeg har. Der kan være nogle gange nogen der kommer og siger "det virker ikke" og så videre og når man så spørger dem hvor meget de har trænet eller hvad de kunne, så – jeg vil sige, at jeg synes at det er de færreste som har kørt et 6 måneders regelret forløb, som ikke har haft nogen gavn af det. Langt hen ad vejen der skyldes det at, "nåh men der gik for lang tid inden kommunen skrev" eller "jamen jeg har kun fået trænet 2 gange om måneden" eller. Og så ved jeg jo hvor de er henne og så kan jeg også med ret sige, jamen altså hvis vi skal operere dig, så er du simpelthen nødt til at vise højere moral. Fordi jeg plejer måske at sige at det kan være, og det er der også noget retorisk i at jeg siger, "jeg kan lave 50 % af arbejdet, jeg kan lave noget god kirurgi, men hvis øh, de resterende 50 % det tror jeg afhænger rigtig meget af din arbejdsindsats efterfølgende". Det er jo et arbitrært tal men det er for at give dem en fornemmelse af hvor meget de selv er inde over. Og øh, man kan sige, så kan jeg måske sige, "du kan se at du har haft nogen effekt af træning", hvis de har haft det, kan jeg sige "du har ikke trænet særlig regelmæssigt, det er svært at sige hvad vi, det er svært for mig ligesom og sige hvor du er henne med hensyn til din lyst, motivation, compliance til at lave det her efterfølgende." Og det er en måde at sige, og så kan det være at det går op for dem, at sige " ja det kan jeg faktisk godt se, men jeg vil rigtig gerne opereres og jeg skal nok give den en ekstra indsats".

RSH: Ja. Så det er også et værktøj til at fortælle dem, at det ikke er sådan en quick fix, der er sgu også en arbejdsindsats bagved?

ORTHOPEDIC SURGEON 4: Ja. Det er meget øh. Det bruger jeg det meget til.

RSH: Yes. Så nu var du selv lige inde på det er hvaa.. Jeg kunne tænke mig at høre hvad du tror patienterne tænker om det. Altså, du har lige været lidt inde på det.

ORTHOPEDIC SURGEON 4: Jeg tror det er meget blandet. Jeg tror mange. Der kommer nogen, som helt fra, at de har ikke lyst til at gennemgå kirurgi og tænker "det er dejligt at der findes et alternativ" og som tror på det. Der kommer nogen der tænker "jeg har prøvet fysioterapi", men det har de ikke rigtigt. Altså, de har siddet og hevet i nogle maskiner for overarmen, for overkroppen. Så kommer der nogle som er ekstremt motiverede og så kommer der en del øh.. hvor, at de faktisk i virkeligheden har haft dårlig kropskontakt, så jeg tror ikke de ved hvad fysioterapi indebærer. Der tror jeg vi har et rigtig stort arbejde foran os med den, hvad skal man sige, etniske minoritet eller minoriteter, fordi jeg tror mange af dem ikke, ikke har den idé om at man skal træne, at træning er en del af udredningen og behandlingen.

RSH: Ja det synes jeg også, at jeg oplever. Med de par stykker, der er med anden etnisk baggrund, der kommer med og de har også mere ondt.

ORTHOPEDIC SURGEON 4: Ja, de har i hvert fald nogle andre typer smerter. Og det kan også være at de gradere det. Det er svært at sige om de har mere ondt, de gradere det nok selv på en anden måde. Jeg tror det er mere en plus/minus, altså enten så har de meget ondt eller ikke ondt. Der er ikke en graderet skala.

RSH: Nej, okay. Så går vi videre til at snakke lidt om den øvelse de får. Fordi gennem den her intervention som de får her, så får de jo en øvelse, som er meget simpel og man kan justere den ret simpelt derhjemme, ved at rykke en stol frem og tilbage og så øger man spændingen eller mindsker spændingen i elastikken. Øh, og på den måde får de så et værktøj, som de selv kan hive frem, hvis de har meget ondt i knæet i perioder for eksempel. Man kunne godt forestille sig at nogle de gennemgår det her og de har effekt og så når projektet er slut, så glemmer de det og lægger den måske væk og så kommer deres symptomer tilbage. Og så i virkeligheden, det er selvfølgelig meget teoretisk det her, men så bør de kunne hive den frem igen, træne igen og opleve effekten endnu en gang. Så det vil jeg.. Hvad er dit syn på det her med, at man ligesom uddanner patienten til selv at håndtere?

ORTHOPEDIC SURGEON 4: Det er et meget tiltalende koncept. Øh, jeg tror meget det afhænger af om patienten har, hvad skal man sige, ejerskab af sin sygdom. Dem der har ejerskab, det er også dem der er motiverede. Dem der er motiverede, de kan se formålet. Dem der kan se formålet, de gør det. Dem der ikke får gjort det og ikke har ejerskab og føler at de bare gerne vil komme op til lægen/kirurgen eller hvad det er, få en pille, få en gang kirurgi, der er helt klart en anden udfordring. Så her er det igen de motiverede og de ikke- motiverede patienter, som vi måler på.

RSH: Ja. Og det vil jo altid i sådan et træningsprojekt, der vil det formegentlig altid være en bias, at der er en motivationsfaktor hos dem der deltager her, ikke?

ORTHOPEDIC SURGEON 4: Ja.

RSH: Men ideen om at man kan give dem det her redskab selv, så de også måske har mindre kontakt til sundhedsvæsenet, fordi de selv kan klare det..

ORTHOPEDIC SURGEON 4: Det er en fan.. Ideen er jo rigtig god og jeg tror også det hjælper for en vis del.

RSH: Så du har tro på at det virker..

ORTHOPEDIC SURGEON 4: Ja ja, jamen jeg er sådan ikke i tvivl om..

RSH: At det er en mulighed hos motiverede mennesker det her.

ORTHOPEDIC SURGEON 4: Ja. Nåh men jeg øh.. Altså træning som sådan. Jeg har jo, det skal jeg jo sige her, jeg har jo ikke øh, jeg har jo ikke haft, jeg har ikke haft patienter igennem selv, som har kørt lige præcis det der QUADX-1. Øh, så jeg har ikke set dem før og efter. Det er jo min øh, der er jeg jo lidt. Men ideen om at de har et, om det så er GLAD trænings set-up derhjemme, de kan lave derhjemme. Eller om det er det her med en øvelse. Hvis at det viser sig at have en god effekt,

jamen så er det absolut. Ideen om at uddanne patienterne er jo, øh den forebyggende behandling er jo øh, helt fænomenal.

RSH: Ja. Øh, kan du se nogle ulemper ved det?

ORTHOPEDIC SURGEON 4: [pause] Jeg kan kun se den ulempe der er hvis det er en masse ressourcer, som vi bruger på noget hvor patienterne ikke gennemfører det alligevel. Altså igen, et eller andet kvalitetsprojekt, som bliver som sådan et eller andet, øh, fyld, øh hvor der ikke kommer øh, hvor patienterne ikke laver det, de føler sig ikke udannede, så der går i virkeligheden bare nogle mennesker rundt og informerer om noget som ikke rigtig kommer til og øh - at patienterne bruger det rigtigt. Det kunne være, det kunne være, det kunne være det eneste jeg tænkte.

RSH: Ja, så det simpelthen, så man, øh, så man valgte at bruge det og så endte det med at patienterne slet ikke lavede noget derhjemme og så var det spild af..

ORTHOPEDIC SURGEON 4: Nej, eller det bliver ikke rigtig brugt eller ikke rigtig introduceret eller øh. Altså, så det er det eneste, men det er jo som så meget andet. Det er jo ikke, det er jo ikke, det er jo ikke konceptets problem. Det jo et formidlingsproblem.

RSH: Det er implementeringen der skal være gennemført ordentligt..

ORTHOPEDIC SURGEON 4: Ja. Så det er det eneste jeg kan se.

RSH: Så når vi til sidste punkt. Og det er at øh. Der er én effekt af det her og det er at nogle af dem, de vil måske vælge at udskyde valget om en operation, hvis de oplever effekt af det og så er der det, at dem der stadig vælger at få en operation, at de måske går en nemmere rehabilitering i møde, qua at de har fået et løft ved at træne før operationen.

ORTHOPEDIC SURGEON 4: Ja. Ja.

RSH: Så de her to scenarier, at nogle vælger at udskyde og nogle de går måske en bedre rehabilitering, lettere rehabilitering i møde. Hvad har du, kan du se nogen øh, hvad er dine tanker om de to ting?

ORTHOPEDIC SURGEON 4: Jamen der er jo kun øh. Jeg kan kun sige at det synes jeg er, hvis det er sådan at den sidste del- er jo lidt usikkert om det reelt løf.., om det reelt gør det lettere for dem. Men jeg tror i forbindelse med forventningsafstemning, for mit vedkommende, i forhold til patienten, der øh, der synes jeg det er en meget positiv måde at arbejde sammen på. Jeg synes det handler om at de får medejerskab. Overordnet handler det om at de får medejerskab. At de har en stor betydning for outcomet, deres træning, deres levevis, ind i det her snakker vi jo også – ryger de? Hvad spiser de? Hvordan er deres levevis, måske også lidt – hvordan sover de? Hvad er nødvendigt at man fokuserer på bagefter. Øh, så give dem.. Jeg synes alt det med træning og den vej rundt, handler meget om medejerskab.

RSH: Ja. Øh, hvad var det jeg tænkte på.[Pause] Nå, den røg lige. Så er der sådan lidt afsluttende her, om der er noget, det er sådan lavpraktisk i forhold til projektet, om jeg kan gøre noget andet for, at det er nemmere for dig at huske at inkludere patienterne?

ORTHOPEDIC SURGEON 4: Ja, jeg synes det er rigtig fint at du skriver på de der patienter som øh.. Som at du.. Det synes jeg er rigtig fint at du gør. Og jeg tror bare, at lige præcis det her studies karakter, det er jo det der med – at nogle gange vil der være patienter, eller ofte vil der være patienter der kommer tilbage, så er de meget motiveret for kirurgi, de har gjort det de skulle – det har ikke haft effekt. Og de er meget motiverede for kirurgi og det er måske den arbejdsdygtige alder. Og de har svært ved at gennemføre deres arbejde, så der synes jeg at jeg har svært ved at sige, ”Vi..”, og eftersom det er et studie, så kan man ligesom ikke, man kan jo ikke, man kan ligesom ikke tvinge dem over i det kan man sige, vel?

RSH: Det kan man ikke, nej.

ORTHOPEDIC SURGEON 4: Så øh, der vil jeg nok være tendens til at tilbyde dem operation. Så det er rent praktisk med inkludering af patienter. Der vil der være, der er der lige en, der er en stor patientkategori der ryger ud.

RSH: Ja ja. Men det er mere sådan, altså så det med at jeg har skrevet det der telefonnummer og sådan noget, det..?

ORTHOPEDIC SURGEON 4: Det er super.

RSH: Det er en fin måde det fungerer på?

ORTHOPEDIC SURGEON 4: Ja. Det er en super måde det fungerer på.

RSH: Har du nogle, et eller andet, en kommentar til noget vi har været inde på?

ORTHOPEDIC SURGEON 4: Nej. Jeg synes øh. Jeg synes det. Jeg ville jo gerne inkludere flere til dit studie, men altså det er der hvor den er lige nu.

RSH: Ja. Det kommer.

ORTHOPEDIC SURGEON 4: Det kommer.

RSH: Allright. Tak xx.
